# Analysing the nutrition-disease nexus: the case of malaria

**DOI:** 10.1186/s12936-015-0894-x

**Published:** 2015-12-01

**Authors:** Milinda Lakkam, Lawrence M. Wein

**Affiliations:** Institute for Computational and Mathematical Engineering, Stanford University, Stanford, CA 94305 USA; Graduate School of Business, Stanford University, Stanford, CA 94305 USA

**Keywords:** Malaria, Undernutrition, Bed nets, Mathematical models

## Abstract

**Background:**

Motivated by the observation that children suffering from undernutrition are more likely to experience disease and are more likely to die if they do contract a disease, mathematical modelling is used to explore the ramifications of targeting preventive disease measures to undernutritioned children.

**Methods:**

A malaria model is constructed with superinfection and heterogeneous susceptibility, where a portion of this susceptibility is due to undernutrition (as measured by weight-for-age z scores); so as to isolate the impact of supplementary food on malaria from the influence of confounding factors, the portion of the total susceptibility that is due to undernutrition is estimated from a large randomized trial of supplementary feeding. Logistic regression is used to estimate mortality given malaria infection as a function of weight-for-age z scores. The clinical malaria morbidity and malaria mortality are analytically computed for a variety of policies involving supplementary food and insecticide-treated bed nets.

**Results:**

The portion of heterogeneity in susceptibility that is due to undernutrition is estimated to be 90.3 %. Targeting insecticide-treated bed nets to undernutritioned children leads to fewer malaria deaths than the random distribution of bed nets in the hypoendemic and mesoendemic settings. When baseline bed net coverage for children is 20 %, supplementary food given to underweight children is estimated to reduce malaria mortality by 7.2–22.9 % as the entomological inoculation rate ranges from 500 to 1.0. In the hyperendemic setting, supplementary food has a bigger impact than bed nets, particularly when baseline bed net coverage is high.

**Conclusions:**

Although the results are speculative (e.g., they are based on parameter estimates that do not possess the traditional statistical significance level), the biological plausibility of the modelling assumptions and the high price-sensitivity of demand for bed nets suggest that free bed net distribution targeted to undernutritioned children in areas suffering from both undernutrition and malaria (e.g., sub-Saharan Africa) should be the subject of a randomized trial in a hypoendemic or mesoendemic setting.

**Electronic supplementary material:**

The online version of this article (doi:10.1186/s12936-015-0894-x) contains supplementary material, which is available to authorized users.

## Background

For many transmissible diseases, large individual variation in infectiousness [1] or susceptibility can be predicted *a priori*, which allows for effective targeting of preventive interventions, such as condom education for sex workers [2], needle exchange for injecting drug users [3], and influenza vaccinations in elementary schools and nursing homes [4]. However, for children under 5 years old in Africa, where pneumonia, malaria and diarrhoea cause 17, 15 and 12 % of deaths, respectively [5], identifying groups of highly susceptible children for targeted preventive interventions is less obvious than in the examples above. Motivated by the fact that undernutrition underlies ${\approx}45\,\%$ of deaths in children under five [5], this study assesses the potential of using an easily observable undernutrition metric—the weight-for-age z score (WAZ)—to target preventive resources in the case of malaria, which has a less diffuse cause than the other two diseases (e.g., diarrhoea can be caused by viruses, bacteria or parasites).

Two preventive resources are considered: food and insecticide-treated bed nets (ITNs). Although ITNs are effective at preventing malaria [6], demand is highly price-sensitive and is greatly reduced when ITNs are not free [7, 8], making them attractive candidates for free targeted intervention. The World Health Organization recommended a switch from targeted ITN intervention (e.g., for children under five and pregnant women) to universal ITN coverage in 2008 [9], which led to the distribution of over 450M ITNs in sub-Saharan Africa between 2008 and 2012 [10]. Nonethless, malaria control funding peaked in 2012 and has begun to decline, with funding commitments in 2013–2016 estimated to meet just over half of demand, yielding a funding gap of $\approx$374M ITNs [10]. In this resource-constrained setting, the targeting of ITNs may need to be reconsidered [11].

To assess various targeted and untargeted intervention strategies, a mathematical model is needed that captures the key interactions between nutrition and infection [12]. Some data suggest that (1) children with low nutrition are more susceptible to disease [13], (2) infection decreases a child’s nutritional status [14–16], and (3) undernutrition increases mortality among infected children [13]. Several other interactions are also possible: (4) undernutritioned children have longer infectious periods [17], (5) infection reduces the effectiveness of nutritional interventions, and (6) undernutrition lessens the impact of infection control measures. However, very little or no evidence support these phenomena [12]. An existing malaria model with heterogeneous susceptibility [18] is generalized so as to incorporate interactions (1) and (3) [interaction (2) is discussed later]. This is achieved by assuming that a portion of the individual variability in susceptibility is due to undernutrition (as measured by WAZ), and assuming that the death rate of infecteds depends on a child’s WAZ. After the model is calibrated using existing data [13, 18, 19], it is used to estimate the impact on clinical malaria morbidity and malaria mortality from various policies that provide food and/or ITNs to children from ages 6 months to 5 years with low WAZ.

## Methods

### The $\int$SI$^{\circ }$S model

The general model is built in several steps and the model parameters and their values are listed in Table 1. The starting point is the $\int$SI$^{\circ }$S model in [18], which was found to provide the best (among six models) overall fit to malaria data for African children under 15 years old from 91 communities. This model relates the entomological inoculation rate (EIR), which is the number of bites from an infectious mosquito per year per person, and the proportion of people who are infected with *P. falciparum*, and allows for a heterogeneous infection rate and super-infection (i.e., no immunity to re-infection). Let *S* be the random susceptibility within the child (ages 6 months to 5 years) population, which has probability density function (PDF) $f_S(s)$ that is a gamma with mean 1.0 and variance 1/*k* (i.e., shape parameter *k* and scale parameter 1/*k*), denoted by $S\sim \Gamma (k,1/k)$. A child with $S=s$ has infection rate $bs\mathcal{E}$, where *b* is the probability that a bite from an infectious mosquito leads to an infection and $\mathcal{E}$ is the annual EIR. Under super-infection (and assuming that infections clear independently), the clearance rate is $g(bs\mathcal{E},r)$, where $g(\Lambda ,r)=\Lambda /(e^{\Lambda /r}-1)$, and 1/*r* is the expected time to clear each infection. Let *x*(*s*) be the proportion of children with susceptibility *s* that are infected. The dynamics of *x*(*s*) is described by the differential equation [Eq. (4) of [18]]

1 $$\begin{aligned} \dot{x}(s) = bs\mathcal{E}(1-x(s)) - g(bs\mathcal{E},r)x(s). \end{aligned}$$

**Table 1:** Parameter values

Parameter	Description	Value
$a(\phi _N)$	Biting rate	(58) and Additional file 1: Figure 1 [20]
*b*	Proportion of bites that produce human infection	$b/r=0.57$ year [21]
*r*	Human clearance rate for each infection	$b/r=0.57$ year [21]
$\alpha$	Proportion of children to adults	0.17 [22]
$m(\phi _N)$	Ratio of female mosquitoes to humans	(59) and Additional file 1: Figure 2 [20]
*c*	Proportion of bites that produce mosquito infection	0.5 [23]
$\mu (\phi _N)$	Mosquito mortality rate	(60) and Additional file 1: Figure 3 [20]
*k*	Susceptibility shape parameter	0.17 [21]
$k_1$	Undernutrition shape parameter	0.153 [19], Additional file 1: Figure 4
$\mu _Z, \sigma _Z$	Normal WAZ parameters	$-$1.00, 1.27 [24]
$\mu _A, \sigma _A$	Lognormal food parameters	$-$1.56, 0.42 [19]
$p_c(\mathcal{E})$	Proportion of infections that are clinical	(74), Additional file 1: Figure 6 and §2.4
*d*(*z*)	Malaria mortality per 1000 children	$e^{1.851-0.607z}$ [13, 25], Additional file 1: Figure 8

### Incorporating adults and infected mosquitoes

Equation (1) needs to be generalised to allow the mosquito population to vary over time so as to be able to look at the impact of interventions. Following the traditional Ross model [26], let *y* be the proportion of mosquitoes that are infected, *a* be the biting rate, *c* be the proportion of bites by a susceptible mosquito of an infected human that lead to the mosquito getting infected, $\mu$ be the mosquito mortality rate, and *m* be the number of female mosquitoes per human.

Although the analysis focuses on children, adults need to be incorporated into the model because they are a key reservoir for infection. Let *w* be the proportion of adults that are infected, *h* be the proportion of humans (children and adults) that are infected, and $\alpha$ be the percentage of the population under 5 years of age. Assuming that the adult population has homogeneous susceptibility and noting that $\mathcal{E}$ corresponds to *amy*, the model in the absence of nutrition and interventions is

2 $$\begin{aligned} \dot{x}(s)&= bsamy(1-x(s)) - g(bsamy,r)x(s), \end{aligned}$$

3 $$\begin{aligned} \dot{w}&= bamy(1-w) - g(bamy,r)w, \end{aligned}$$

4 $$\begin{aligned} \dot{y}&= ach(1-y)-\mu y, \end{aligned}$$

where

5 $$\begin{aligned} h = \alpha \Bigl (\int _0^{\infty }x(s)f_S(s)ds\Bigr ) + (1-\alpha )w. \end{aligned}$$

### Accounting for baseline ITN coverage

Baseline (i.e., pre-intervention) ITN coverage is accounted for by dividing the children into two sub-populations, one with ITNs and one without. Let $\phi _0^c, \phi _0^a$ represent the baseline ITN coverage for the child and adult population respectively, and assume the baseline allocation for children is independent of their WAZ scores. Assuming that one adult gets ITN coverage for every child under the net (in Fig. 4.2 in [27], the ITN coverage for children under five is very similar to the ITN coverage for women) implies that $\phi _0^a = \frac{\alpha }{1-\alpha } \phi _0^c$.

A simplified version of the feeding cycle model with ITNs in [20] is used, which allows the biting rate *a*, the mosquito-human ratio *m* and the mosquito death rate $\mu$ to be functions of the ITN coverage in the human population, which is $\phi _N = \alpha \phi _0^c + (1-\alpha ) \phi _0^a$; these functions, which are now denoted by $a(\phi _N)$, $m(\phi _N)$ and $\mu (\phi _N)$, are specified so that they match the model output in [20], as described later. In addition, let *p* be the probability that a mosquito finding an ITN-protected human is able to successfully bite him. Let $x_1(s)$ represent the proportion of unprotected children with susceptibility *s* that are infected and $x_2(s)$ be the proportion of ITN-protected children that are infected. Similarly, let $w_1$ and $w_2$ represent the proportion of adults infected without and with ITN protection, respectively. The model, accounting for baseline ITN coverage, is

6 $$\begin{aligned} \dot{x}_1(s)&= bsa(\phi _N) \,m(\phi _N) \,y(1-x_1(s)) - g(bsa(\phi _N) \,m(\phi _N)y,r)\,x_1(s),\end{aligned}$$

7 $$\begin{aligned} \dot{x}_2(s)&= bsa(\phi _N) \,m(\phi _N) \,py(1-x_2(s)) - g(bsa(\phi _N) \,m(\phi _N)py,r)\,x_1(s),\end{aligned}$$

8 $$\begin{aligned} \dot{w}_1&= ba(\phi _N)\, m(\phi _N) \,y(1-w_1) - g(ba(\phi _N) \,m(\phi _N)y,r)\,w_1,\end{aligned}$$

9 $$\begin{aligned} \dot{w}_2&= ba(\phi _N)\, m(\phi _N) \,py(1-w_2) - g(ba(\phi _N) \,m(\phi _N)py,r)\,w_2, \end{aligned}$$

10 $$\begin{aligned} \dot{y}&= a(\phi _N) \,ch(1-y)-\mu (\phi _N)\,y, \end{aligned}$$

where

11 $$\begin{aligned} h = \alpha \left( ( 1- \phi _0^c) \int _0^{\infty }\,x_1(s)\,f_S(s)\,ds + p\phi _0^c \int _0^{\infty }\,x_2(s)\,f_S(s)\,ds \right) + (1-\alpha ) \left( ( 1- \phi _0^a) \,w_1 + p\phi _0^a \,w_2 \right) \end{aligned}$$

is now interpreted such that $a(\phi _N)h$ is the biting rate of infected humans.

### Incorporating nutrition

To incorporate nutrition, let the susceptibility *S* equal $U+V$, where *U* is a measure of undernutrition and *V* is the residual portion of susceptiblity that does not depend on undernutrition. Assume that *U* and *V* are statistically independent with PDFs $f_U(u)$ and $f_V(v)$ and cumulative distribution functions (CDFs) $F_U(u)$ and $F_V(v)$. So as to estimate only one additional parameter, it is assumed that $U\sim \Gamma (k_1,1/k)$ and $V\sim \Gamma (k-k_1,1/k)$, which is consistent with $S\sim \Gamma (k,1/k)$. Let $Z\sim \mathcal{N}(\mu _Z,\sigma _Z)$ denote the random WAZ values in the child population, which has PDF $f_Z(z)$ and CDF $F_Z(z)$. Because higher values of $S=U+V$ and lower levels of *Z* each lead to higher susceptibility, a one-to-one transformation is constructed between *U* and *Z* such that the $p\mathrm{th}$ fractile of the distribution of *U* corresponds to the $(1-p)\mathrm{th}$ fractile of the distribution of *Z* for all $p\in [0,1]$; this transformation is $u(z)=F_U^{-1}(1-F_Z(z))$, which is displayed in Additional file 1: Figure 8.

### Interventions

Four intervention policies are considered (Table 2), which all assume prior ITN coverage of $\phi _0^c$ and $\phi _0^a$ for children and adults: a targeted (i.e., based on WAZ $<\theta$) food policy, an untargeted (i.e., random distribution with an additional coverage of $\phi$ in the unprotected population) ITN policy, a targeted ITN policy, and a targeted food and targeted ITN policy. The impact of supplementary food is to change a child’s WAZ from *z* to $z+A$, where *A* has a lognormal distribution with PDF $f_A(a)$ and $\ln A\sim \mathcal{N}(\mu _A,\sigma _A)$.

**Table 2:** Intervention policies

Policy	Description	Parameter Values
No intervention	No food or ITN	$\phi _0^c$ fixed, $\phi _t^{(1)} = \phi _t^{(2)} = 0$,
		$$\phi _a^1 = \frac{\alpha }{1-\alpha } \phi _0^c, \phi _N = 2\alpha \phi _0^c$$,
Targeted food	Food if WAZ $< \theta$	$p_I=1$, $\phi _0^c$ fixed, $\phi _t^{(1)} = \phi _t^{(2)} = P(Z<\theta )$,
		$$\phi _a^1 = \frac{\alpha }{1-\alpha } \phi _0^c, \phi _N = 2\alpha \phi _0^c$$,
		$$P(G=11|Z=z, G_0 =1)= 1_{\{z>\theta \}}$$,
		$$P(G=12|Z=z, G_0 =1)= 1_{\{z<\theta \}}$$,
		$$P(G=21|Z=z, G_0 =2)= 1_{\{z>\theta \}}$$,
		$$ P(G=22|Z=z,G_0=2)=1_{\{z<\theta \}}$$
Untargeted ITN	ITN with probability $\phi$ if unprotected	$p_I = p, \phi _0^c$ fixed, $\phi _t^{(1)} = \phi , \phi _t^{(2)} = 0, f_A(a)=\delta (a)$,
		$$\phi _a^1 = \frac{\alpha }{1-\alpha } (\phi _0^c + (1 - \phi _0^c)\phi ), \phi _N = 2\alpha (\phi _0^c + (1 - \phi _0^c)\phi )$$,
		$$P(G=11|Z=z, G_0 =1)= 1 - \phi $$,
		$$P(G=12|Z=z, G_0 =1)= \phi $$,
		$$P(G=21|Z=z, G_0 =2)= 1$$,
		$$ P(G=22|Z=z,G_0=2)= 0$$
Targeted ITN	ITN if WAZ $< \theta$ and unprotected	$p_I = p, \phi _t^{(1)}=P(Z<\theta ), \phi _t^{(2)} = 0$, $\phi _0^c$ fixed, $f_A(a)=\delta (a)$,
		$$\phi _a^1 = \frac{\alpha }{1-\alpha } (\phi _0^c + (1 - \phi _0^c)\phi _t^{(1)}), \phi _N = 2\alpha (\phi _0^c + (1 - \phi _0^c)\phi _t^{(1)})$$,
		$$P(G=11|Z=z, G_0 =1)= 1_{\{z>\theta \}}$$,
		$$P(G=12|Z=z, G_0 =1)= 1_{\{z<\theta \}}$$,
		$$P(G=21|Z=z, G_0 =2)= 1$$,
		$$ P(G=22|Z=z,G_0=2)= 0$$
Targeted food and	food if WAZ $< \theta$,	$p_I = p, \phi _t^{(1)}= \phi _t^{(2)} = P(Z<\theta )$, $\phi _0^c$ fixed,
Targeted ITN	ITN if WAZ $< \theta$ and unprotected	$\phi _a^1 = \frac{\alpha }{1-\alpha } (\phi _0^c + (1 - \phi _0^c)\phi _t^{(1)}),\, \phi _N = 2\alpha (\phi _0^c + (1 - \phi _0^c)\phi _t^{(1)})$,
		$$P(G=11|Z=z, G_0 =1)= 1_{\{z>\theta \}}$$,
		$$P(G=12|Z=z, G_0 =1)= 1_{\{z<\theta \}}$$,
		$$P(G=21|Z=z, G_0 =2)= 1_{\{z>\theta \}}$$,
		$$ P(G=22|Z=z,G_0=2)=1_{\{z<\theta \}}$$

The last column specifies the restrictions on the parameter values in Eqs. (4)–(6), where $1_{\{x\}}$ is the indicator function of the event *x*, and $\delta (a)$ is the Dirac delta function

### The full model

The general model that covers all four intervention policies divides children into four groups: group 11 does not have ITN protection at baseline and does not receive any interventions (food or ITNs), group 12 does not have ITN protection at baseline and receives intervention, group 21 has ITN protection at baseline and does not receive additional intervention, group 22 has ITN protection at baseline and receives additional intervention (food in the case of the targeted food and the targeted food and ITN policies). Let the groups be indexed by $G=11,\, 12,\, 21$ and 22, and let *I* denote the event that a random person is infected. Let $x_{ij}(s)=P(I|S=s,G=ij)$ be the proportion of the population in each group that is infected, and let $f_S^{(ij)}(s)$ be the PDF of the susceptibility of the group $G=ij$. Let $G_0 = 1$ index the sub-population that does not have ITNs at baseline and $G_0 = 2$ index the group that has ITNs. Defining $\phi _t^{(1)}, \phi _t^{(2)}$ to be the policy-dependent proportion of the population in groups $G_0 = 1, G_0 =2$ that receives food or ITNs and introducing the policy-dependent probabilities $P(G=ij|Z=z, G_0 = i)$ that are specified in Table 2, it follows that

12 $$\begin{aligned} f_S^{(11)}(s)&= \frac{1}{1-\phi _t^{(1)}}\int _{-\infty }^{\infty }f_V(s-u(z))f_Z(z)\\ &\qquad\qquad \qquad P(G=11|Z=z, G_0 = 1)~dz,\end{aligned}$$

13 $$\begin{aligned} f_S^{(12)}(s)&= \frac{1}{\phi _t^{(1)}}\int _{-\infty }^{\infty }f_V(s-u(z+a))\int _0^{\infty }f_Z(z)f_A(a)P(G=12|Z=z, G_0 = 1)~da~dz,\end{aligned}$$

14 $$\begin{aligned} f_S^{(21)}(s)&= \frac{1}{1-\phi _t^{(2)}}\int _{-\infty }^{\infty }f_V(s-u(z))f_Z(z)P(G=21|Z=z, G_0 = 2)~dz, \end{aligned}$$

15 $$\begin{aligned} f_S^{(22)}(s)&= \frac{1}{\phi _t^{(2)}}\int _{-\infty }^{\infty }f_V(s-u(z+a))\int _0^{\infty }f_Z(z)f_A(a)P(G=22|Z=z, G_0 = 2)~da~dz. \end{aligned}$$

Let $\phi _a^1$ be the proportion of adults protected by ITNs post-intervention, assuming one adult is protected for every protected child. Let $\phi _N$ denote the proportion of the entire population that is protected by ITNs, and let $p_I$ be the probability that a mosquito successfully bites a human under a net in the case of interventions involving ITNs, and be equal to one otherwise. The full model is

16 $$\begin{aligned} \dot{x}_{11}(s)&= bsa(\phi _N)m(\phi _N)y(1-x_{11}(s)) - g(bsa(\phi _N)m(\phi _N)y,r)x_{11}(s),\end{aligned}$$

17 $$\begin{aligned} \dot{x}_{12}(s)&= bsp_Ia(\phi _N)m(\phi _N)y(1-x_{12}(s)) - g(bsp_Ia(\phi _N)m(\phi _N)y,r)x_{12}(s),\end{aligned}$$

18 $$\begin{aligned} \dot{x}_{21}(s)&= bspa(\phi _N)m(\phi _N)y(1-x_{21}(s)) - g(bspa(\phi _N)m(\phi _N)y,r)x_{21}(s),\end{aligned}$$

19 $$\begin{aligned} \dot{x}_{22}(s)&= bspa(\phi _N)m(\phi _N)y(1-x_{21}(s)) - g(bspa(\phi _N)m(\phi _N)y,r)x_{21}(s),\end{aligned}$$

20 $$\begin{aligned} \dot{w}_1&= ba(\phi _N)m(\phi _N)y(1-w_1) - g(ba(\phi _N)m(\phi _N)y,r)w_1,\end{aligned}$$

21 $$\begin{aligned} \dot{w}_2&= ba(\phi _N)m(\phi _N)py(1-w_2) - g(ba(\phi _N)m(\phi _N)py,r)w_2,\end{aligned}$$

22 $$\begin{aligned} \dot{y}&= a(\phi _N)ch(1-y)-\mu (\phi _N)y, \end{aligned}$$

where

23 $$\begin{aligned} h &{}= \alpha \Biggl ( \left( 1- \phi _0^c\right) \Bigl ( \left( 1 - \phi _t^{(1)}\right) \int _0^{\infty }x_{11}(s)f_S^{(11)}(s)~ds + p_I \phi _t^{(1)} \int _0^{\infty }x_{12}(s)f_S^{(12)}(s)~ds \Bigr ) \\ &{}\quad + p\phi _0^c \Bigl ( (1 - \phi _t^{(2)}) \int _0^{\infty }x_{21}(s)f_S^{(21)}(s)~ds + \phi _t^{(2)} \int _0^{\infty } x_{22}(s)f_S^{(22)}(s)~ds \Bigr ) \Biggr ) \\ &{}\quad + (1-\alpha ) \Bigl (\left( 1- \phi _1^a\right) w_1 + p\phi _1^a w_2 \Bigr ). \end{aligned}$$

To specify the model for each policy, the parameter values given in Table 1 are imposed in (16)–(23).

### Performance measures

Two performance measures are associated with model (16)–(23): the clinical malaria prevalence $P_c$ (i.e., the proportion of children who have clinical malaria) and the malaria mortality *D* (i.e., the proportion of children who die from malaria). The analytical derivations of $P_c$ and *D* for the five cases in Table 2 appear in Additional file 1: §1, and are briefly outlined here. The equilibrium solution to (16)–(23) is given by

24 $$\begin{aligned} \bar{x}_{11}(s)&= 1- e^{-bsa(\phi _N)m(\phi _N)\bar{y}/r},\end{aligned}$$

25 $$\begin{aligned} \bar{x}_{12}(s)&= 1- e^{-bsp_Ia(\phi _N)m(\phi _N)\bar{y}/r},\end{aligned}$$

26 $$\begin{aligned} \bar{x}_{21}(s)&= 1- e^{-bspa(\phi _N)m(\phi _N)\bar{y}/r},\end{aligned}$$

27 $$\begin{aligned} \bar{x}_{22}(s)&= 1- e^{-bspa(\phi _N)m(\phi _N)\bar{y}/r},\end{aligned}$$

28 $$\begin{aligned} \bar{w}_1&= 1- e^{-ba(\phi _N)m(\phi _N)\bar{y}/r},\end{aligned}$$

29 $$\begin{aligned} \bar{w}_2&= 1- e^{-bpa(\phi _N)m(\phi _N)\bar{y}/r},\end{aligned}$$

30 $$\begin{aligned} \bar{y}&= \frac{a(\phi _N)c \bar{h}}{a(\phi _N)c \bar{h} + \mu (\phi _N)}, \end{aligned}$$

where

31 $$\begin{aligned} \bar{h} &{}= \alpha \Biggl (\left( 1- \phi _0^c\right) \left( \left( 1 - \phi _t^{(1)}\right) \int _0^{\infty }\bar{x}_{11}(s)f_S^{(11)}(s)~ds + p_I \phi _t^{(1)} \int _0^{\infty }\bar{x}_{12}(s)f_S^{(12)}(s)~ds \right) \\ &{} \quad + p\phi _0^c \Bigl ( \left( 1 - \phi _t^{(2)}\right) \int _0^{\infty } \bar{x}_{21}(s)f_S^{(21)}(s)~ds + \phi _t^{(2)} \int _0^{\infty } \bar{x}_{22}(s)f_S^{(22)}(s)~ds \Bigr )\Biggr ) \\ &{} \quad + (1-\alpha ) \Bigl (\left( 1- \phi _1^a\right) \bar{w}_1 + p\phi _1^a \bar{w}_2 \Bigr ). \end{aligned}$$

The pair ($\bar{y}, \bar{h})$ are obtained by jointly solving the fixed point Eqs. (30)–(31). The prevalence of malaria infection in children is then given by

32 $$\begin{aligned} \begin{array}{ll} P &{}= ( 1- \phi _0^c) \left( \left( 1 - \phi _t^{(1)}\right) \int _0^{\infty }\bar{x}_{11}(s)f_S^{(11)}(s)~ds{ + }\phi _t^{(1)} \int _0^{\infty }\bar{x}_{12}(s)f_S^{(12)}(s)~ds \right) \\ &{} \quad{ + }\phi _0^c \left( \left( 1 - \phi _t^{(2)}\right) \int _0^{\infty } \bar{x}_{21}(s)f_S^{(21)}(s)~ds{ + }\phi _t^{(2)} \int _0^{\infty } \bar{x}_{22}(s)f_S^{(22)}(s)~ds \right) . \end{array} \end{aligned}$$

Let $p_c(\mathcal{E})$ denote the probability of a child under 5 years of age developing clinical disease given malaria infection. The model in [28] is used to obtain this probability as a function of the EIR $\mathcal{E}$ (which equals $a(\phi _N) m(\phi _N) \bar{y}$) in Additional file 1: §2.4. The prevalence of clinical malaria in children is then given by

33 $$\begin{aligned} P_c = p_c(\mathcal{E})P. \end{aligned}$$

Let $Z_1$ represent the post-intervention WAZ score. Let $p_{ij}(z) = P(I|Z_1=z, G=ij)$ and $f_{Z_1|G = ij}(z)$ be the PDF of $Z_1|G = ij$ for $i,j \in {1,2}$, which are derived in §1 of Additional file 1 for the five cases in Table 2. The malaria mortality in each of the groups is given by the product of four probabilities: the PDF of post-intervention WAZ ($f_{Z_1|G=ij}(z)$), the probability of being infected with malaria if WAZ $=z$ (i.e., $p_{ij}(z)$), the probability of showing clinical symptoms given infection ($p_c(\mathcal{E})$), and the probability of death from malaria conditioned on having clinical disease and having WAZ $=z$, which is denoted by *d*(*z*). The malaria mortality is then given by

34 $$\begin{aligned} \begin{array}{ll} D &{}= \left( 1- \phi _0^c\right) \left( \left( 1 - \phi _t^{(1)}\right) \int _{-\infty }^{\infty } d(z)p_c(\mathcal{E})p_{11}(z) f_{Z_1|G=11}(z)~dz + \phi _t^{(1)} \int _{-\infty }^{\infty } d(z)p_c(\mathcal{E})p_{12}(z) f_{Z_1|G=12}(z)~dz \right) \\ & \quad{ + }\phi _0^c \left( \left( 1 - \phi _t^{(2)}\right) \int _{-\infty }^{\infty } d(z)p_c(\mathcal{E})p_{21}(z) f_{Z_1|G=21}(z)~dz{ + }\phi _t^{(2)} \int _{-\infty }^{\infty } d(z)p_c(\mathcal{E})p_{22}(z) f_{Z_1|G=22}(z)~dz \right) . \end{array} \end{aligned}$$

### Parameter estimates

An update (Table A7.3 in [21]) of the analysis in [18] gives $k=1/5.9=0.17$ and $b/r=0.57$ (the results depend on *b* and *r* only through their ratio). Assume $c=0.5$ (Table 4 in [23]) and $p=0.1$ [20]. The sub-Saharan Africa CDF in Fig. 2b of [24] was digitized and fit to a normal distribution to obtain $\mu _Z=-1.00$ and $\sigma _Z=1.27$. Data from the Population Reference Bureau for the year 2008 [22] yield $\alpha = 0.17$. Data from the only large randomized controlled food (500 kCal/day of ready-to-use therapeutic food for 3 months) trial with a treatment-free control group [19] are used to estimate the increase in WAZ due to supplementary food. This trial reported only height-for-age z scores (HAZ) and weight-for-height z scores (WHZ), and by considering a typical child that was of mean age, baseline HAZ and baseline WHZ, and achieved the mean increases in HAZ and WHZ from supplementary food, the mean increase in WAZ is roughly estimated to be 0.23 (§2.1 of Additional file 1). For lack of data, the standard deviation of the WAZ increase is assumed to be 0.1, which yields the lognormal parameters $\mu _A=-1.56$ and $\sigma _A=0.42$.

**Fig. 1:**
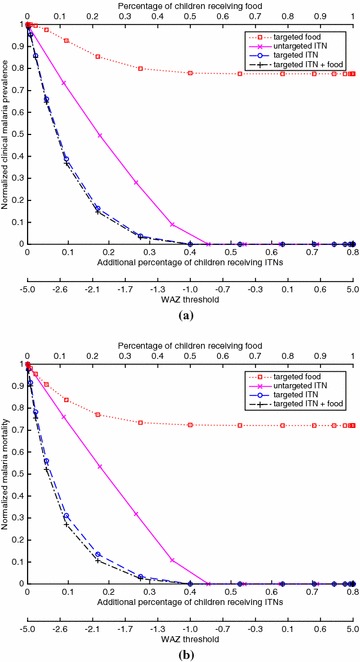
For the case of EIR = 1 (hypoendemic) and 20 % baseline ITN coverage of children, which generates a clinical malaria prevalence of 0.049 in children at baseline, **(a)** the proportion of children with clinical malaria for a given coverage of a given policy, divided by the proportion of children with clinical malaria in the intervention-free case, and **(b)** the proportion of children who die from malaria for a given coverage of a given policy, divided by the proportion of children who die from malaria in the intervention-free case. The *bottom* of both figures gives the WAZ threshold that corresponds to a given coverage for the targeted policies (e.g., for the targeted ITN policy, ITNs are given to children with WAZ values below the threshold)

**Fig. 2:**
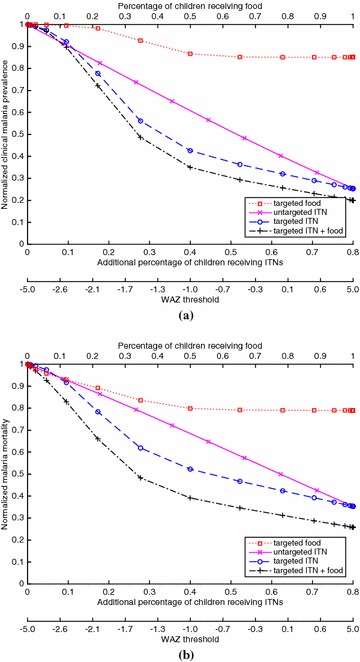
For the case of EIR = 10 (mesoendemic) and 20 % baseline ITN coverage of children, which generates a clinical malaria prevalence of 0.354 in children at baseline, **(a)** the proportion of children with clinical malaria for a given coverage of a given policy, divided by the proportion of children with clinical malaria in the intervention-free case, and **(b)** the proportion of children who die from malaria for a given coverage of a given policy, divided by the proportion of children who die from malaria in the intervention-free case. The *bottom* of both figures gives the WAZ threshold that corresponds to a given coverage for the targeted policies (e.g., for the targeted ITN policy, ITNs are given to children with WAZ values below the threshold)

**Fig. 3:**
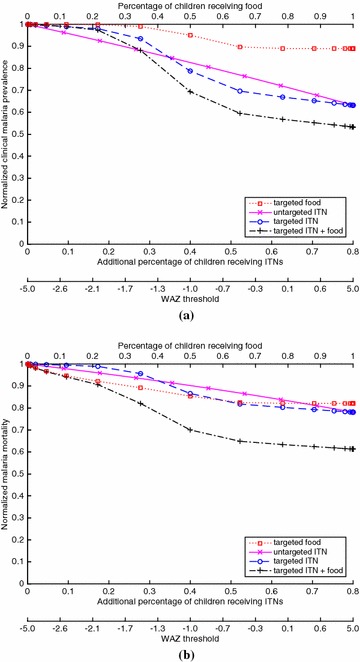
For the case of EIR = 100 (hyperendemic) and 20 % baseline ITN coverage of children, which generates a clinical malaria prevalence of 0.580 in children at baseline, **(a)** the proportion of children with clinical malaria for a given coverage of a given policy, divided by the proportion of children with clinical malaria in the intervention-free case, and **(b)** the proportion of children who die from malaria for a given coverage of a given policy, divided by the proportion of children who die from malaria in the intervention-free case. The *bottom* of both figures gives the WAZ threshold that corresponds to a given coverage for the targeted policies (e.g., for the targeted ITN policy, ITNs are given to children with WAZ values below the threshold)

The functions $a(\phi _N)$, $m(\phi _N)$ and $\mu (\phi _N)$ are taken from the feeding cycle model in [20] and are specified in Eqs. (58)–(60) in Additional file 1 and plotted in Additional file 1: Figures 1–3. Because the EIR varies greatly in different regions [18], four versions of $m(\phi )$ are considered, which correspond to pre-intervention EIR values of 1, 10, 100 and 500 in the absence of baseline coverage (i.e., $\phi _0^c=\phi _0^a=0)$. These versions generate examples of hypoendemic, mesoendemic and (for EIR = 100 and 500) hyperendemic regions, respectively (Table 1 in [29]), and are constructed by changing the value of the parameter $\lambda$ in Eq. (82) of Additional file 1, as explained in Additional file 1: §2.2. The clinical fraction $p_c(\mathcal{E})$ is estimated using the anti-disease immunity model in [28] (Additional file 1: §2.4).

**Fig. 4:**
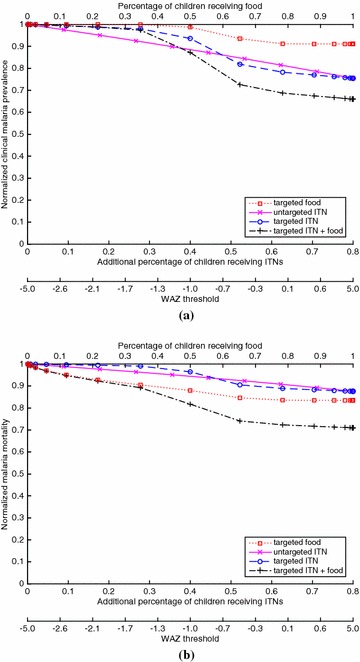
For the case of EIR = 500 (hyperendemic) and 20 % baseline ITN coverage of children, which generates a clinical malaria prevalence of 0.680 in children at baseline, **(a)** the proportion of children with clinical malaria for a given coverage of a given policy, divided by the proportion of children with clinical malaria in the intervention-free case, and **(b)** the proportion of children who die from malaria for a given coverage of a given policy, divided by the proportion of children who die from malaria in the intervention-free case. The *bottom* of both figures gives the WAZ threshold that corresponds to a given coverage for the targeted policies (e.g., for the targeted ITN policy, ITNs are given to children with WAZ values below the threshold)

A critical and challenging parameter to estimate is $k_1$, which quantifies the proportion of heterogeneity in susceptibility that is due to undernutrition. To measure the change in malaria prevalence due to providing supplementary food, the impact of undernutrition on malaria needs to be isolated from other confounding factors that may be positively correlated with undernutrition, such as family income, home location relative to mosquito-breeding areas, and the protective ability of the home (e.g., window screens). Consequently, although there are estimates that quantify the relative risk of WAZ $<-2$ for clinical malaria [13], if these estimates were used in the model, the impact of food on malaria would be overestimated by implicitly assuming that providing food would also improve the level of the confounding variables. Hence, to estimate $k_1$, data are used from the only large randomized controlled food trial of children ages 6 months to 5 years with a treatment-free control group [19], which found that the adjusted (after accounting for age, sex, seasonality, HAZ, and village) odds ratio for post-treatment clinical malaria (requiring a fever and an infection as measured by the HRP2 rapid diagnostic test, which is very sensitive and specific [30]) was 0.76. This analysis (Additional file 1: §2.3) yields $k_1=0.153$ (Additional file 1: Figure 4), which corresponds to 90.3 % of the heterogeneity in susceptibility being due to undernutrition (i.e., using the mean values, $k_1/k=0.903$).

The malaria mortality probability for the infected population, *d*(*z*), is equal to the unconditional malaria mortality probability divided by the probability of having clinical malaria, where all three probabilities are conditioned on having a WAZ value of *z*. In the derivation of *d*(*z*) (Additional file 1: §2.5), the numerator is estimated from Table 2.5 of [13], and the denominator is calculated from the estimated relative risk of clinical malaria (this estimate relies heavily on [25], which required fever and infection via microscopy) of 1.31 for WAZ $< -2$ (Table 2.8 of [13]) and an estimate of the underling WAZ PDF for the population upon which this relative risk is based [25]. This calculation yields $d(z)=e^{1.851-0.607z}$ per 1000 children (Additional file 1: Figure 7).

## Results

### Main results

Results are reported for all 12 combinations of 20, 50 and 80 % baseline ITN coverage and pre-intervention (and no baseline coverage) EIR of 1, 10, 100 and 500, except for the 80 % ITN coverage, EIR = 1 scenario, which achieves malaria elimination in the absence of intervention. To place these EIR values in context, the setting of [19] has a pre-intervention (and no baseline coverage) EIR value of 2.65. For these 11 scenarios, two quantities are computed for each policy: the normalized clinical malaria prevalence (i.e., clinical malaria prevalence divided by intervention-free clinical malaria prevalence, where the latter number is shown in the figure legends), and the normalized malaria mortality (i.e., malaria mortality divided by the intervention-free malaria mortality). Some representative results from these scenarios appear in Table 3.

**Table 3:** Selected numerical results

Baseline ITN coverage (%)	Metric	EIR = 1	EIR = 10	EIR =100	EIR = 500
20	$1-D_\mathrm{TF}$	0.23	0.11	0.08	0.07
20	$D_\mathrm{UI}-D_\mathrm{TI}$	0.40	0.08	−0.03	−0.01
20	$D_\mathrm{TI}-D_\mathrm{TF+I}$	0.03	0.12	0.08	0.07
50	$1-D_\mathrm{TF}$	0.35	0.14	0.09	0.07
50	$D_\mathrm{UI}-D_\mathrm{TI}$	NA	0.08	−0.02	−0.01
50	$D_\mathrm{TI}-D_\mathrm{TF+I}$	NA	0.14	0.09	0.07
80	$1-D_\mathrm{TF}$	NA	0.19	0.10	0.08
80	$D_\mathrm{UI}-D_\mathrm{TI}$	NA	0.06	−0.01	−0.01
80	$D_\mathrm{TI}-D_\mathrm{TF+I}$	NA	0.18	0.11	0.08

The quantity $D_\mathrm{P}$ is the normalized malaria mortality achieved by policy P

The subscripts TF, UI, TI and TF+I stand for the targeted food policy, the untargeted ITN policy, the targeted ITN policy and the targeted food and targeted ITN policy, respectively, where the WAZ threshold $\theta =-2$ for the targeted policies and the additional ITN coverage $\phi$ equals the proportion of children with WAZ $<-2$ without ITN coverage at baseline

Hence, $1-D_\mathrm{TF}$ is the reduction in normalized malaria mortality from the targeted food policy, $D_\mathrm{UI}-D_\mathrm{TI}$ is the reduction in normalized malaria mortality due to targeting ITNs (and is negative if targeting is worse than not targeting), and $D_\mathrm{TI}-D_\mathrm{TF+I}$ is the marginal reduction in normalized malaria mortality due to adding targeted food to the targeted ITN policy

NA represents the case where both policies in the metric column eliminate malaria

All numbers are taken from Figs. 1b, 2b, 3b, 4b, 5b, 6b and 7b and from Additional file 1: Figures 9b, 10b, 11b, 12b

Figures 1, 2, 3 and 4 show results for 20 % baseline coverage for children $(\phi _c=0.2$) for pre-intervention EIR = 1, 10, 100 and 500, respectively. When EIR = 1 and ITN coverage = 20 %, the targeted food policy achieves a normalized clinical malaria prevalence of 0.77 when $\theta = \infty$ (i.e., every child receives food). This policy experiences decreasing returns as the threshold $\theta$ increases, with the elbow of the curve in Fig. 1a near WAZ = −1.5. The reductions in normalized malaria mortality are larger than the corresponding reductions in normalized malaria prevalence for the targeted food policy, regardless of EIR and pre-intervention coverage; i.e., the targeted food policy curves in Figs. 1b, 2b, 3b and 4b are lower than the corresponding curves in Figs. 1a, 2a, 3a and 4a. In particular, in Fig. 1b the reduction in malaria mortality is 0.72 when $\theta =\infty$ for the targeted food policy.

The untargeted ITN curve is nearly linear in Fig. 1 (and in all other figures), while the curves for the three targeted policies are all convex. The untargeted ITN policy achieves more dramatic morbidity and mortality reductions than the targeted food policy, and is capable of eliminating malaria in this low-EIR setting when ${\approx}45\,\%$ children, in addition to the 20 % children at baseline, are randomly chosen to receive an ITN. The targeted ITN policy is even more effective, eliminating malaria when an additional ${\approx}40\,\%$ of the children—those with WAZ $<-1.0$—receive ITNs. At 30 % coverage of the child population (including the 20 % at baseline), the targeted ITN policy achieves a 69 % reduction in mortality and the untargeted ITN policy achieves a 24 % reduction. The policy that targets both food and ITNs achieves morbidity and mortality levels that are almost indistinguishable in Fig. 1 from those achieved by the targeted ITN policy.

As expected (both empirically [6] and because malaria prevalence is an increasing concave function of EIR in the model), the interventions have less impact on malaria morbidity and mortality as EIR increases; e.g., for 20 % baseline coverage, the curves in Fig. 1 (EIR = 1) are lower than the corresponding curves in Fig. 2 (EIR = 10), which are lower than the corresponding curves in Fig. 3 (EIR = 100), which in turn are lower than the corresponding curves in Fig. 4 (EIR = 500). The clinical malaria prevalence curves for the targeted food policy asymptote (for $\theta =\infty$) at 0.85, 0.89 and 0.91 in Figs. 2a, 3a and 4a respectively, while the mortality curves asymptote at 0.80, 0.82 and 0.83 in Figs. 2b, 3b and 4b respectively. In contrast to the EIR = 1 scenario, malaria elimination is not achievable when EIR = 10, 100 or 500, even with 100 % ITN coverage of the child population (which corresponds to 34 % of the entire population).

For the targeted food policy in Figs. 2a, 3a and 4a, the normalized prevalence equals 1.0 for very low coverage because undernutritioned children (e.g., WAZ $<-2$ in Fig. 3a) get infected even if they receive food. Similarly, the targeted ITN policy performs worse than the untargeted ITN policy for low coverage in the hyperendemic setting (Figs. 3, 4) because children with low WAZ are highly likely to get infected regardless of the intervention policy. In addition, adding targeted food to targeted ITNs has the biggest impact when EIR is intermediate in value (i.e., EIR = 10 or 100).

**Fig. 5:**
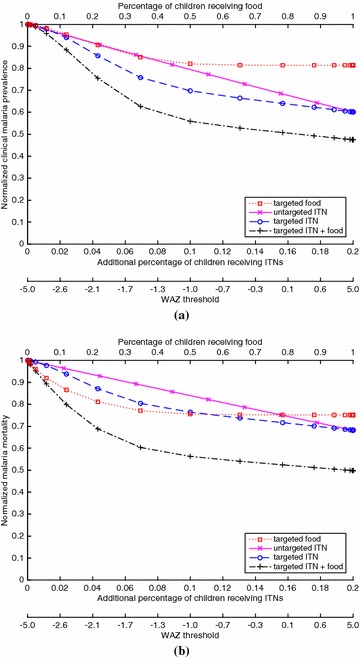
For the case of EIR = 10 (mesoendemic) and 80 % baseline ITN coverage of children, which generates a clinical malaria prevalence of 0.149 in children at baseline, **(a)** the proportion of children with clinical malaria for a given coverage of a given policy, divided by the proportion of children with clinical malaria in the intervention-free case, and **(b)** the proportion of children who die from malaria for a given coverage of a given policy, divided by the proportion of children who die from malaria in the intervention-free case. The *bottom* of both figures gives the WAZ threshold that corresponds to a given coverage for the targeted policies (e.g., for the targeted ITN policy, ITNs are given to children with WAZ values below the threshold)

**Fig. 6:**
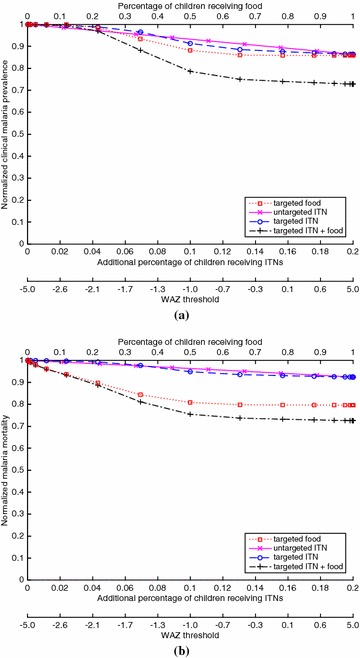
For the case of EIR = 100 (hyperendemic) and 80 % baseline ITN coverage of children, which generates a clinical malaria prevalence of 0.424 in children at baseline, **(a)** the proportion of children with clinical malaria for a given coverage of a given policy, divided by the proportion of children with clinical malaria in the intervention-free case, and **(b)** the proportion of children who die from malaria for a given coverage of a given policy, divided by the proportion of children who die from malaria in the intervention-free case. The *bottom* of both figures gives the WAZ threshold that corresponds to a given coverage for the targeted policies (e.g., for the targeted ITN policy, ITNs are given to children with WAZ values below the threshold)

**Fig. 7:**
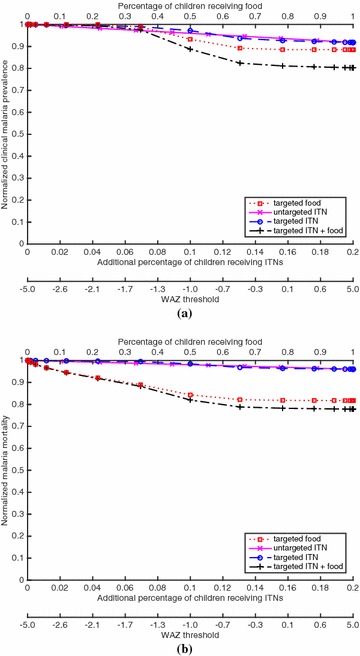
For the case of EIR = 500 (hyperendemic) and 80 % baseline ITN coverage of children, which generates a clinical malaria prevalence of 0.558 in children at baseline, **(a)** the proportion of children with clinical malaria for a given coverage of a given policy, divided by the proportion of children with clinical malaria in the intervention-free case, and **(b)** the proportion of children who die from malaria for a given coverage of a given policy, divided by the proportion of children who die from malaria in the intervention-free case. The *bottom* of both figures gives the WAZ threshold that corresponds to a given coverage for the targeted policies (e.g., for the targeted ITN policy, ITNs are given to children with WAZ values below the threshold)

The four scenarios with 50 % baseline coverage are qualitatively similar to Figs. 1, 2, 3 and 4 except that the horizontal axis ranges from 0 to 0.5 rather than from 0 to 0.8; the results for 50 % ITN coverage appear in Additional file 1: Figures 9–12. Finally, under 80 % baseline coverage (results for the EIR = 10, 100 and 500 scenarios are in Figs. 5, 6 and 7), ITN interventions have very limited impact when EIR = 100 and 500, and targeted food offers larger morbidity and mortality reductions than additional ITN interventions.

### Sensitivity analysis

Because $k_1$ is the most critical and problematic parameter in the model, a sensitivity analysis is undertaken using values of $k_1=0.08$ and 0.04, which generate $k_1/k$ values (the portion of susceptibility heterogeneity due to undernutrition) of 0.471 and 0.235, respectively, compared to the base-case value of $k_1/k=0.903$. As expected (Additional file 1: Figures 13–34), as $k_1$ decreases, the impact of the targeted policies decreases. Nonetheless, even at $k_1=0.04$, the qualitative results described earlier still hold, particularly for malaria mortality.

## Discussion

### Intervention results

Eleven scenarios are considered that span a range of baseline ITN coverage values and EIR values. Although spatial targeting should cause these two quantities to be positively correlated in practice, the large funding gap in ITNs [10] suggests that the only coverage-EIR combination considered here that may be rare in practice is the 80 % coverage, EIR = 10 scenario.

The first of three main results from the model is that the targeted food policy achieves a nontrivial reduction in malaria mortality; e.g., under 20 % baseline ITN coverage, providing supplementary food to underweight children (i.e., those with WAZ $<-2$) reduces malaria mortality by 22.9, 10.8, 7.8 and 7.2 % when EIR = 1, 10, 100 and 500, respectively. The morbidity reduction in the model explicitly incorporates the herd effect of nutrition: children who receive supplementary food become less susceptibile to disease, which leads to fewer infected mosquitoes, which in turn reduces the likelihood of disease in children who do not receive supplementary food. The mortality reduction is larger than the morbidity reduction because the children who receive food are also the most likely to die if they do get infected with malaria, and so their direct protection via supplementary food has a synergistic effect on the overall mortality due to malaria. As with ITNs, the impact of the targeted food policy is lower in higher EIR settings.

As expected, the untargeted ITN policy has a much larger effect than the targeted food policy on clinical malaria prevalence, although this effect decreases with increasing EIR, which is consistent with results from randomized controlled trials [6]. The model predicts malaria elimination when EIR = 1 with 60 % ITN coverage of the child population, which corresponds to 20 % of the total population. It also predicts that 100 % ITN coverage of children, which corresponds to 34 % coverage of the entire population, is insufficient to eliminate malaria when EIR $\ge 10$. The model predictions about the infeasibility of malaria elimination in many scenarios is not inconsistent with results from randomized controlled trials [6] or other modelling studies (Table 1 in [31]). A cost-effectiveness comparison between supplementary food and ITNs has not been performed because supplementary food may also directly reduce mortality from wasting and stunting and reduce the lifelong effects of stunting, in addition to reducing morbidity and mortality associated with other diseases such as pneumonia and diarrhoea.

The second main result is that in the hypoendemic and mesoendemic settings, the targeted ITN policy outperforms the untargeted ITN policy (it achieves elimination at a lower coverage, and significantly reduces mortality over a wide range of sub-elimination coverages). However, the targeted ITN policy is outperformed by the untargeted ITN policy for conventional WAZ thresholds (e.g., WAZ $\in [-3, -1]$) in the hyperendemic setting because undernutritioned children in this case are likely to get infected despite being protected by an ITN. While ITN targeting is typically performed at the macro level based on spatial estimates of transmission intensity [21], these results raise the possibility of additional targeting at the micro level in the hypoendemic and mesoendemic settings based on easily-obtained anthropometric measures such as WAZ, despite the fact that young children are not a major contributor to the infectious reservoir [28]. In addition, targeting based on child undernutrition may be more politically feasible and practically implementable than means-testing, where family incomes are the basis for ITN distribution [32]. This targeting approach is particularly appealing for sub-Saharan Africa, which incurs 90 % of malarial cases and deaths, and where the burden of disease is in young children (and pregnant women) [33].

The policy that targets ITNs and food performs nearly the same as the targeted ITN policy when EIR = 1. This lack of improvement may be due to the positive correlation of the two interventions (i.e., they are targeting the exact same children); efficacy of a joint strategy is often improved if two interventions are negatively correlated, in that they generate higher coverage [28]. However, the improvements from adding targeted food to targeted ITNs are sizeable in the mesoendemic and hyperendemic settings. The third main result is that in a hyperendemic setting with 80 % ITN child coverage, food targeting offers a larger reduction in malaria morbidity and mortality than increasing the child ITN coverage beyond 80 %, which is often a logistical challenge.

Overall, the study suggests that much of the heterogeneity in susceptibility is observable (in this case, via WAZ values) and hence exploitable for purposes of targeting, which is sufficient to generate meaningful reductions in clinical malaria prevalence in some settings. Coupling this effect with the dependence of mortality on WAZ leads to even larger reductions when considering malaria mortality.

Although beyond the scope of this study, a similar analysis—but with a Susceptible-Exposed-Infected-Removed (SEIR) model with heterogeneous susceptibility rather than a vector model as in (2)–(5)—could be performed for the cases of diarrhoea (e.g., rotavirus) or pneumonia (e.g., respiratory syncytial virus), using either partial differential equations [34] or branching processes [1]. Such an analysis could quantify the benefits of other targeted preventive measures—e.g., rotavirus vaccination—to undernutritioned children. The relative risks for morbidity associated with WAZ $<-2$ are 1.23 and 1.86, respectively, for pneumonia and diarrhoea [13], and mortality rates for these two diseases decrease with increasing WAZ (Table 2.5 in [13]).

A possible generalization of the model is to incorporate the possibility that children’s nutrition level (e.g., WAZ) decreases when they are infected [14–16]. Capturing this effect and the subsequent catch-up growth (pages 182–183 of [35]) would require generalizing Eqs. (4)–(6) to a partial differential equation model, where $\dot{x}_i(s)$ is replaced by $\frac{\partial x_i(s,t)}{\partial t}$ for $i=1,2$. This generalization, which would be more difficult to analyze, may not yield any new qualitative results because of the catch-up growth.

### Limitations of the study

The integrated nutrition-malaria model presented here simplifies aspects of nutrition and malaria. Undernutrition in this model is measured by WAZ (i.e., underweight), which can be viewed as a composite measure of HAZ (i.e., stunting), which is a long-term micronutrient deficiency that is caused by insufficiently balanced diets as well as repeated infection and psycho-social deprivation, and WHZ (i.e., wasting), which is an acute undersupply in energy and proteins (an alternative view is that wasting is a composite measure of stunting and underweight). Combining these into a single measure tends to muddle the interaction of nutrition with malaria. However, the best malaria mortality data [13] explores its relationship only with WAZ and prevents us from developing a bivariate model using (HAZ,WHZ). If better data become available, a bivariate model may lead to a refinement of these findings, although the model would be considerably more difficult to analyse.

The malaria model ignores many complexities that have been incorporated in other malaria models, such as seasonality, spatial structure, age structure, immunity to infection (although this aspect did not improve the model fit in [18]), and mosquito searching and feeding cycle (e.g., [20, 28, 36]), and temporal issues related to the relative effectiveness of ITNs and the new generation of long-lasting insecticide-treated nets (LLINs). Nonetheless, given the research questions being raised (i.e., attempting to gain broad insights about targeted interventions as opposed to accurately predicting morbidity and mortality rates), these omissions seem appropriate, and the ITN parameters are estimated from the output of the more detailed model in [20]. On the other hand, the model is more detailed (although much less broad) than the Lives Saved Tool [37], which—while invaluable for broad resource allocation decisions for maternal and child health—is not able to address the type of targeting questions and policies considered here.

Despite these modelling limitations, the biggest shortcoming in this analysis relates to the estimation of the crucial parameter, $k_1$, which specifies the proportion of susceptibility heterogeneity that is due to undernutrition. First, the estimation of the total susceptibility heterogeneity (i.e., the parameter *k* in the model and in [18, 21]) is extremely difficult [38]. Several modelling choices need to be made without supporting data. In [18, 21], it was assumed that the susceptibility distribution had a gamma distribution. A much bolder assumption is made here that the undernutrition random variable is also gamma with the same shape parameter as the susceptibility distribution derived in [21], so that only one new parameter ($k_1$) needs to be estimated. It is further assumed that the left tail of the WAZ distribution corresponds to the right tail of the undernutrition distribution. In addition, the analysis in [18] considers children up to 15 years of age, and their results are applied here to children up to 5 years of age. Turning to the data used to estimate $k_1$, the adjusted odds ratio of 0.76 (which gives an adjusted prevalence ratio of 0.77) in [19] has a 95 % confidence interval (CI) of (0.51,1.13), and a p value of 0.177, and hence is not statistically significant at the traditional 0.05 level.

Interestingly, a more recent randomized controlled feeding trial [39] of 54 g/day (slightly less than half the dose used in [19]) of a lipid-based nutrient supplement had very similar results to [19]: pooling the three intervention arms (milk-, soy- and corn-soy-based) and comparing to the control arm leads to an incident rate ratio of clinical malaria (fever and infection determined via microscopy) of 0.81 and a 95 % CI of (0.69, 0.94), which is statistically significant (in [39], the three intervention arms were not pooled and did not achieve statistical significance). The results in [39] cannot be directly pooled with those in [19] because of the lower food dose, the restricted ages (6–18 months old), the higher pre-intervention nutrition levels (mean WAZ = $-$0.8, mean WHZ = 0.4), and the higher malaria infection prevalence (0.13). Nonetheless, the consistency in results between these two studies suggests that this result may be robust.

If the relative risk of clinical malaria of 1.31 associated with WAZ $<-2$ [13]—rather than the feeding trial data in [19]—is used to estimate $k_1$, then $k_1=0.0095$ (Additional file 1: §2.5), which corresponds to 5.3 % of heterogeneity being due to undernutrition and which generates a negligible impact of food on malaria morbidity, although it still reduces malaria mortality. One would have expected $k_1$ based on [13] to be larger than the $k_1=0.153$ estimate based on [19] because the former includes the impact of confounding factors; on the other hand, the 1.31 estimate may incorporate some reverse causality: malaria causes low WAZ and partial immunity (although seen more in older children), and so low WAZ may also be associated with less malaria. The relative risk of 1.31 is based on only two observational studies and has a 95 % CI of (0.92,1.88), which also is not quite at the level of statistical significance (p value = 0.143). Indeed, the relative risk of malaria due to undernutrition is difficult to estimate from observational studies [40].

Taken together, due to the nature of its design, the trial in [19] is believed to offer the best data for estimating the impact that undernutrition has on malaria prevalence. Although the p value of 0.177 does not allow for the traditional level of statistical significance, the biological plausibility of this hypothesis (e.g., undernutrition down-regulates immune functioning [41], including the anti-*P. falciparum* antibody response [42])—coupled with the similar results achieved in [39] and the important policy implications if it is true—leads us to believe that this problem is worthy of study despite the tenuous nature of the results. In summary, the results may not be valid and certainly are not robust, but they nonetheless deserve serious consideration. Given that no relevant data to shed more light on this issue are likely to be generated in the near future (in particular, there are ethical concerns with feeding trials that have treatment-free control arms), the most appropriate next step may be a randomized trial. More specifically, a design that may be ethically and politically acceptable is a cluster (at the village level) randomized control trial in a hypoendemic or mesoendemic setting, where the control arm offers a partial subsidy of ITNs to all children and the treatment arm provides free ITNs to children with WAZ $<-2$ and a partial subsidy to children with WAZ $>-2$.

## Conclusion

In calibrating the malaria-nutrition model, it is not possible to reliably estimate the proportion of susceptibility heterogeneity in the child population that is due to undernutrition: data from a randomized feeding trial generates a point estimate of 90.3 %, data from observational studies provide a point estimate of 5.3 %, and neither estimate is based on study results that are statistically significant at the traditional 0.05 level. The former estimate is assumed to be more reliable than the latter estimate due to the randomized nature of the design and its adjustment for other factors.

From a policy perspective, the results (Table 3) suggest that in a hypoendemic setting (EIR = 1), micro targeting of ITNs to undernutritioned children offers the most leverage; although targeting supplementary food offers some improvement on its own, the marginal impact of adding food to a targeted ITN policy is minimal. In the mesoendemic setting (EIR = 10), the impact of targeting ITNs is more modest, the impact of the targeted food policy increases with baseline ITN coverage, and the targeted ITN and food policy performs better than using either intervention alone. In the hyperendemic settings (EIR = 100 or 500), ITN targeting performs worse than the untargeted ITN policy, even though supplementary food has a larger impact than ITNs in these scenarios. The targeted food and ITN policy provides some improvement in these scenarios if coverage is high, although ITNs add little to this policy when baseline ITN coverage is already high.

The results from this analysis may not turn out to be true and are not robust. Nonetheless, the biological plausibility of the assumption underlying this result—coupled with the probable lack of new data to further inform this issue—leads us to suggest that a randomized cluster trial should be undertaken in a hypoendemic or mesoendemic setting, where children in the control group receive partially subsidized ITNs and children in the treatment group receive free ITNs if WAZ $<-2$ and partially subsidized ITNs if WAZ $>-2$.

## Additional file

10.1007/s12936-015-0894-x Details of the derivation of performance analysis measures for various policies, calibration of the model, and sensitivity analysis results.
