# Supplementary material for: Analysing the nutrition-disease nexus: the case of malaria
Source: Malar J. 2015 Dec 1;14:479. doi: 10.1186/s12936-015-0894-x (PMC4665912; doi:10.1186/s12936-015-0894-x)
Supplement: Supplementary file 1 — 10.1007/s12936-015-0894-x Details of the derivation of performance analysis measures for various policies, calibration of the model, and sensitivity analysis results. [file 12936_2015_894_MOESM1_ESM.pdf]

Additional file 1

Performance measures for the model are derived in §1 and the parameters for the model are estimated in §2. Figures 8-34 are cited in the main text.

1 Performance analysis

In this section, the prevalence of malaria infection P and the malaria mortality D for the five cases in Table 1 of the main text are derived. The clinical malaria prevalence is calculated from P via equation (33) of the main text.

1.1 No intervention

In the case of no intervention, $\phi_t^{(1)} = 0, \phi_t^{(2)} = 0$ (Table 1 in the main text), and ϕ_0^c is the proportion of children protected by ITNs. Hence, no children are in groups 12 and 22. Also, $f_S^{(11)}(s) = f_S^{(21)}(s) = f_S(s)$, which is the PDF for $S \sim \Gamma(k, 1/k)$. The equilibrium solution to (16), (18) and (20)-(22) in the main text is given by equations (24), (26) and (28)-(30) in the main text, where $\bar{h}$ in equation (31) in the main text reduces to

$$\bar{h} = \alpha \left((1 - \phi_c^0) \int_0^\infty \bar{x}_{11}(s) f_S(s)\ ds + p\phi_c^0 \int_0^\infty \bar{x}_{21}(s) f_S(s)\ ds \right) + (1-\alpha)[(1-\phi_a^1)\bar{w}_1 + p(1-\phi_a^1)\bar{w}_2]. \tag{1}$$

The pair $(\bar{y}, \bar{h})$ are solved using equation (30) in the main text and (1). By equation (32) in the main text and the identity $\mathcal{E} = a(\phi_N)m(\phi_N)\bar{y}$, the prevalence of malaria infection in children is

$$\begin{aligned} P &= (1 - \phi_0^c) \int_0^\infty (1 - e^{-bs\mathcal{E}/r}) f_S(s)\ ds + \phi_0^c \int_0^\infty (1 - e^{-bsp\mathcal{E}/r}) f_S(s)\ ds, \\ &= (1 - \phi_0^c) \left(1 - \left(1 + \frac{b\mathcal{E}}{rk} \right)^{-k} \right) + \phi_0^c \left(1 - \left(1 + \frac{bp\mathcal{E}}{rk} \right)^{-k} \right). \end{aligned} \tag{2}$$

To calculate the malaria mortality, $p_{11}(z) = P(I|Z_1 = z, G = 11)$ and $p_{21}(z) =$

$P(I|Z_1 = z, G = 21)$ need to be computed. Because the probability of being infected is given by $\bar{x}_{11}(s)$ in equation (24) in the main text, z is transformed into $u(z) = F_U^{-1}(1 - F_Z(z))$ and random v is added to get $s = u + v$, which gives

$$p_{11}(z) = \int_0^\infty P(I|Z = z, V = v, G = 11) f_V(v)\, dv, \tag{3}$$

$$= \int_0^\infty \bar{x}_{11}(u(z) + v) f_V(v)\, dv, \tag{4}$$

$$= 1 - e^{-bu(z)\mathcal{E}/r} \int_0^\infty \frac{v^{(k-k_1-1)} e^{-v\left(k+\frac{b\mathcal{E}}{r}\right)}}{\Gamma(k-k_1)\theta^{k-k_1}}\, dv \text{ by (24) in the main text,} \tag{5}$$

$$= 1 - e^{-bu(z)\mathcal{E}/r} \left(1 + \frac{b\mathcal{E}}{rk}\right)^{-(k-k_1)}. \tag{6}$$

A similar analysis yields

$$p_{21}(z) = 1 - e^{-bpu(z)\mathcal{E}/r} \left(1 + \frac{bp\mathcal{E}}{rk}\right)^{-(k-k_1)}. \tag{7}$$

Since there is no intervention, $f_{Z_1|G=11}(z) = f_{Z_1|G=21}(z) = f_Z(z)$. By equation (34) in the main text and equations (6)-(7), the malaria mortality is

$$D = P_c(\mathcal{E}) \int_0^\infty d(z) \left[(1 - \phi_0^c) p_{11}(z) + \phi_0^c p_{21}(z)\right] f_Z(z)\, dz. \tag{8}$$

1.2 Targeted food policy

Under the targeted food policy, $\phi_t^{(1)} = \phi_t^{(2)} = P(Z < \theta)$ (Table 1 in the main text), and this quantity is denoted by ϕ_t. Substituting the values for the probabilities for the targeted food

policy from Table 1 in the main text into equations (12)-(15) of the main text yields

$$f_S^{(11)}(s) = \frac{1}{1-\phi_t}\int_t^\infty f_V(s-u(z))f_Z(z)\ dz, \tag{9}$$

$$f_S^{(12)}(s) = \frac{1}{\phi_t}\int_{-\infty}^t f_V(s-u(z+a))\int_0^\infty f_Z(z)f_A(a)\ da\ dz, \tag{10}$$

$$f_S^{(21)}(s) = \frac{1}{1-\phi_t}\int_t^\infty f_V(s-u(z))f_Z(z)\ dz, \tag{11}$$

$$f_S^{(22)}(s) = \frac{1}{\phi_t}\int_{-\infty}^t f_V(s-u(z+a))\int_0^\infty f_Z(z)f_A(a)\ da\ dz. \tag{12}$$

Let $P_{ij} = P(I|G = ij)$ be the prevalence of malaria infection in group ij, so that $P_{ij} = \int_0^\infty \bar{x}_{ij}(s)f_S^{(ij)}(s)ds$ for $i, j = 1, 2$. It follows that

$$P_{11} = \int_0^\infty \bar{x}_{11}(s)f_S^{(11)}(s)\ ds, \tag{13}$$

$$= \int_0^\infty (1-e^{-bs\mathcal{E}/r})\frac{1}{1-\phi_t}\int_t^\infty f_V(s-u(z))f_Z(z)\ dz\ ds, \tag{14}$$

$$= \frac{1}{1-\phi_t}\int_0^\infty\int_t^\infty (1-e^{-bs\mathcal{E}/r})f_V(s-u(z))f_Z(z)\ dz\ ds, \tag{15}$$

$$= \frac{1}{1-\phi_t}\int_t^\infty \left(1-\left(1+\frac{b\mathcal{E}}{rk}\right)^{-k_2} e^{-b\mathcal{E}u(z)/r}\right) f_Z(z)\ dz. \tag{16}$$

A similar calculation gives

$$P_{21} = \frac{1}{1-\phi_t}\int_t^\infty \left(1-\left(1+\frac{bp\mathcal{E}}{rk}\right)^{-k_2} e^{-bp\mathcal{E}u(z)/r}\right) f_Z(z)\ dz. \tag{17}$$

The derivation for group 12 is

$$
\begin{aligned}
P_{12} &= \int_0^\infty \bar{x}_{12}(s) f_S^{(12)}(s)\, ds, &(18)\\
&= \int_0^\infty (1-e^{-bs\mathcal{E}/r}) \frac{1}{\phi_t} \int_{-\infty}^t f_V(s-u(z+a)) \int_0^\infty f_Z(z) f_A(a)\, da\, dz\, ds, &(19)\\
&= \frac{1}{\phi_t} \int_0^\infty \int_{-\infty}^t \left(\int_0^\infty (1-e^{-bs\mathcal{E}/r}) f_V(s-u(z+a))\, ds \right) f_Z(z) f_A(a)\, da\, dz, &(20)\\
&= \frac{1}{\phi_t} \int_0^\infty \int_{-\infty}^t \left(1 - \left(1+\frac{b\mathcal{E}}{rk}\right)^{-k_2} e^{-b\mathcal{E}u(z+a)/r} \right) f_Z(z) f_A(a)\, da\, dz, &(21)
\end{aligned}
$$

and a similar calculation leads to

$$
P_{22} = \frac{1}{\phi_t} \int_0^\infty \int_{-\infty}^t \left(1 - \left(1+\frac{bp\mathcal{E}}{rk}\right)^{-k_2} e^{-bp\mathcal{E}u(z+a)/r} \right) f_Z(z) f_A(a)\, da\, dz. \tag{22}
$$

By equations (30)-(31) in the main text, the pair $(\bar{y}, \bar{h})$ are derived using the following equations:

$$
\bar{y} = \frac{a(\phi_N)c\bar{h}}{a(\phi_N)c\bar{h} + \mu(\phi_N)}, \tag{23}
$$

$$
\begin{aligned}
\bar{h} &= \alpha\left((1-\phi_0^c)[(1-\phi_t)P_{11} + \phi_t P_{12}] + p\phi_0^c[(1-\phi_t)P_{21} + \phi_t P_{22}]\right)\\
&\quad + (1-\alpha)[(1-\phi_a^1)\bar{w}_1 + p\phi_a^1 \bar{w}_2].
\end{aligned} \tag{24}
$$

The prevalence of malaria infection is given by

$$
P = (1-\phi_0^c)\left[(1-\phi_t)P_{11} + \phi_t P_{12}\right] + \phi_0^c \left[(1-\phi_t)P_{21} + \phi_t P_{22}\right]. \tag{25}
$$

The group-specific PDFs $p_{11}(z)$, $p_{12}(z)$ are both given by (6) and $p_{21}(z)$, $p_{22}(z)$ are given by (7). For groups 11 and 21, since the two groups do not receive any food, $f_{Z_1|G=11} = f_{Z_1|G=21} = f_Z(z) 1_{\{z>t\}}$. For groups 12 and 22,

$$
f_{Z_1|G=12}(z) = f_{Z_1|G=22}(z) = \int_0^\infty f_Z(z-a) 1_{\{z-a<t\}} f_A(a) da \tag{26}
$$

By equation (34) in the main text, the malaria mortality is given by

$$D = (1-\phi_0^c)\left(\int_t^{\infty} d(z)P_c(\mathcal{E})p_{11}(z)f_Z(z)\ dz + \int_{-\infty}^{t}\int_0^{\infty} d(z+a)P_c(\mathcal{E})p_{12}(z+a)f_A(a)f_Z(z)\ dz\right)$$
$$+\,\phi_0^c\left(\int_t^{\infty} d(z)P_c(\mathcal{E})p_{21}(z)f_Z(z)\ dz + \int_{-\infty}^{t} d(z+a)P_c(\mathcal{E})p_{22}(z+a)f_Z(z)\ dz\right). \tag{27}$$

1.3 Untargeted ITN policy

Because this policy administers no food, it follows that $f_A(a) = \delta(a)$ and $f_S^{(11)}(s) = f_S^{(12)}(s) = f_S^{(21)}(s) = f_S(s)$, $f_S^{(22)}(s) = 0$. The prevalence of infection and the malaria mortality in this case is exactly the same as in the no-intervention scenario in §1.1, but with ϕ_0^c replaced by $\phi_0^c + (1-\phi_0^c)\phi$.

1.4 Targeted ITN policy

Substituting the values for the probabilities and $f_A(a)$ for the targeted ITN policy from Table 1 in the main text into equations (12)-(14) in the main text yields

$$f_S^{(11)}(s) = \frac{1}{1-\phi_t^{(1)}}\int_t^{\infty} f_V(s-u(z))f_Z(z)\ dz, \tag{28}$$

$$f_S^{(12)}(s) = \frac{1}{\phi_t^{(1)}}\int_{-\infty}^{t} f_V(s-u(z))f_Z(z)\ dz, \tag{29}$$

$$f_S^{(21)}(s) = \int_{-\infty}^{\infty} f_V(s-u(z))f_Z(z)\ dz = f_S(s). \tag{30}$$

Similar to the calculations in §1.2, the group prevalences are

$$P_{11} = \frac{1}{1-\phi_t^{(1)}} \int_t^{\infty} \left(1 - \left(1 + \frac{b\mathcal{E}}{rk}\right)^{-k_2} e^{-b\mathcal{E}u(z)/r}\right) f_Z(z)\ dz, \tag{31}$$

$$P_{12} = \frac{1}{\phi_t^{(1)}} \int_{-\infty}^{t} \left(1 - \left(1 + \frac{bp\mathcal{E}}{rk}\right)^{-k_2} e^{-bp\mathcal{E}u(z)/r}\right) f_Z(z)\ dz, \tag{32}$$

$$P_{21} = \int_0^{\infty} x_{21}(s) f_S(s)\ ds, \tag{33}$$

$$= \int_0^{\infty} (1 - e^{-bsp\mathcal{E}/r}) f_S(s)\ ds, \tag{34}$$

$$= 1 - \left(1 + \frac{bp\mathcal{E}}{rk}\right)^{-k}. \tag{35}$$

By equations (10)-(11) in the main text, the pair $(\bar{y}, \bar{h})$ are obtained by jointly solving

$$\bar{y} = \frac{a(\phi_N)c\bar{h}}{a(\phi_N)c\bar{h} + \mu(\phi_N)}, \tag{36}$$

$$\bar{h} = \alpha\left((1-\phi_0^c)[(1-\phi_t^{(1)})P_{11} + p\phi_t^{(1)}P_{12}] + \phi_0^c p P_{21}\right) + (1-\alpha)[(1-\phi_a^1)\bar{w}_1 + p\phi_a^1\bar{w}_2], \tag{37}$$

and the prevalence of malaria infection is

$$P = (1-\phi_0^c)[(1-\phi_t^{(1)})P_{11} + \phi_t^{(1)}P_{12}] + \phi_0^c P_{21}. \tag{38}$$

The PDF $p_{11}(z)$ is again given by (6), and $p_{12}(z)$ and $p_{21}(z)$ are both given by the right side of equation (7). Since, the policy administers no food, $f_{Z_1}(z) = f_Z(z)$. By equation (34) in the main text, the malaria mortality is

$$\begin{aligned} D = (1-\phi_0^c)&\left(\int_t^{\infty} d(z)P_c(\mathcal{E})p_{11}(z)f_Z(z)\ dz + \int_{-\infty}^{t} d(z)P_c(\mathcal{E})p_{12}(z)f_Z(z)\ dz\right) \\ &+ \phi_0^c\left(\int_{-\infty}^{\infty} d(z)P_c(\mathcal{E})p_{21}(z)f_Z(z)\ dz\right). \end{aligned} \tag{39}$$

1.5 Targeted ITN and targeted food policy

As in §1.2, $\phi_t^{(1)} = \phi_t^{(2)} = P(Z < \theta)$, which is denoted by ϕ_t. The calculation of P_{ij} is similar to that of the targeted food policy with two differences: $\phi_N = 2\alpha(\phi_0^c + \phi_t(1-\phi_0^c))$ and group 12 is protected by ITNs as well, and hence the biting rate of the mosquitoes is reduced by a factor of p. This implies

$$P_{11} = \frac{1}{1-\phi_t}\int_t^\infty \left(1 - \left(1 + \frac{b\mathcal{E}}{rk}\right)^{-k_2} e^{-b\mathcal{E}u(z)/r}\right) f_Z(z)\, dz, \tag{40}$$

$$P_{21} = \frac{1}{1-\phi_t}\int_t^\infty \left(1 - \left(1 + \frac{bp\mathcal{E}}{rk}\right)^{-k_2} e^{-bp\mathcal{E}u(z)/r}\right) f_Z(z)\, dz, \tag{41}$$

$$P_{12} = P_{22} = \frac{1}{\phi_t}\int_0^\infty \int_{-\infty}^t \left(1 - \left(1 + \frac{bp\mathcal{E}}{rk}\right)^{-k_2} e^{-bp\mathcal{E}u(z+a)/r}\right) f_Z(z) f_A(a)\, da\, dz. \tag{42}$$

The pair $(\bar{y}, \bar{h})$ are obtained by solving

$$\bar{y} = \frac{a(\phi_N)c\bar{h}}{a(\phi_N)c\bar{h} + \mu(\phi_N)}, \tag{43}$$

$$\bar{h} = \alpha\left((1-\phi_0^c)[(1-\phi_t)P_{11} + p\phi_t P_{12}] + \phi_0^c p[(1-\phi_t)P_{21} + \phi_t P_{22}]\right) + (1-\alpha)[(1-\phi_a^1)\bar{w}_1 + p\phi_a^1\bar{w}_2], \tag{44}$$

and the prevalence of malaria infection is

$$P = (1-\phi_0^c)[(1-\phi_t)P_{11} + \phi_t P_{12}] + \phi_0^c[(1-\phi_t)P_{21} + \phi_t P_{22}]. \tag{45}$$

The group-specific PDFs are

$$p_{11}(z) = 1 - e^{-bu(z)\mathcal{E}/r}\left(1 + \frac{b\mathcal{E}}{rk}\right)^{-(k-k_1)}, \tag{46}$$

$$p_{12}(z) = p_{21}(z) = p_{22}(z) = 1 - e^{-bpu(z)\mathcal{E}/r}\left(1 + \frac{bp\mathcal{E}}{rk}\right)^{-(k-k_1)}. \tag{47}$$

Similar to the targeted food case, the group-specific distributions of WAZ values are

$$f_{Z_1|G=11} = f_{Z_1|G=21} = f_Z(z)1_{\{z>t\}}, \tag{48}$$

$$f_{Z_1|G=12}(z) = f_{Z_1|G=22}(z) = \int_0^\infty f_Z(z-a)1_{\{z-a<t\}}f_A(a)\ da, \tag{49}$$

and the malaria mortality is

$$D = (1-\phi_0^c)\left(\int_t^\infty d(z)P_c(\mathcal{E})p_{11}(z)f_Z(z)\ dz + \int_{-\infty}^t\int_0^\infty d(z+a)P_c(\mathcal{E})p_{12}(z+a)f_A(a)f_Z(z)\ da\ dz\right) + \phi_0^c\left(\int_t^\infty d(z)P_c(\mathcal{E})p_{21}(z)f_Z(z)\ dz + \int_{-\infty}^t\int_0^\infty d(z+a)P_c(\mathcal{E})p_{22}(z+a)f_Z(z)\ dz\right). \tag{50}$$

2 Parameter estimation

The values for μ_A, k_1 and the probability of clinical disease upon infection $p_c(\mathcal{E})$ are derived in §2.1, §2.3 and §2.4, respectively, and the ITN functions and the mortality function $d(z)$ are derived in §2.2 and §2.5. An alternative approach to estimating k_1 is given in §2.6.

2.1 Estimating μ_A

Consider a typical child from [1] that has mean age, mean baseline HAZ and mean baseline WHZ from their population of children, which are -1.9, -0.7 and 30 mo, respectively (Table 1 of [1]). These values correspond to baseline heights of 84.64 cm (girls) and 86.09 cm (boys), and baseline weights of 10.63 kg (girls) and 11.29 kg (boys), which in turn yield baseline WAZ values of -1.58 (girls) and -1.50 (boys). The average increase (after eight mo) in HAZ and WHZ from the three mo of supplementary food was 0.14 and 0.22, respectively, giving post-intervention values of HAZ = -1.76 and WHZ = -0.48. These values correspond to post-intervention heights of 90.13 cm (girls) and 91.23 cm (boys) and post-intervention weights of 12.15 kg (girls) and 12.68 kg (boys). These weights yield post-intervention WAZ values of -1.33 (girls) and -1.29 (boys). Therefore, the increases in WAZ for the typical child are

0.25 (girls) and 0.21 (boys), and the mean WAZ increase is set to 0.23.

2.2 Estimating the ITN functions

The functions $a(\phi_N)$, $m(\phi_N)$ and $\mu(\phi_N)$ are derived in [2] using a feeding cycle model. In this feeding cycle model, p is the probability that a mosquito successfully bites after finding a human under an ITN, r_l is the probability that a mosquito looks for another host after finding a human under an ITN, $Q(\phi_N)$ is the proportion of mosquito blood meals taken on a human, $1/f(\phi_N)$ is the mean time to complete one feeding cycle, and $\tau_1(\phi_N)$ and τ_2 are the lengths of the host-seeking and oviposition stages of the feeding cycle. The feeding cycle model in [2] specifies that

$$Q(\phi_N) = \frac{Q(0)(1-\phi_N+\phi_N p)}{1-Q(0)\phi_N(1-p)}, \tag{51}$$

$$\frac{1}{f(\phi_N)} = \frac{\tau_1(0)}{1-Q(0)\phi_N r_l} + \tau_2, \tag{52}$$

$$a(\phi_N) = f(\phi_N)Q(\phi_N), \tag{53}$$

$$= \frac{(1-Q(0)\phi_N r_l)Q(0)(1-\phi_N+\phi_N p)}{[\tau_1(0)+\tau_2(1-Q(0)\phi_N r_l)][1-Q(0)\phi_N(1-p)]} \quad \text{by (32) – (33).} \tag{54}$$

The parameter values in [2] corresponding to the species *Anopheles gambiae s.l.* in Nigeria are used because it transmits *Plasmodium falciparum*. From [2], $Q(0) = 0.9, p = 0.1, r_l = 0.6$, $\tau_1(0) = 0.7667$ days and $\tau_2 = 1.2333$ days, and substituting these values into (34) yields the biting rate function $a(\phi_N)$ in Figure 1.

Let $p_1(0)$ be the probability of surviving the host-seeking process of the feeding cycle in the absence of ITNs, p_2 be the probability of surviving the resting cycle, and λ be the rate at which mosquitoes emerge per human per day. In [2], the ratio of the number of female mosquitoes to that of humans is given by

$$m(\phi_N) = \frac{\lambda}{-f(\phi_N)\ln\left(\frac{p_1(0)p_2[1-Q(0)\phi_N(1-p)]}{1-Q(0)\phi_N r_l p_1(0)}\right)}. \tag{55}$$

Equating the estimated mean life span of a mosquito under ITN coverage ϕ_N in [2] to the analytical value from the feeding cycle model, which is $m(\phi_N)/\lambda$, yields $p_1(0) = 0.9427$ and $p_2 = 0.8592$.

For a given λ, the pre-intervention EIR is obtained by solving for $\bar{y}$ as in § 1.1 and setting $\mathcal{E} = a(0)m(0)\bar{y}$. The parameter λ in (55) is varied to generate the scenarios with pre-intervention EIR equaling 1, 10, 100 and 500, which yields $\lambda = 0.757, 4.644, 45.634$ and 226.405, respectively. The function $m(\phi_N)$ for these four scenarios appears in Figure 2.

The mosquito mortality rate varies with ITN coverage according to

$$\mu(\phi_N) = \mu(0) + a(\phi_N)\phi_N d, \tag{56}$$

where d is the probability that a mosquito is killed by insecticide after finding a human under a ITN. It is assumed that $d = 0.3$ from [2] and $\mu(0) = 0.2$/day from Table 4 in [3], which yields the mosquito mortality function in Figure 3.

2.3 Estimating k_1

Let P_0 denote the prevalence in the intervention-free arm and let P_1 denote the prevalence in the food intervention arm; in [1], food was given to every child in the latter arm regardless of nutritional status. The odds ratio is defined by

$$\text{OR} = \frac{P_1/(1-P_1)}{P_0/(1-P_0)}. \tag{57}$$

Define the prevalence ratio PR by P_1/P_0, so that

$$\text{PR} = \frac{\text{OR}}{(1-P_0) + \text{OR}P_0}. \tag{58}$$

Equation (24) can be used to compute that the (adjusted) odds ratio of 0.76 corresponds to a prevalence ratio of 0.77. For the non-intervention arm, the scenario is the same as in §1.1

with $\phi_0^c = 0.55$, which is the bed net coverage from a survey of 3113 children under five from Niger in Sept-Oct 2006 [7], which is the same time and location as the study in [1]; the bed net coverage in [1] was nearly the same, at 53% (personal communication, Sheila Isanaka, June 3, 2015). The value of $\bar{y}$ is obtained from equation (30) in the main text and (1), and the prevalence in the child population is given by equation (2) as

$$P_0 = (1-\phi_0^c)\left(1-\left(1+\frac{b\mathcal{E}_0}{rk}\right)^{-k}\right) + \phi_0^c\left(1-\left(1+\frac{bp\mathcal{E}_0}{rk}\right)^{-k}\right). \tag{59}$$

where $\mathcal{E}_0$ is the EIR in the non-intervention arm. Similarly, it follows by equations (6)-(7) that

$$\begin{aligned} P_1 = (1-\phi_0^c)&\left(1-\left(1+\frac{b\mathcal{E}_1}{rk}\right)^{-(k-k_1)}\int_{-\infty}^{\infty} e^{-bu(z)\mathcal{E}_1/r} f_{Z_1}(z)\ dz\right) \\ +\ \phi_0^c&\left(1-\left(1+\frac{bp\mathcal{E}_1}{rk}\right)^{-(k-k_1)}\int_{-\infty}^{\infty} e^{-bu(z)p\mathcal{E}_1/r} f_{Z_1}(z)\ dz\right), \end{aligned} \tag{60}$$

where $\mathcal{E}_1$ is the EIR in the treatment arm and

$$f_{Z_1}(z) = \int_0^\infty f_Z(z-a) f_A(a)\ da. \tag{61}$$

The parameter k_1 is chosen so that $\frac{p_C(\mathcal{E}_1)P_1}{p_C(\mathcal{E}_0)P_0} = 0.77$, where P_0 and P_1 are the solutions to equations (59)-(60). In performing this calculation, in order to use the best estimate of the WAZ PDF for the child population in [1], for this calculation only, μ_Z is changed from -1.00 to -1.54, which is the average of -1.58 (girls) and -1.50 (boys) (see §2.1); for lack of data on σ_Z in the population in [1], σ_Z is set to 1.10 because the expected range of σ_Z is [1.00,1.20] (pg 51 of [4]). The parameter λ in (55) is chosen so that $m(2\alpha\phi_0^c) = 10.6848$, which generates (using equation (30) in the main text and (1)-(2)) the intervention-free malaria prevalence of 0.0564 from [1]. Solving $\frac{p_C(\mathcal{E}_1)P_1}{p_C(\mathcal{E}_0)P_0} = 0.77$ gives $k_1 = 0.153$. The resulting PDFs $f_S(s)$, $f_U(u)$ and $f_V(v)$ are plotted in Figure 4.

2.4 Estimating $p_c(\mathcal{E})$

The function $p_c(\mathcal{E})$ is estimated from a model that specifies $p_c(a, \mathcal{E})$, which is the conditional probability of acquiring clinical disease given malaria infection, as a function of age a and EIR $\mathcal{E}$ [8]. A Hill function,

$$p_c(a, \mathcal{E}) = \frac{1}{1 + \left(\frac{I_C(a)}{I_{C0}(\mathcal{E})}\right)^{\kappa_c}}, \tag{62}$$

is used in [8], where $I_{C0}(\mathcal{E})$, which is given in §3.1.2 of Protocol S3 in [8], and $\kappa_c = 4.13$ are estimated by fitting the model to data, and the time-dependent clinical immunity $I_C(a, t)$ is the sum of immunity acquired by exposure to infection ($I_{CA}(a, t)$) and that maternally acquired ($I_{CM}(a, t)$). These components are modeled by the partial differential equations,

$$\frac{\partial I_{CA}(a, t)}{\partial t} + \frac{\partial I_{CA}(a, t)}{\partial a} = \frac{\mathcal{E}}{\gamma\mathcal{E} + 1} - \frac{I_{CA}(a, t)}{d_c}, \qquad I_{CA}(0, t) = 0, \tag{63}$$

$$\frac{\partial I_{CM}(a, t)}{\partial t} + \frac{\partial I_{CM}(a, t)}{\partial a} = -\frac{I_{CM}(a, t)}{d_M}, \quad I_{CM}(0, t) = P_{CM} I_C(20, t), \tag{64}$$

where $d_C = 30$ years and $d_M = 255.5$ days are the reciprocals of the decay rates of infection-acquired immunity and maternally-acquired immunity, respectively (Table S3.1 in [8]), and $P_{CM} = 0.5$ is the proportion of the maternal immunity that a new-born acquires. Solving for the equilibrium values of these immunity functions as a function of age (by setting $\frac{\partial I_{CA}(a,t)}{\partial t} = 0$ and $\frac{\partial I_{CM}(a,t)}{\partial t} = 0$) yields (introducing the dependence on $\mathcal{E}$)

$$I_{CA}(a, \mathcal{E}) = \frac{\mathcal{E}}{\gamma\mathcal{E} + 1} d_c \left(1 - e^{-a/d_c}\right), \tag{65}$$

$$I_{CM}(a, \mathcal{E}) = P_{CM} I_C(20) e^{-a/d_M}, \tag{66}$$

and therefore

$$I_C(a,\mathcal{E}) = I_{CA}(a,\mathcal{E}) + I_{CM}(a,\mathcal{E}), \tag{67}$$

$$= \frac{\mathcal{E}}{\gamma\mathcal{E}+1} d_c \left(1 - e^{-a/d_c}\right) + P_{CM} I_C(20,\mathcal{E}) e^{-a/d_M}. \tag{68}$$

For $a = 20$ years, $e^{-a/d_M} \approx 0$, and by (68) it is assumed that $I_C(20,\mathcal{E}) = \frac{\mathcal{E}}{\gamma\mathcal{E}+1} d_c \left(1 - e^{-2/3}\right)$. It follows that

$$I_C(a,\mathcal{E}) = \frac{\mathcal{E}}{\gamma\mathcal{E}+1} d_c \left(1 - e^{-a/d_c} + P_{CM}(1 - e^{-2/3}) e^{-a/d_M}\right). \tag{69}$$

Substituting (69) into (62) gives $p_c(a,\mathcal{E})$. Because this function does not vary much between ages six months and five years when EIR > 1 (Figure 5), the age dependence is ignored and the function $p_c(\mathcal{E})$ is calculated by integrating $p_c(a,\mathcal{E})$ between the ages of six months and five years:

$$p_c(\mathcal{E}) = \int_{0.5}^{5} \frac{1}{1 + \left(\frac{I_C(a,\mathcal{E})}{I_{C0}(\mathcal{E})}\right)^{\kappa_C}}\, da. \tag{70}$$

The resulting function is plotted in Figure 6.

2.5 Estimating $d(z)$

Let D be the event of death from malaria, I be the event of malaria infection, and Z be the random WAZ value. It follows that

$$d(z) = P[D|I, Z = z] = \frac{P[D, I|Z = z]}{P[I|Z = z]}. \tag{71}$$

The numerator in equation (71), which is the number of malaria deaths per 1000 children in the entire (infected and uninfected) population, has been estimated to be (Table 2.5 in [5])

$$P[D, I|Z = z] = e^{0.058 - 0.750z}. \tag{72}$$

The denominator in equation (71) is assumed to take the form $P[I|Z=z]=e^{a_0+a_1 z}$ and the parameters a_0 and a_1 are derived based on the estimate (Table 2.8 in [5]) that the relative risk of malaria infection associated with WAZ < -2 is 1.31. This relative risk is given by

$$\frac{P[I|Z<-2]}{P[I|Z>2]} = \frac{\int_{-\infty}^{-2} e^{a_0+a_1 z} f_Z(z)dz / F_Z(-2)}{\int_{-2}^{\infty} e^{a_0+a_1 z} f_Z(z)dz / [1-F_Z(-2)]}, \tag{73}$$

$$= \frac{[1-F_Z(-2)] \int_{-\infty}^{-2} e^{a_1 z} f_Z(z)dz}{F_Z(-2) \int_{-2}^{\infty} e^{a_1 z} f_Z(z)dz}. \tag{74}$$

Equating the expression in (74) to 1.31 yields the value of a_1, but first $f_Z(z)$ needs to be determined. In this subsection only, $f_Z(z)$ is the WAZ PDF for the population of children in [6], which is the study that dominates the meta-analysis in Table 2.8 of [5].

The population in [6] consists of 397 children in the mesoendemic area and 1114 children in hyperendemic area. The two WAZ PDFs of these two areas in Figure 1 of [6] are fit to the distributions $\mathcal{N}(-0.77, 1.01)$ and $\mathcal{N}(-0.99, 1.07)$. Denoting these two PDFs by $f_m(z)$ and $f_h(z)$ yields the mixture density

$$f_Z(z) = \frac{397}{397+1114} f_m(z) + \frac{1114}{397+1114} f_h(z). \tag{75}$$

Substituting (75) into (74) and equating (74) to 1.31 gives $a_1 = -0.143$.

The parameter a_0 is estimated by assuming that children do not get infected twice within six months, and that the likelihood of children with WAZ < -2 getting infected with malaria is 31/129 (Table 2 in [6]). Solving

$$\frac{31}{129} = \frac{\int_{-\infty}^{-2} e^{a_0+a_1 z} f_Z(z)dz}{\int_{-\infty}^{-2} f_Z(z)dz} \tag{76}$$

gives $a_0 = -1.793$.

Combining equations (71)-(72) and the estimates of a_0 and a_1 gives

$$d(z) = \frac{e^{0.058-0.750z}}{e^{-1.793-0.143z}} = e^{1.851-0.607z} \tag{77}$$

per 1000 children, which is plotted in Figure 7.

2.6 Alternative approach to estimating k_1

This section contains an alternative approach to estimating k_1, based on the relative risk of malaria infection associated with WAZ < -2 being equal to 1.31 [5]. Because $Z < -2$ corresponds to $U > u(-2) = F_U^{-1}(1 - F_Z(-2))$, the relative risk is given by

$$\frac{P[I|U > u(-2)]}{P[I|U < u(-2)]}. \tag{78}$$

The numerator of (78) can be expressed as

$$\begin{aligned}
P[I|U > u(-2)] &= \frac{P[I, U > u(-2)]}{F_Z(-2)} && (79)\\
&= \frac{1}{F_Z(-2)} \int\limits_{u(-2)}^{\infty} P(I, U = u)\ du, && (80)\\
&= \frac{1}{F_Z(-2)} \int\limits_{u(-2)}^{\infty} \int\limits_{0}^{\infty} \left(1 - e^{-b(u(z)+v)\mathcal{E}/r}\right) f_V(v) f_U(u)\ dv\ du \quad \text{by (??) and (4)}, && (81)\\
&= \frac{1}{F_Z(-2)} \left(F_Z(-2) - \int\limits_{u(-2)}^{\infty} e^{-bu(z)\mathcal{E}/r} f_U(u)\ du \int\limits_{0}^{\infty} e^{-bv\mathcal{E}/r} f_V(v)\ dv \right), && (82)\\
&= \frac{1}{F_Z(-2)} \left(F_Z(-2) - \left(1 + \frac{b\mathcal{E}}{rk}\right)^{-(k-k_1)} \int\limits_{u(-2)}^{\infty} e^{-bu(z)\mathcal{E}/r} f_U(u)\ du \right) \quad \text{by (4)} - (6). && (83)
\end{aligned}$$

Similarly, the denominator of (78) is given by

$$P[I|U < u(-2)] = \frac{1}{1 - F_Z(-2)} \left((1 - F_Z(-2)) - \left(1 + \frac{b\mathcal{E}}{rk}\right)^{-(k-k_1)} \int_0^{u(-2)} e^{-bu(z)\mathcal{E}/r} f_U(u)\, du \right). \tag{84}$$

Combining equations (78), (83) and (84) yields the equation that needs to be solved:

$$\frac{(1 - F_Z(-2)) \left(F_Z(-2) - \left(1 + \frac{b\mathcal{E}}{rk}\right)^{-(k-k_1)} \int_{u(-2)}^{\infty} e^{-bu(z)\mathcal{E}/r} f_U(u)\, du \right)}{F_Z(-2) \left((1 - F_Z(-2)) - \left(1 + \frac{b\mathcal{E}}{rk}\right)^{-(k-k_1)} \int_0^{u(-2)} e^{-bu(z)\mathcal{E}/r} f_U(u)\, du \right)} = 1.31. \tag{85}$$

To reflect the population in [5, 6], $f_Z(z)$ in (85) is set to (75) (and $F_Z(z)$ is set accordingly), and λ in (55) is calibrated so that $m(0)$ satisfies $p_c(\mathcal{E})P(I|Z < -2) = 31/129$, which by (83), is

$$p_c(\mathcal{E}) \frac{1}{F_Z(-2)} \left(F_Z(-2) - \left(1 + \frac{b\mathcal{E}}{rk}\right)^{-(k-k_1)} \int_{u(-2)}^{\infty} e^{-bu(z)\mathcal{E}/r} f_U(u)\, du \right) = \frac{31}{129}. \tag{86}$$

Solving (84)-(86) jointly for k_1 and m gives $k_1 = 0.0095$ and $m = 13.78$.

References

[1] Isanaka, S., Nombela, N., Djibo, A., Poupard, M., Van Beckhoven, D., Gaboulaud, V. *et al.*. **Effective of preventive supplementation with ready-to-use therapeutic food on the nutritional status, mortality and morbidity of children aged 6 to 60 months in Niger**. *JAMA* **301**, 277-285, 2009.

[2] Le Menach, A., Takala, S., McKenzie, F. E., Perisse, A., Harris, A., Flahault, A. *et al.*. **An elaborated feeding cycle model for reductions in vectorial capacity of night-biting mosquitoes by insecticide-treated nets**. *Malaria Journal* **6**, 10, 2007.

[3] Mandal, S., Sarkar, R. R., Sinha, S. **Mathematical models of malaria - a review**. *Malaria Journal* **10**, 202, 2011.

[4] de Onis, M., Blossner, M. *WHO global database on child growth and malnutrition.* World Health Organization, Geneva, 1997.

[5] Fishman, S. M., Caulfield, L. E., de Onis, M., Blossner, M., Hyder, A. A., Mullany, L. *et al.*. **Childhood and maternal underweight**. Chapter 2 of *Comparative quantification of health risks: global and regional burden of disease attributable to selected major risk factors, Vol. 1.* Eds, Ezzati, M., Lopez, A. D., Rodgers, A., Murray, C. J. L. World Health Organization, Geneva, 2004.

[6] Williams, T. N., Maitland, K., Phelps, L., Bennett, S., Peto, T. E. A., Viji, J. *et al.*. **Plasmodium vivax: a cause of malnutrition in young children**. *Quarterly Journal Medicine* **90**, 751-757, 1997.

[7] Vanden Eng, J. L., Thwing, J., Wolkon, A., Kulkarni, M. A., Manya, A., Erskine, M. *et al.*. **Assessing bed net use and non-use after long-lasting insecticidal net distribution: a simple framework to guide programmatic strategies**. *Malaria Journal* **9**, 133, 2010.

[8] Griffin, J. T., Hollingsworth, T. D., Okell, L. C., Churcher, T. S. White, M., Hinsley, W., et al. **Reducing Plasmodium falciparum malaria transmission in Africa: a model-based evaluation of intervention strategies**. *PLOS Medicine* **7**, e1000324, 2010.

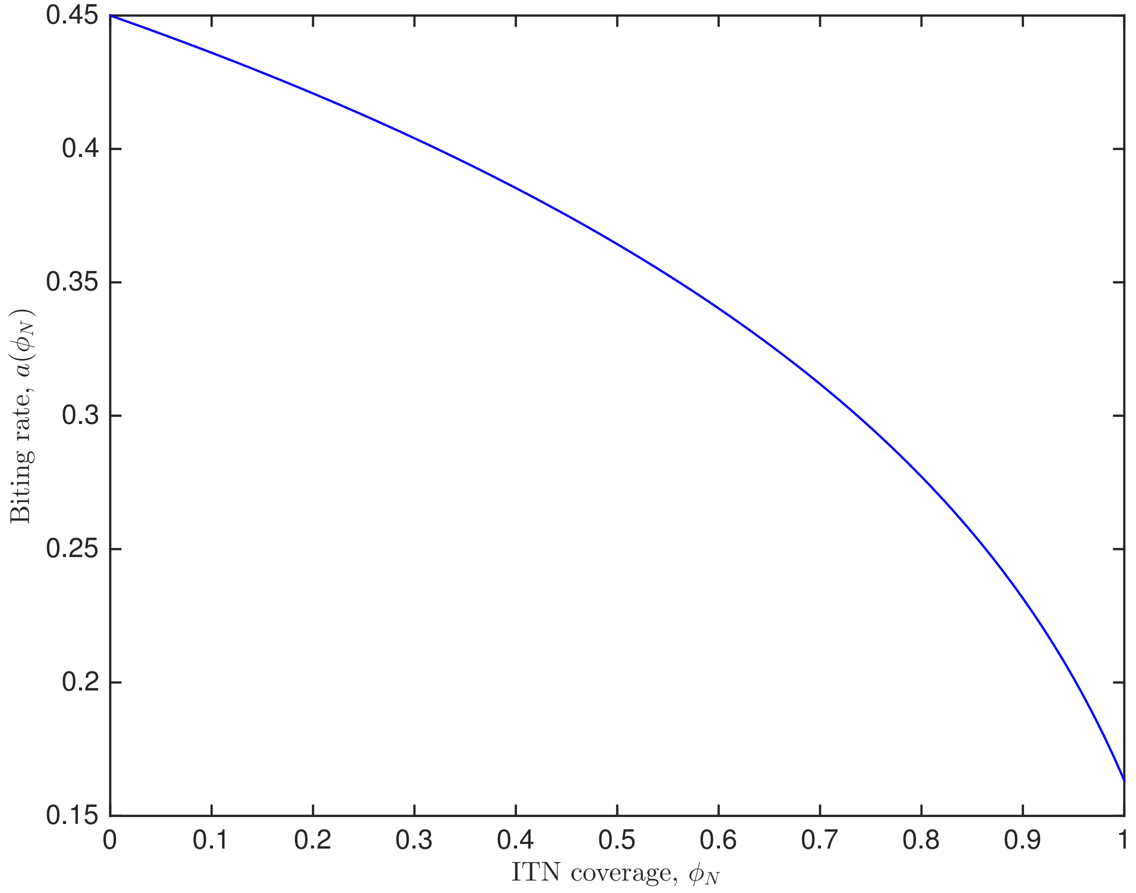

Figure 1: The biting rate $a(\phi_N)$ as a function of the ITN coverage ϕ_N.

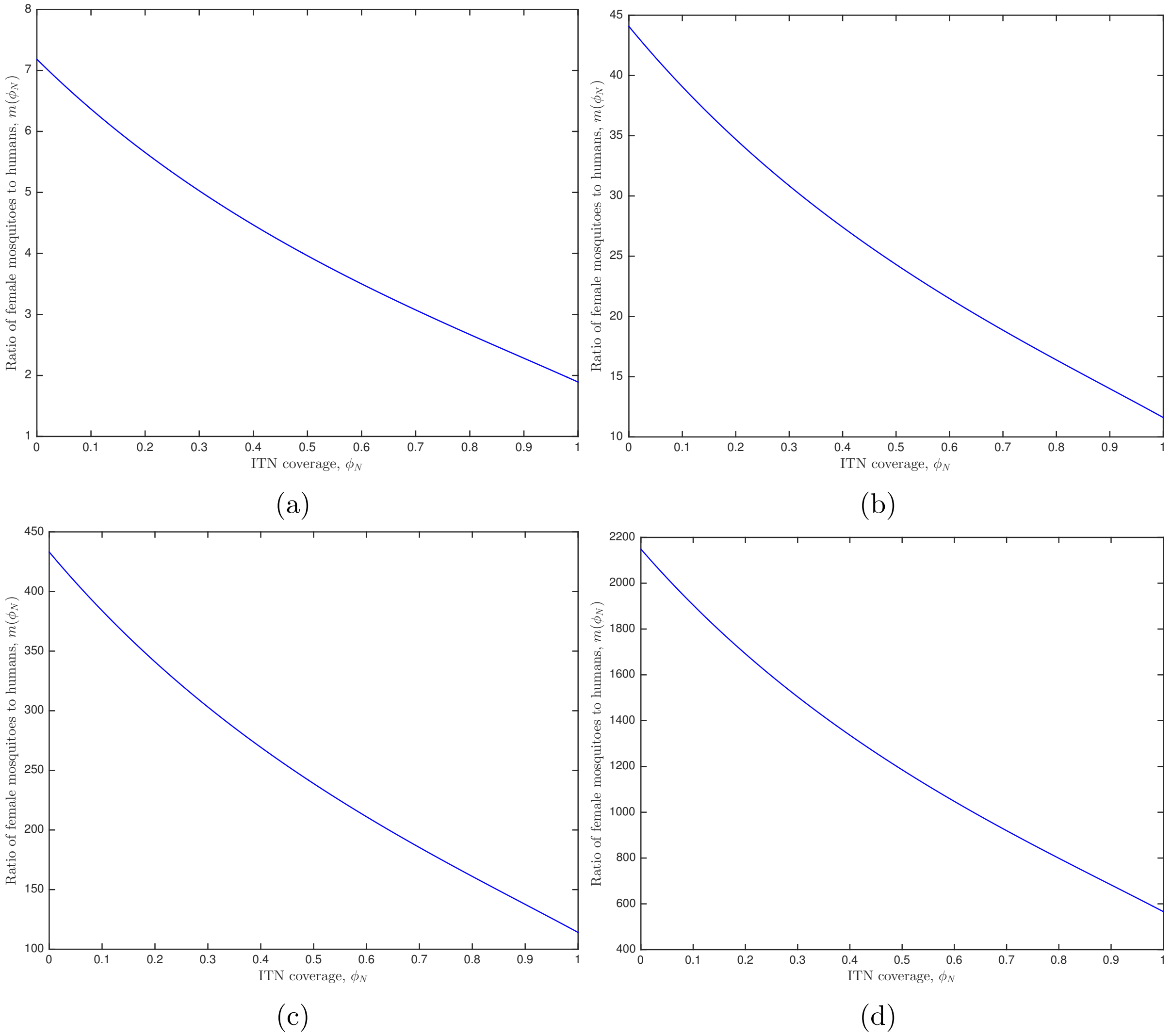

Figure 2: The ratio of female mosquitoes to humans, $m(\phi_N)$, as a function of the ITN coverage ϕ_N for pre-intervention EIR equal to **(a)** 1, **(b)** 10, **(c)** 100, and **(d)** 500.

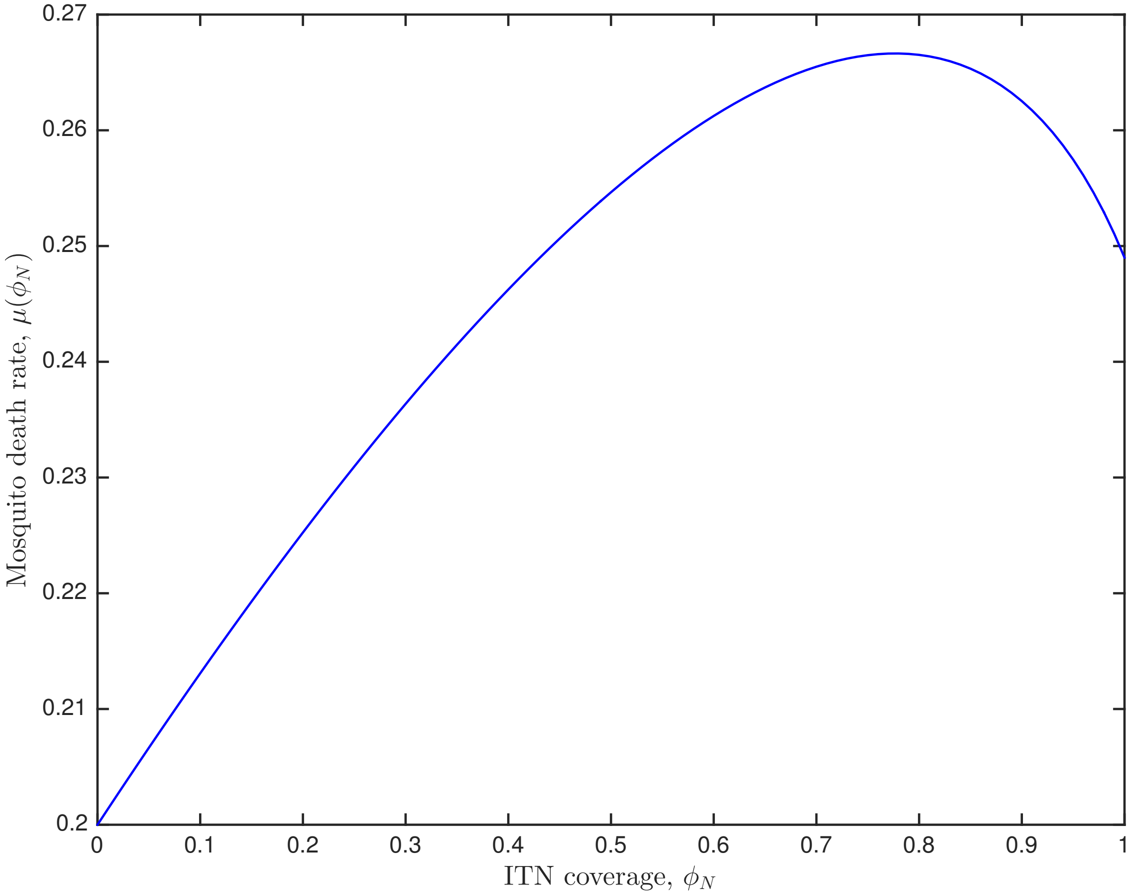

Figure 3: The mosquito death rate $\mu(\phi_N)$ as a function of the ITN coverage ϕ_N.

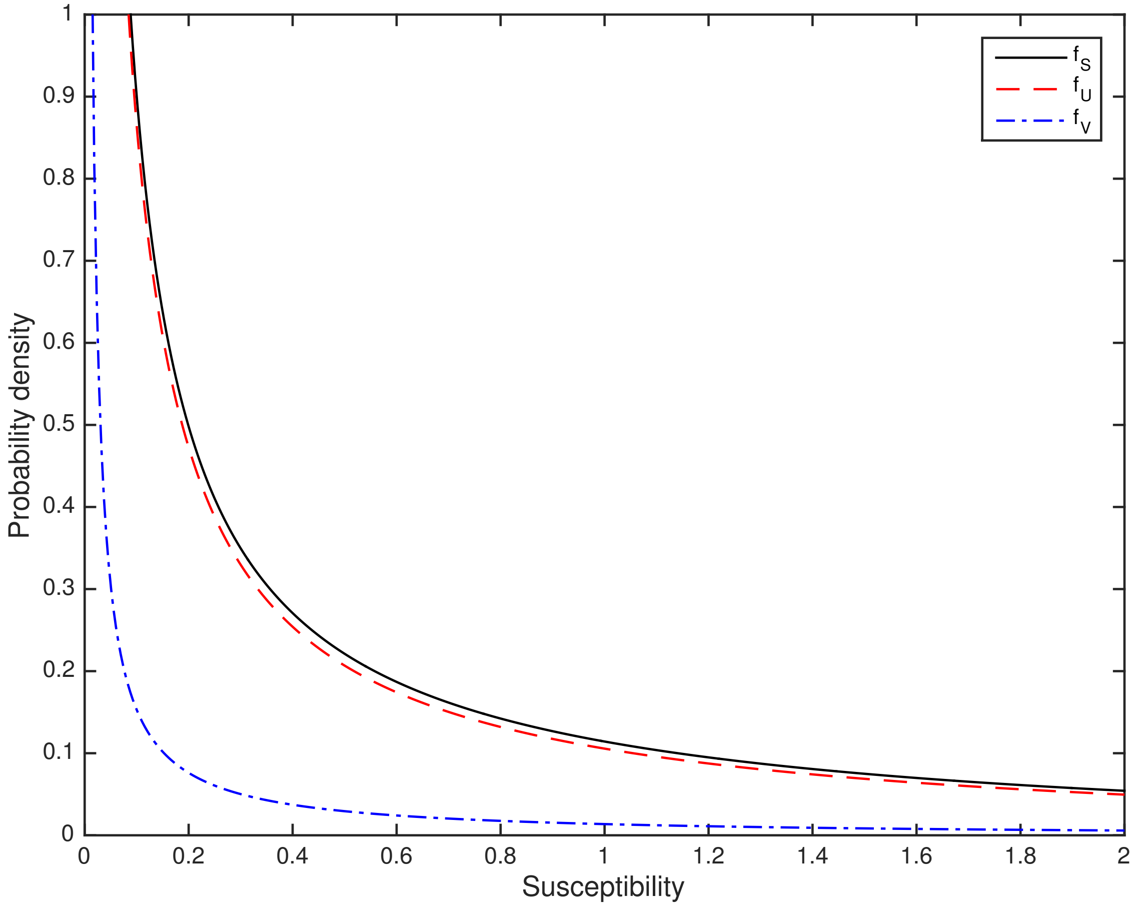

Figure 4: The PDFs of $f_S(s)$ (total susceptibility in the human population), $f_U(u)$ (susceptibility due to undernutrition) and $f_V(v)$ (susceptibility that is independent of undernutrition).

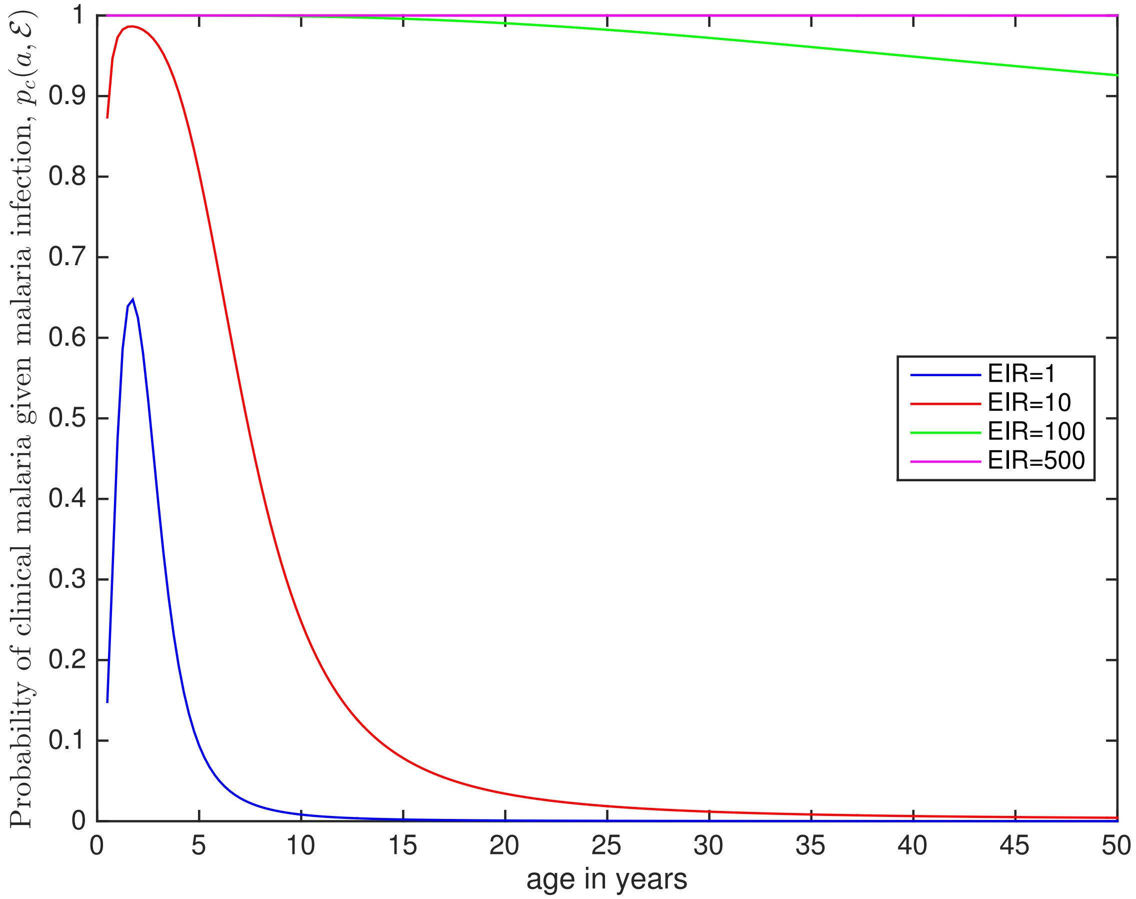

Figure 5: The conditional probability of clinical malaria given malaria infection, $p_c(a, \mathcal{E})$, as a function of age a for four values of EIR.

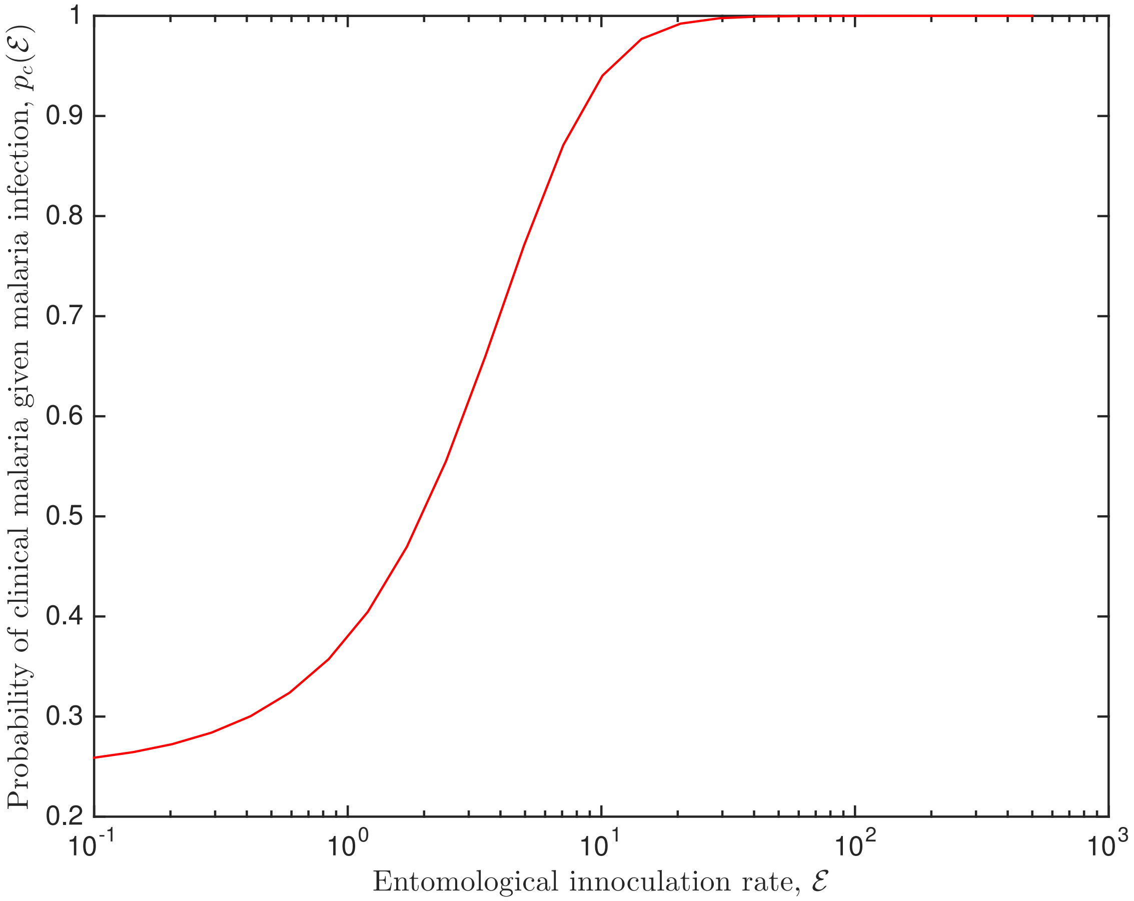

Figure 6: The conditional probability of clinical malaria given malaria infection, $p_c(\mathcal{E})$, as a function of EIR $\mathcal{E}$.

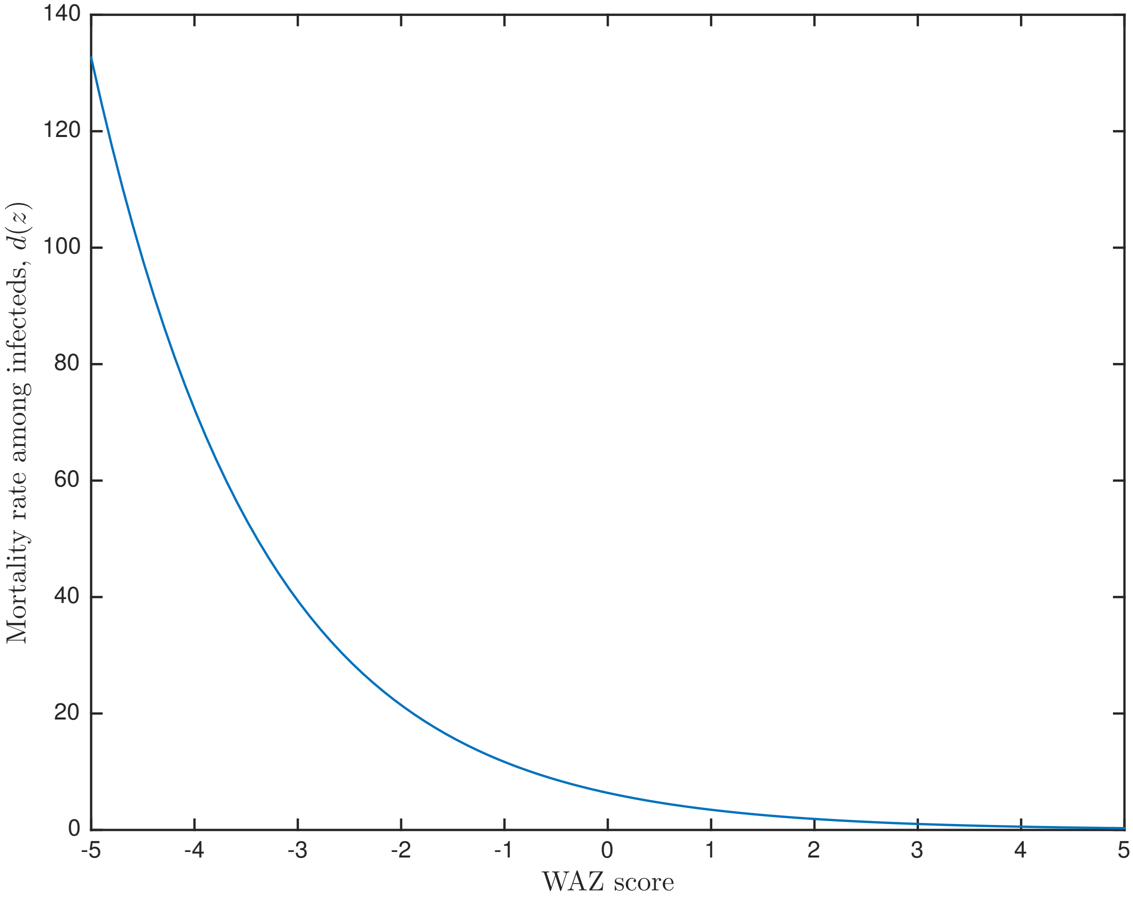

Figure 7: The mortality rate (per 1000 children) among infecteds as a function of WAZ, $d(z)$.

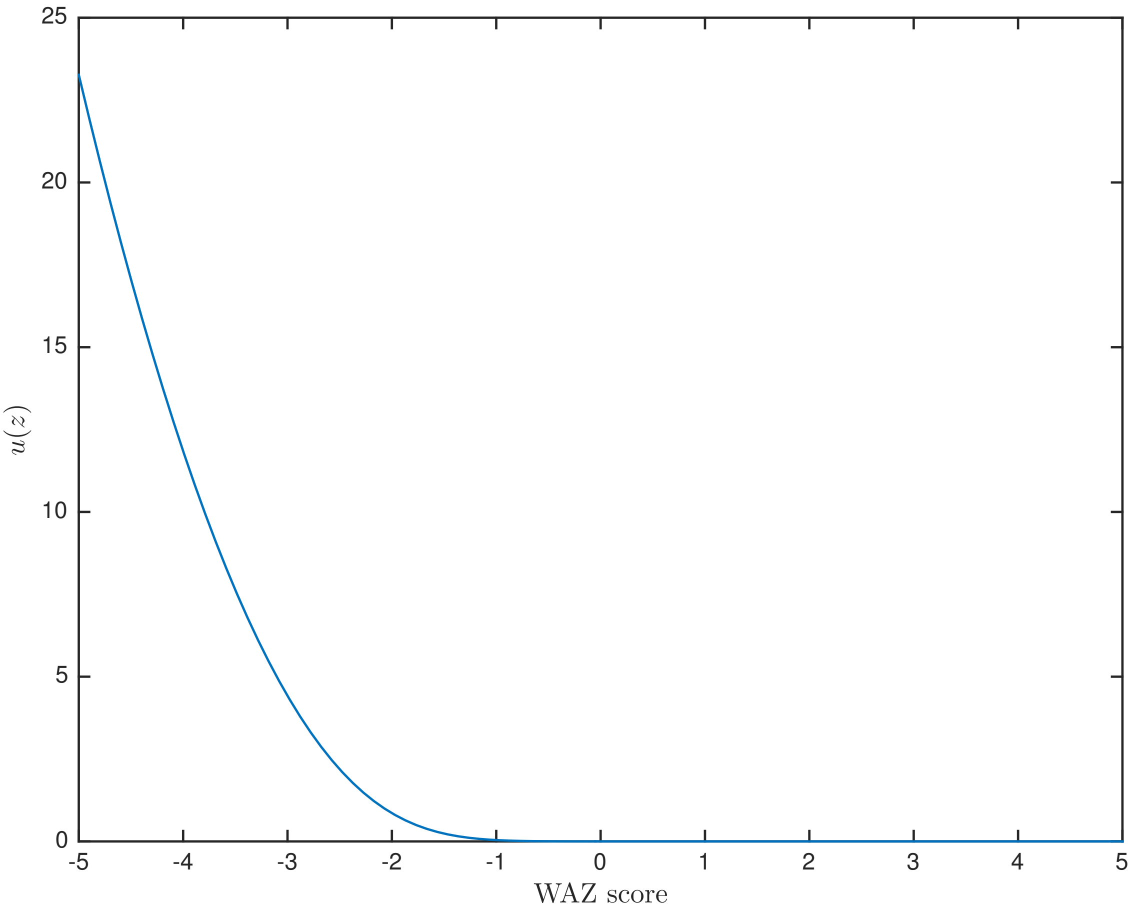

Figure 8: The function $u(z) = F_U^{-1}(1 - F_Z(z))$, which transforms WAZ values into undernutrition values.

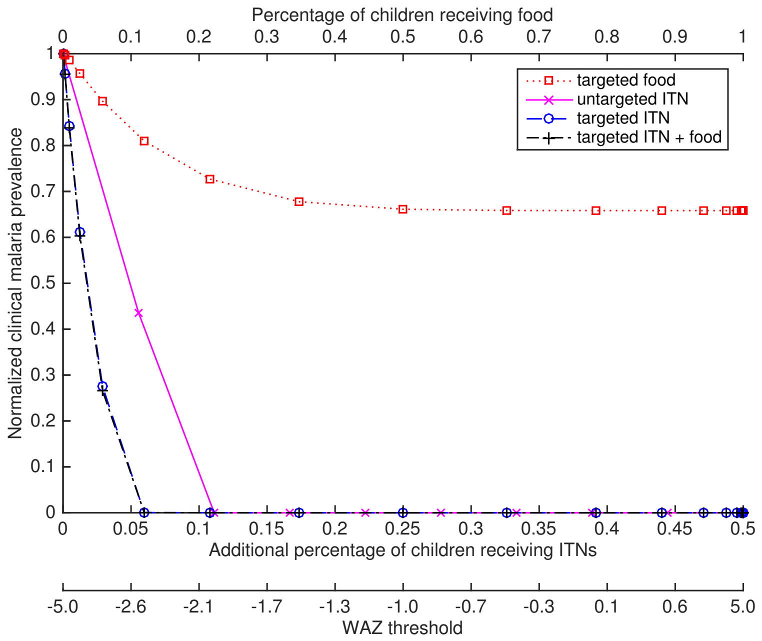

(a)

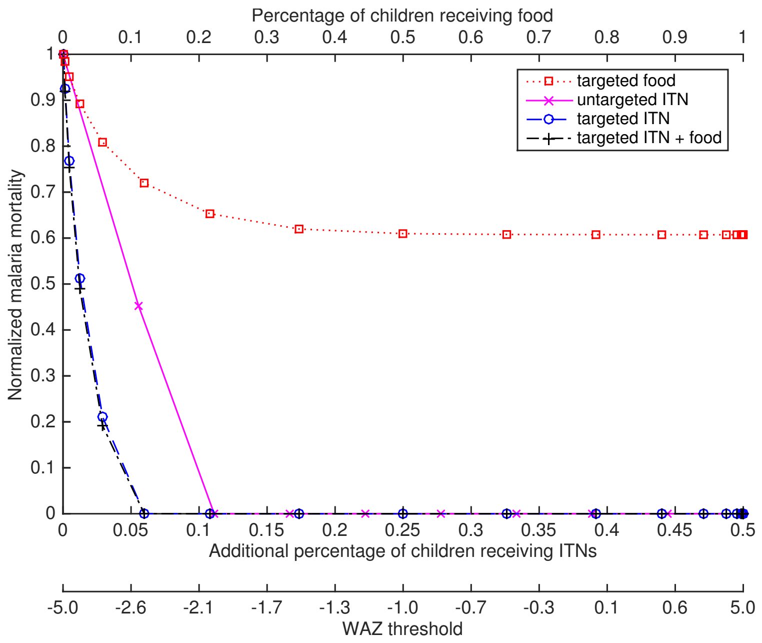

(b)

Figure 9: For the case of $k_1 = 0.153$, EIR $= 1$ (hypoendemic) and 50% baseline ITN coverage of children, which generates a clinical malaria prevalence of 0.010 in children at baseline, **(a)** the proportion of children with clinical malaria for a given coverage of a given policy, divided by the proportion of children with clinical malaria in the intervention-free case, and **(b)** the proportion of children who die from malaria for a given coverage of a given policy, divided by the proportion of children who die from malaria in the intervention-free case. The bottom of both figures gives the WAZ threshold that corresponds to a given coverage for the targeted policies (e.g., for the targeted ITN policy, ITNs are given to children with WAZ values below the threshold).

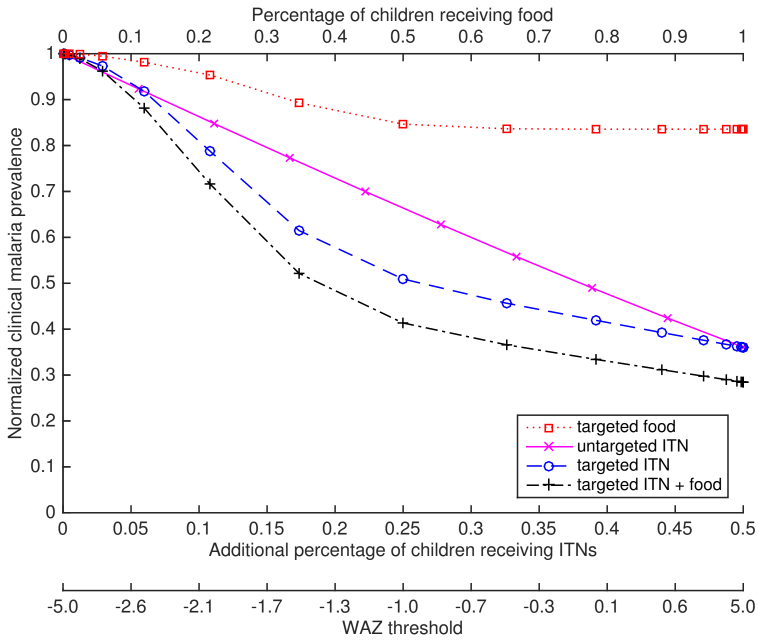

(a)

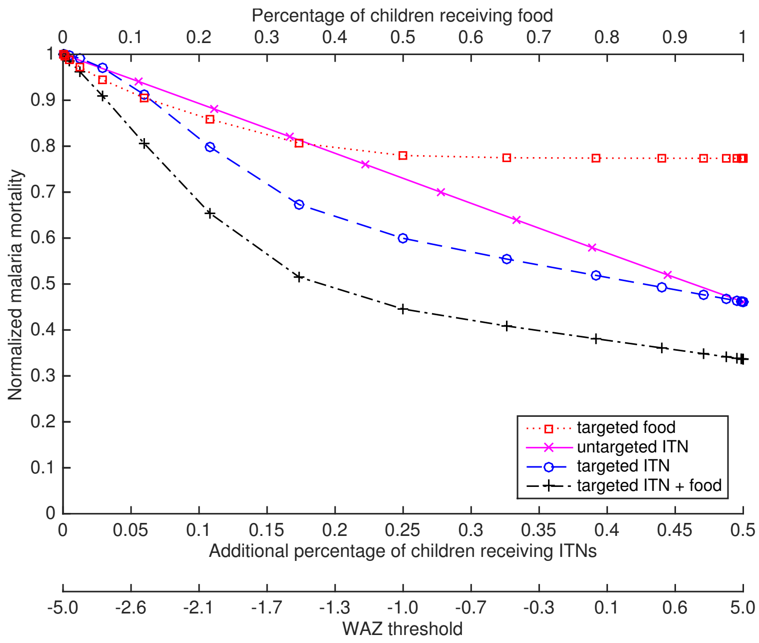

(b)

Figure 10: For the case of $k_1 = 0.153$, EIR $= 10$ (mesoendemic) and 50% baseline ITN coverage of children, which generates a clinical malaria prevalence of 0.250 in children at baseline, **(a)** the proportion of children with clinical malaria for a given coverage of a given policy, divided by the proportion of children with clinical malaria in the intervention-free case, and **(b)** the proportion of children who die from malaria for a given coverage of a given policy, divided by the proportion of children who die from malaria in the intervention-free case. The bottom of both figures gives the WAZ threshold that corresponds to a given coverage for the targeted policies (e.g., for the targeted ITN policy, ITNs are given to children with WAZ values below the threshold).

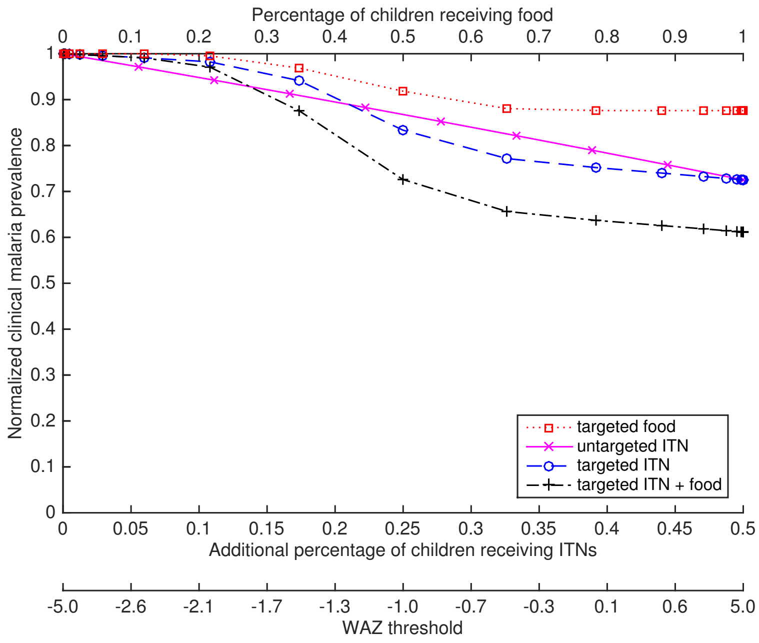

(a)

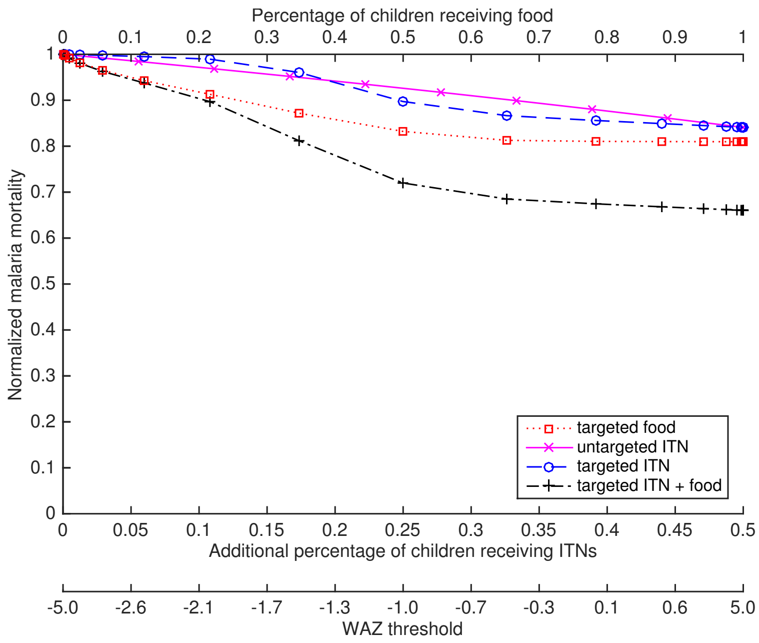

(b)

Figure 11: For the case of $k_1 = 0.153$, EIR $= 100$ (hyperendemic) and 50% baseline ITN coverage of children, which generates a clinical malaria prevalence of 0.505 in children at baseline, **(a)** the proportion of children with clinical malaria for a given coverage of a given policy, divided by the proportion of children with clinical malaria in the intervention-free case, and **(b)** the proportion of children who die from malaria for a given coverage of a given policy, divided by the proportion of children who die from malaria in the intervention-free case. The bottom of both figures gives the WAZ threshold that corresponds to a given coverage for the targeted policies (e.g., for the targeted ITN policy, ITNs are given to children with WAZ values below the threshold).

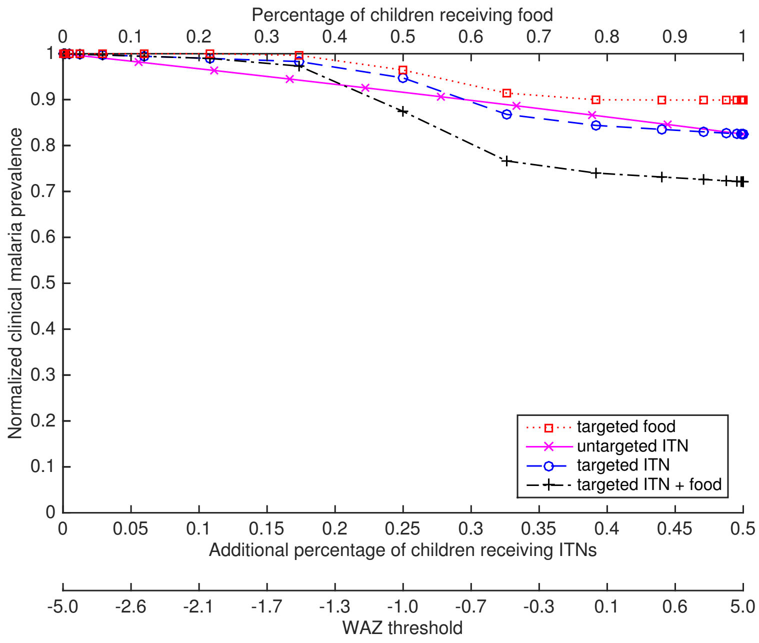

(a)

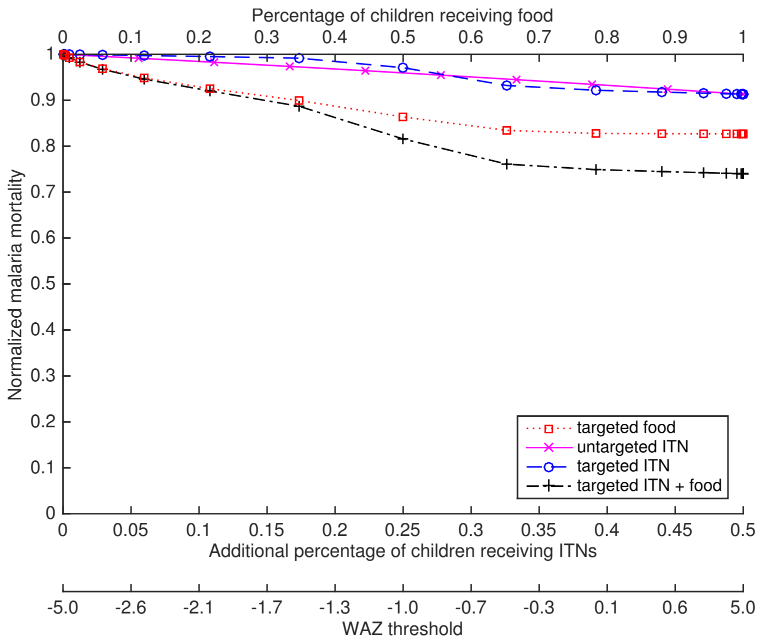

(b)

Figure 12: For the case of $k_1 = 0.153$, EIR $= 500$ (hyperendemic) and 50% baseline ITN coverage of children, which generates a clinical malaria prevalence of 0.621 in children at baseline, **(a)** the proportion of children with clinical malaria for a given coverage of a given policy, divided by the proportion of children with clinical malaria in the intervention-free case, and **(b)** the proportion of children who die from malaria for a given coverage of a given policy, divided by the proportion of children who die from malaria in the intervention-free case. The bottom of both figures gives the WAZ threshold that corresponds to a given coverage for the targeted policies (e.g., for the targeted ITN policy, ITNs are given to children with WAZ values below the threshold).

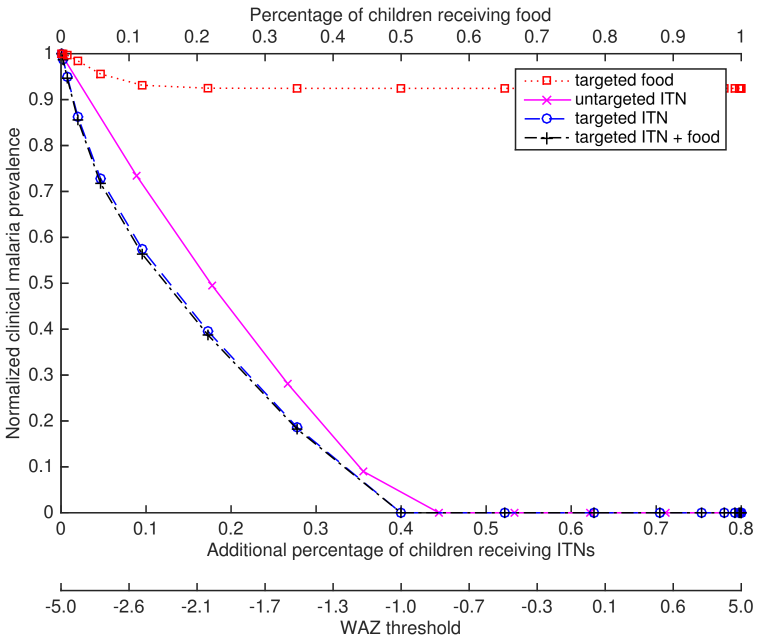

(a)

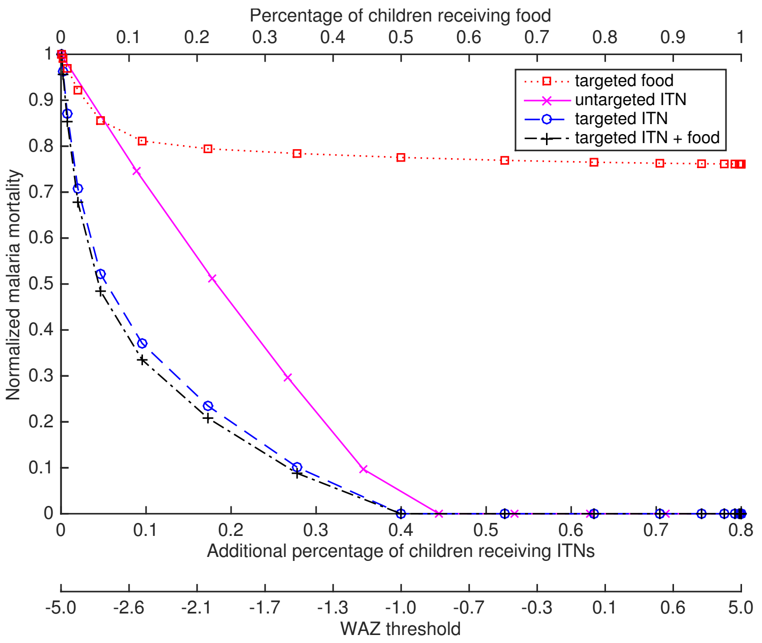

(b)

Figure 13: For the case of $k_1 = 0.04$, EIR $= 1$ (hypoendemic) and 20% baseline ITN coverage of children, which generates a clinical malaria prevalence of 0.049 in children at baseline, **(a)** the proportion of children with clinical malaria for a given coverage of a given policy, divided by the proportion of children with clinical malaria in the intervention-free case, and **(b)** the proportion of children who die from malaria for a given coverage of a given policy, divided by the proportion of children who die from malaria in the intervention-free case. The bottom of both figures gives the WAZ threshold that corresponds to a given coverage for the targeted policies (e.g., for the targeted ITN policy, ITNs are given to children with WAZ values below the threshold).

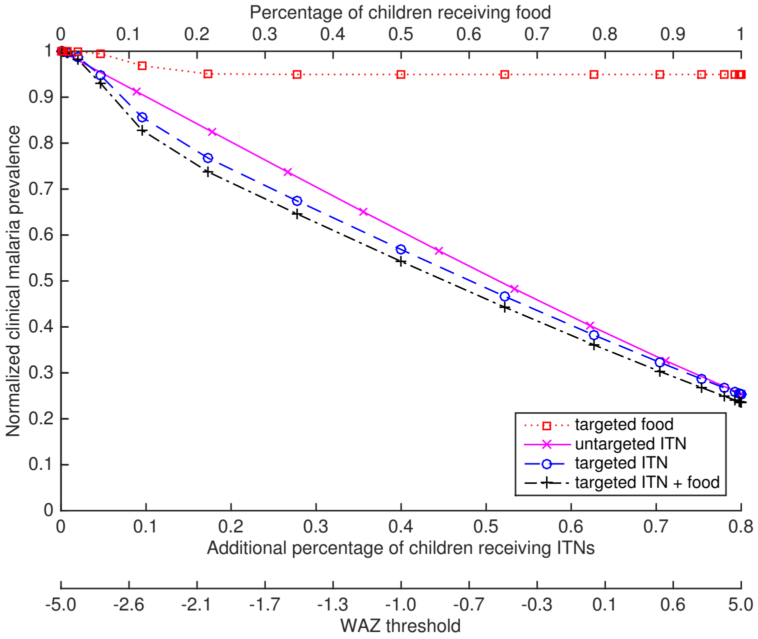

(a)

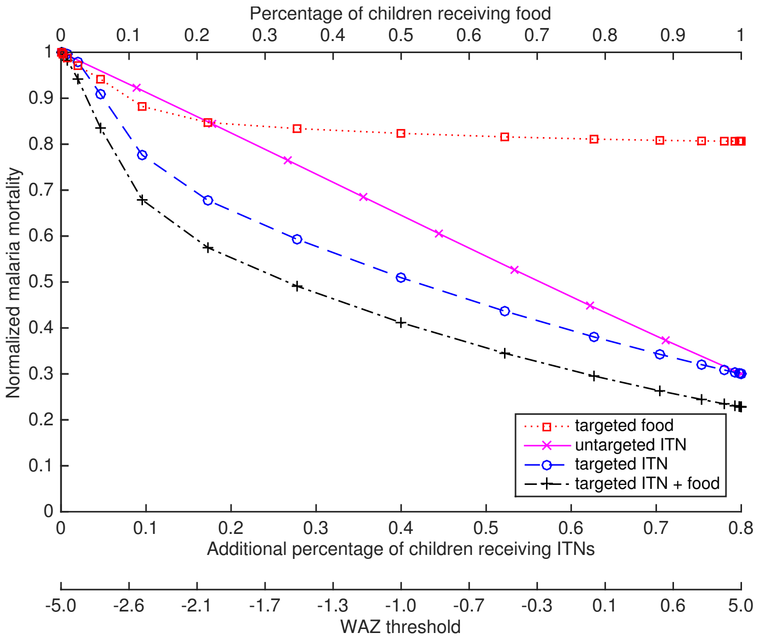

(b)

Figure 14: For the case of $k_1 = 0.04$, EIR $= 10$ (mesoendemic) and 20% baseline ITN coverage of children, which generates a clinical malaria prevalence of 0.354 in children at baseline, **(a)** the proportion of children with clinical malaria for a given coverage of a given policy, divided by the proportion of children with clinical malaria in the intervention-free case, and **(b)** the proportion of children who die from malaria for a given coverage of a given policy, divided by the proportion of children who die from malaria in the intervention-free case. The bottom of both figures gives the WAZ threshold that corresponds to a given coverage for the targeted policies (e.g., for the targeted ITN policy, ITNs are given to children with WAZ values below the threshold).

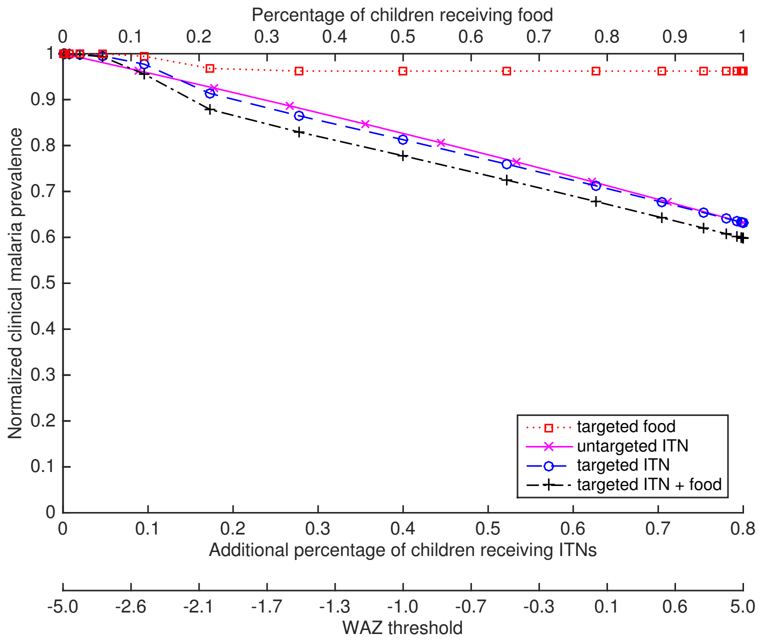

(a)

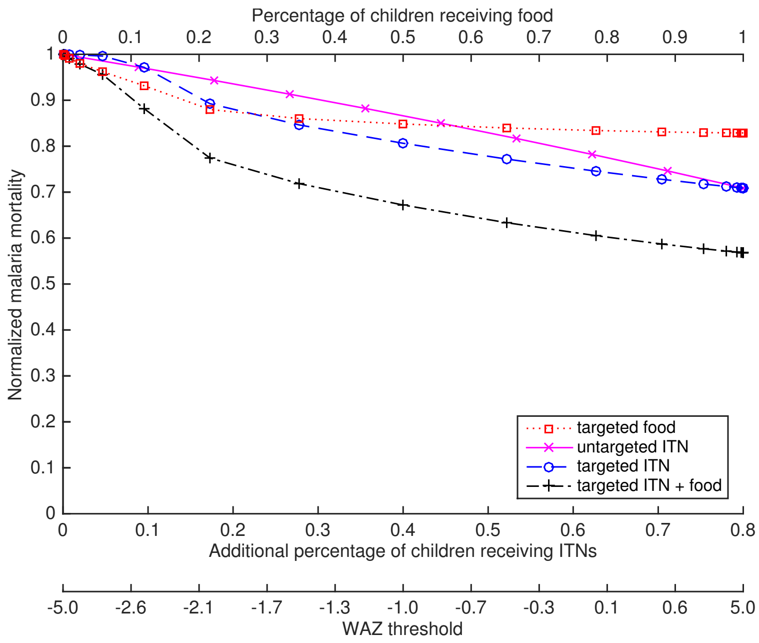

(b)

Figure 15: For the case of $k_1 = 0.04$, EIR $= 100$ (hyperendemic) and 20% baseline ITN coverage of children, which generates a clinical malaria prevalence of 0.580 in children at baseline, **(a)** the proportion of children with clinical malaria for a given coverage of a given policy, divided by the proportion of children with clinical malaria in the intervention-free case, and **(b)** the proportion of children who die from malaria for a given coverage of a given policy, divided by the proportion of children who die from malaria in the intervention-free case. The bottom of both figures gives the WAZ threshold that corresponds to a given coverage for the targeted policies (e.g., for the targeted ITN policy, ITNs are given to children with WAZ values below the threshold).

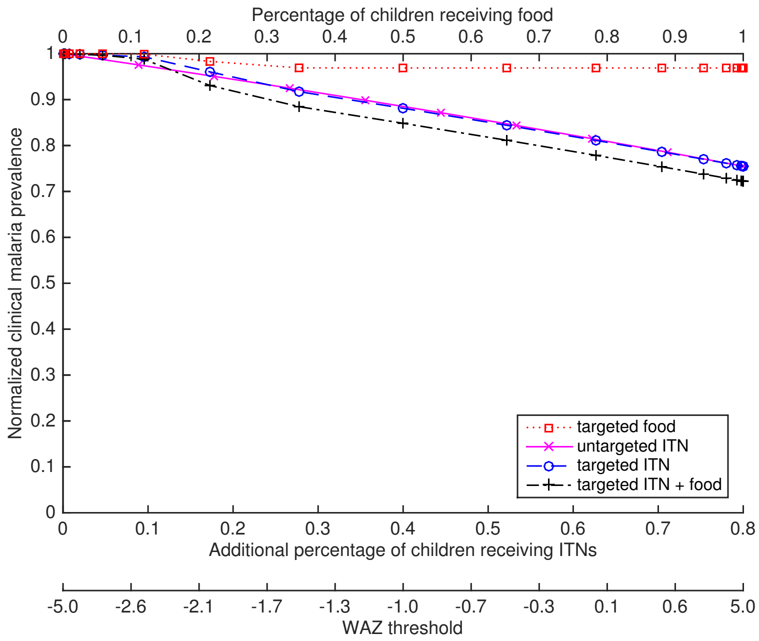

(a)

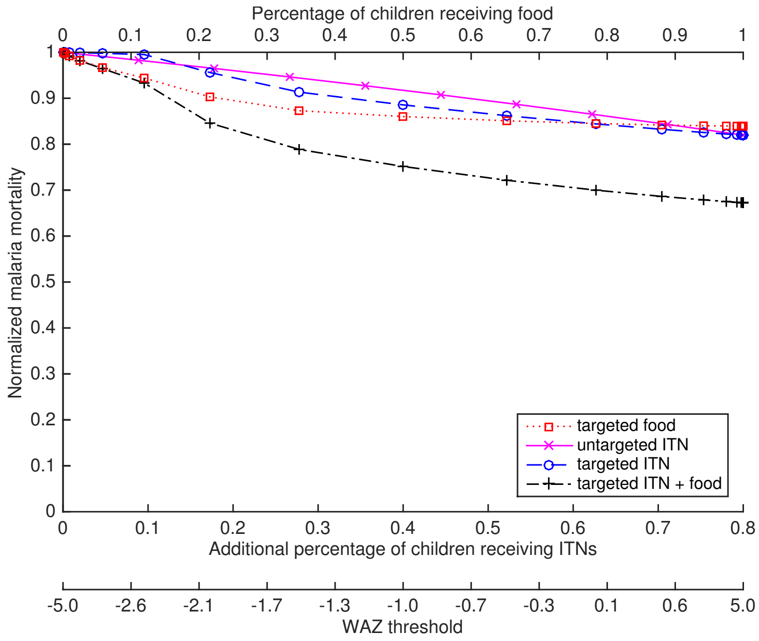

(b)

Figure 16: For the case of $k_1 = 0.04$, EIR $= 500$ (hyperendemic) and 20% baseline ITN coverage of children, which generates a clinical malaria prevalence of 0.680 in children at baseline, **(a)** the proportion of children with clinical malaria for a given coverage of a given policy, divided by the proportion of children with clinical malaria in the intervention-free case, and **(b)** the proportion of children who die from malaria for a given coverage of a given policy, divided by the proportion of children who die from malaria in the intervention-free case. The bottom of both figures gives the WAZ threshold that corresponds to a given coverage for the targeted policies (e.g., for the targeted ITN policy, ITNs are given to children with WAZ values below the threshold).

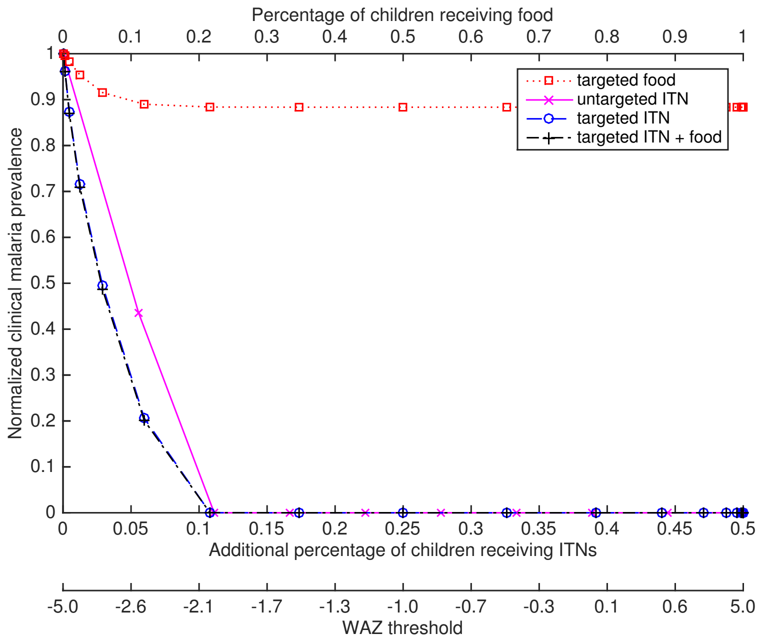

(a)

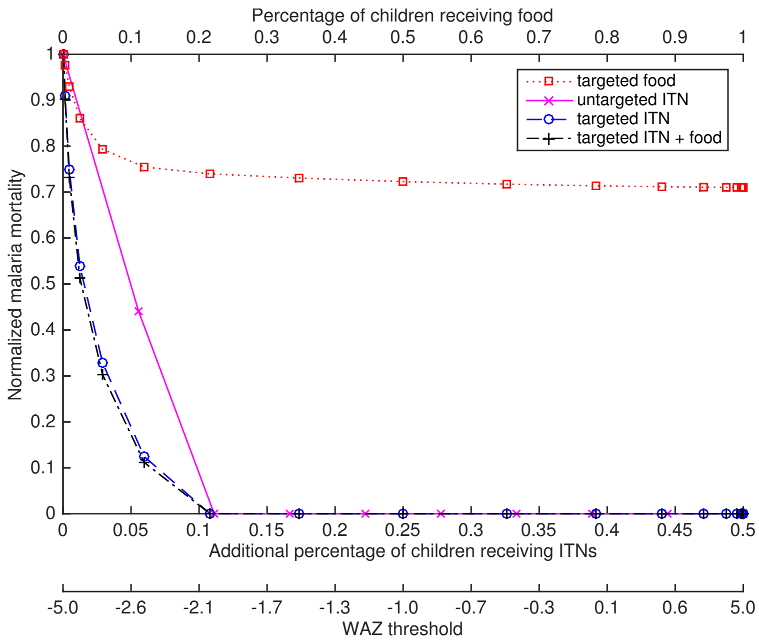

(b)

Figure 17: For the case of $k_1 = 0.04$, EIR $= 1$ (hypoendemic) and 50% baseline ITN coverage of children, which generates a clinical malaria prevalence of 0.010 in children at baseline, **(a)** the proportion of children with clinical malaria for a given coverage of a given policy, divided by the proportion of children with clinical malaria in the intervention-free case, and **(b)** the proportion of children who die from malaria for a given coverage of a given policy, divided by the proportion of children who die from malaria in the intervention-free case. The bottom of both figures gives the WAZ threshold that corresponds to a given coverage for the targeted policies (e.g., for the targeted ITN policy, ITNs are given to children with WAZ values below the threshold).

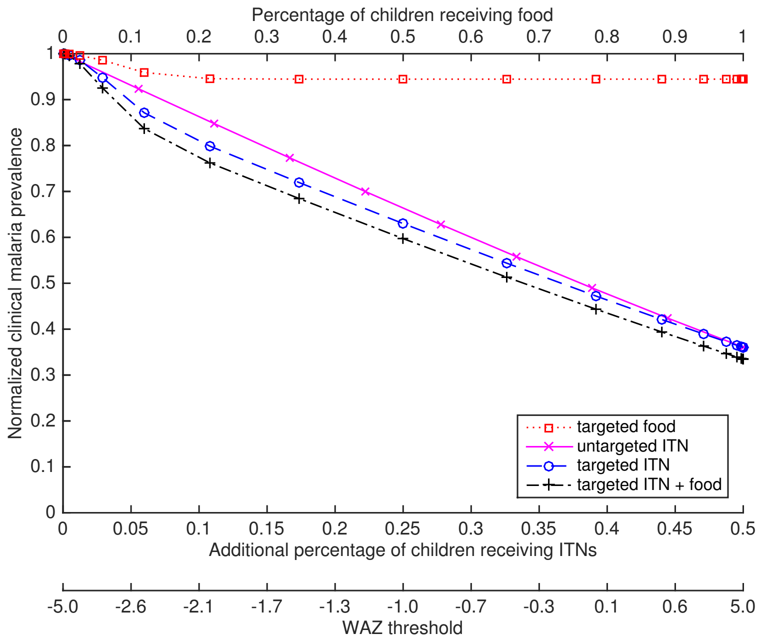

(a)

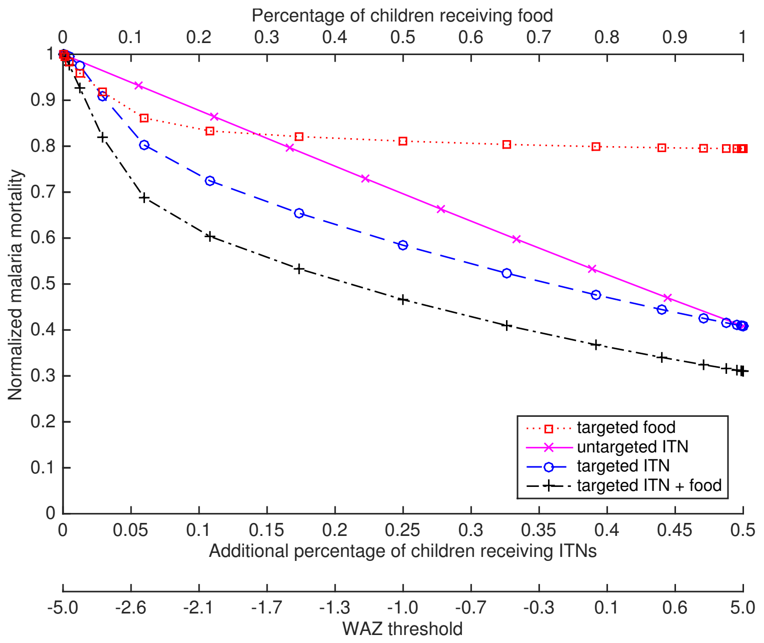

(b)

Figure 18: For the case of $k_1 = 0.04$, EIR $= 10$ (mesoendemic) and 50% baseline ITN coverage of children, which generates a clinical malaria prevalence of 0.250 in children at baseline, **(a)** the proportion of children with clinical malaria for a given coverage of a given policy, divided by the proportion of children with clinical malaria in the intervention-free case, and **(b)** the proportion of children who die from malaria for a given coverage of a given policy, divided by the proportion of children who die from malaria in the intervention-free case. The bottom of both figures gives the WAZ threshold that corresponds to a given coverage for the targeted policies (e.g., for the targeted ITN policy, ITNs are given to children with WAZ values below the threshold).

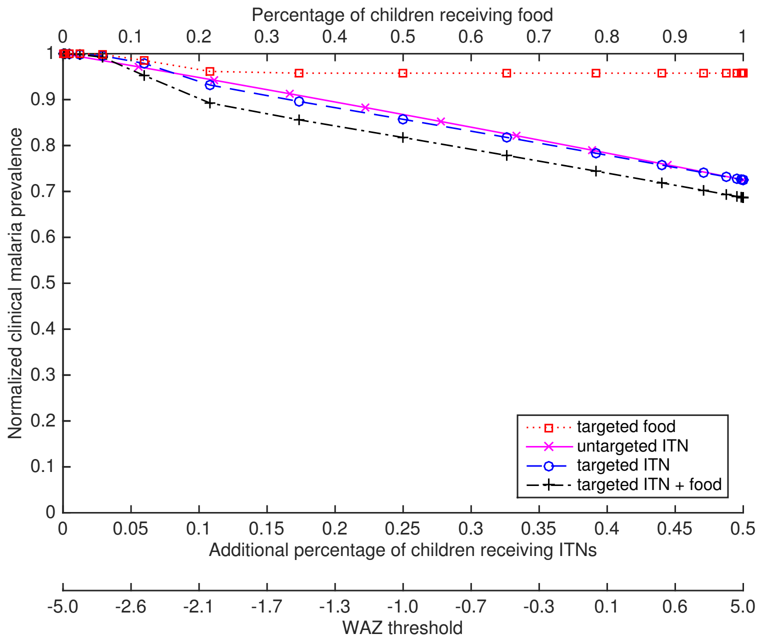

(a)

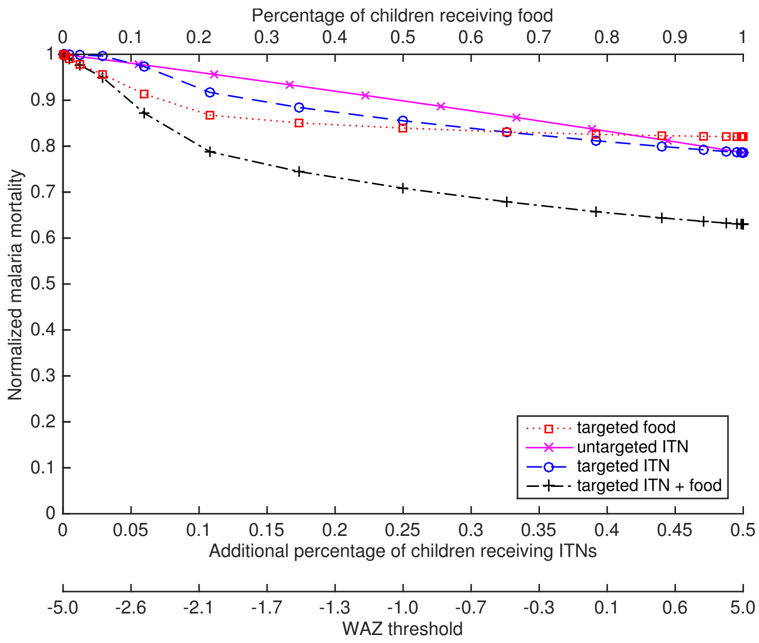

(b)

Figure 19: For the case of $k_1 = 0.04$, EIR $= 100$ (hyperendemic) and 50% baseline ITN coverage of children, which generates a clinical malaria prevalence of 0.505 in children at baseline, **(a)** the proportion of children with clinical malaria for a given coverage of a given policy, divided by the proportion of children with clinical malaria in the intervention-free case, and **(b)** the proportion of children who die from malaria for a given coverage of a given policy, divided by the proportion of children who die from malaria in the intervention-free case. The bottom of both figures gives the WAZ threshold that corresponds to a given coverage for the targeted policies (e.g., for the targeted ITN policy, ITNs are given to children with WAZ values below the threshold).

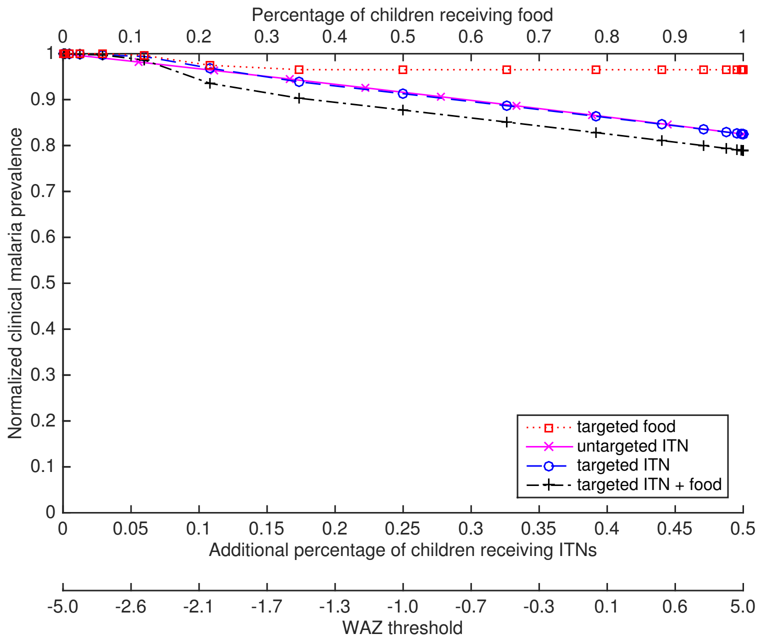

(a)

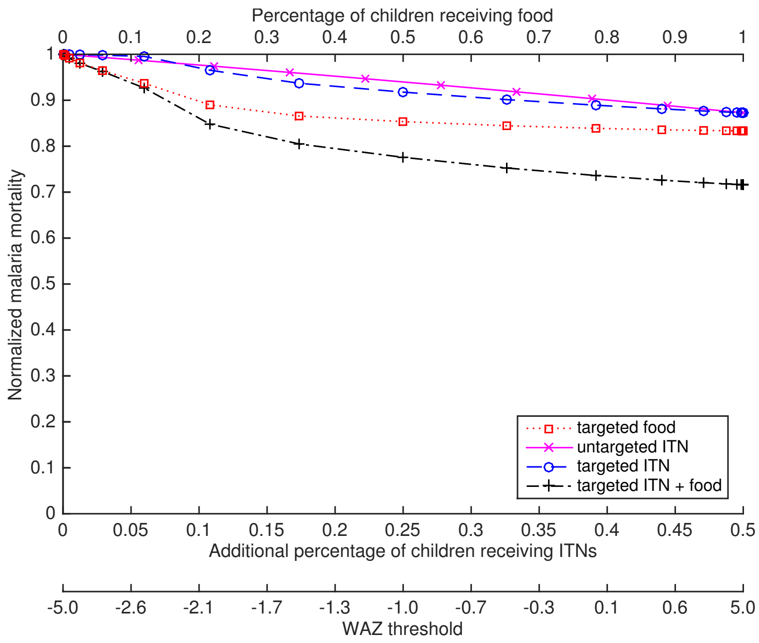

(b)

Figure 20: For the case of $k_1 = 0.04$, EIR $= 500$ (hyperendemic) and 50% baseline ITN coverage of children, which generates a clinical malaria prevalence of 0.621 in children at baseline, **(a)** the proportion of children with clinical malaria for a given coverage of a given policy, divided by the proportion of children with clinical malaria in the intervention-free case, and **(b)** the proportion of children who die from malaria for a given coverage of a given policy, divided by the proportion of children who die from malaria in the intervention-free case. The bottom of both figures gives the WAZ threshold that corresponds to a given coverage for the targeted policies (e.g., for the targeted ITN policy, ITNs are given to children with WAZ values below the threshold).

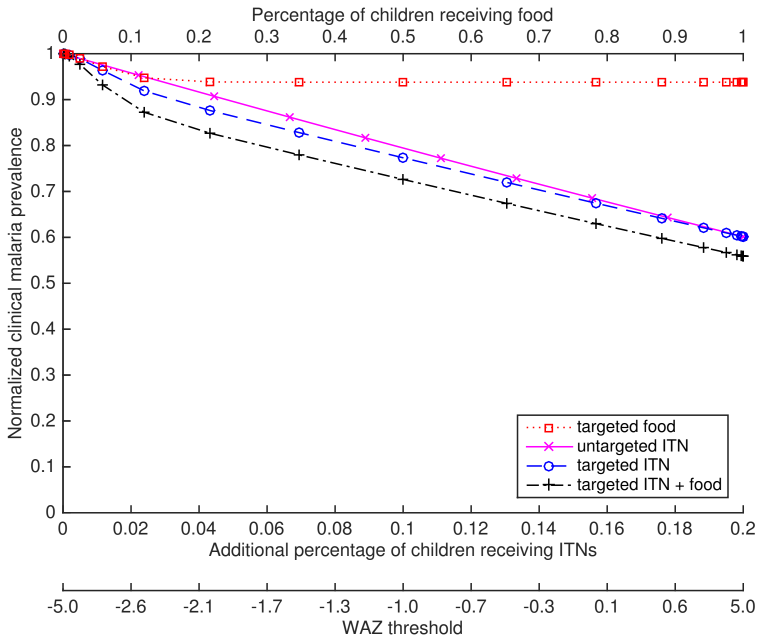

(a)

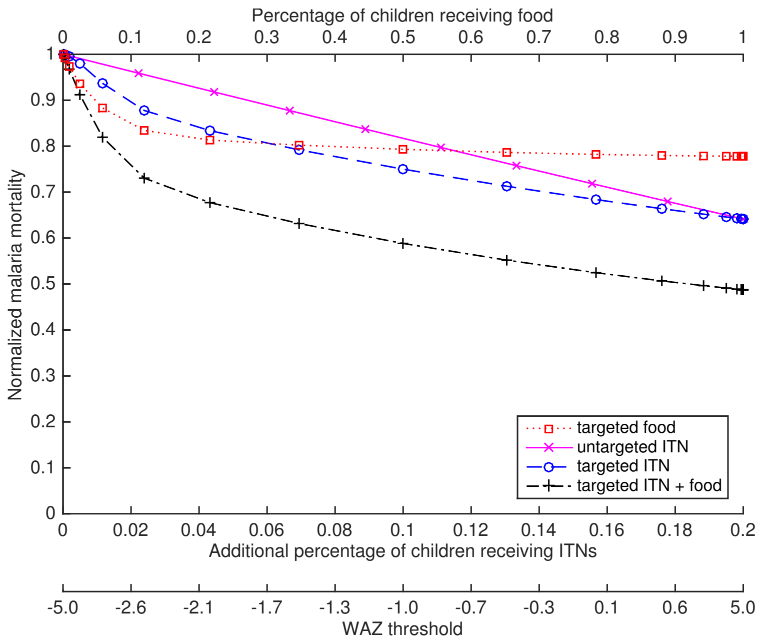

(b)

Figure 21: For the case of $k_1 = 0.04$, EIR $= 10$ (mesoendemic) and 80% baseline ITN coverage of children, which generates a clinical malaria prevalence of 0.149 in children at baseline, **(a)** the proportion of children with clinical malaria for a given coverage of a given policy, divided by the proportion of children with clinical malaria in the intervention-free case, and **(b)** the proportion of children who die from malaria for a given coverage of a given policy, divided by the proportion of children who die from malaria in the intervention-free case. The bottom of both figures gives the WAZ threshold that corresponds to a given coverage for the targeted policies (e.g., for the targeted ITN policy, ITNs are given to children with WAZ values below the threshold).

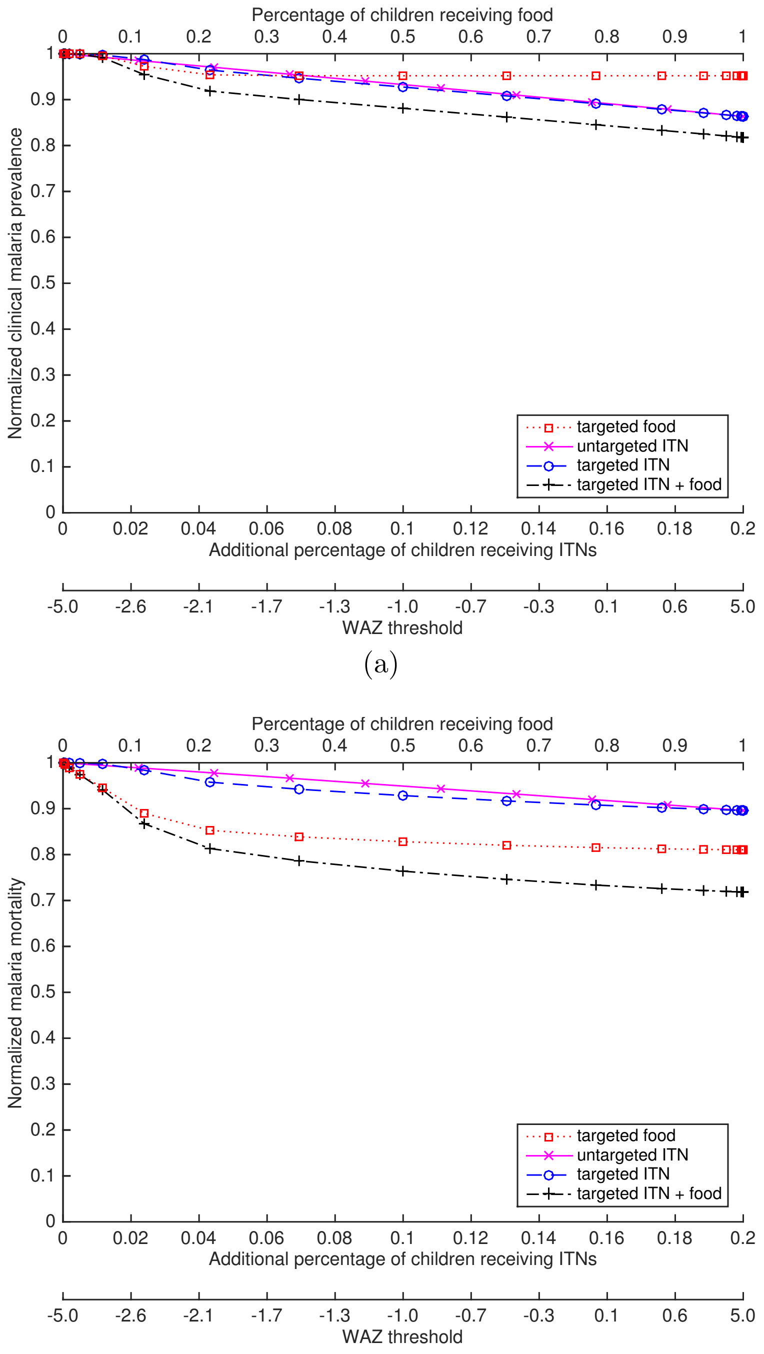

(a)

(b)

Figure 22: For the case of $k_1 = 0.04$, EIR $= 100$ (hyperendemic) and 80% baseline ITN coverage of children, which generates a clinical malaria prevalence of 0.424 in children at baseline, **(a)** the proportion of children with clinical malaria for a given coverage of a given policy, divided by the proportion of children with clinical malaria in the intervention-free case, and **(b)** the proportion of children who die from malaria for a given coverage of a given policy, divided by the proportion of children who die from malaria in the intervention-free case. The bottom of both figures gives the WAZ threshold that corresponds to a given coverage for the targeted policies (e.g., for the targeted ITN policy, ITNs are given to children with WAZ values below the threshold).

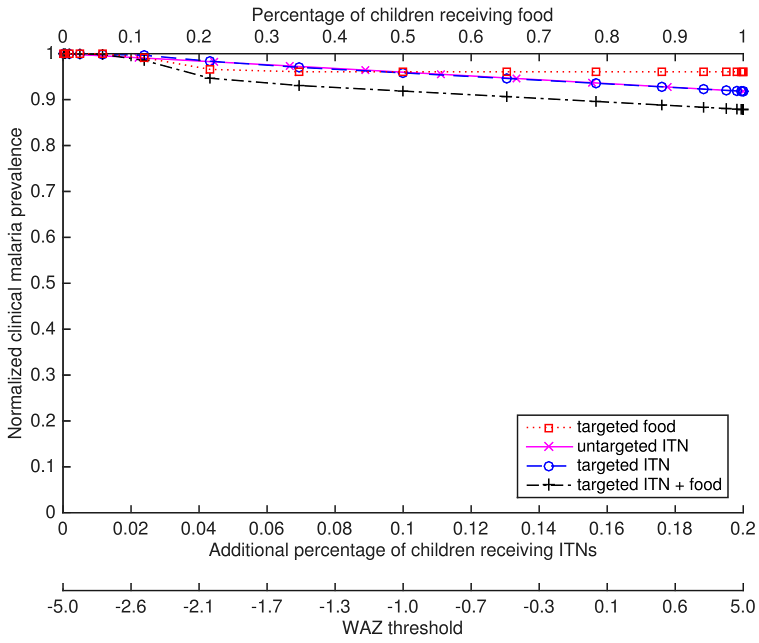

(a)

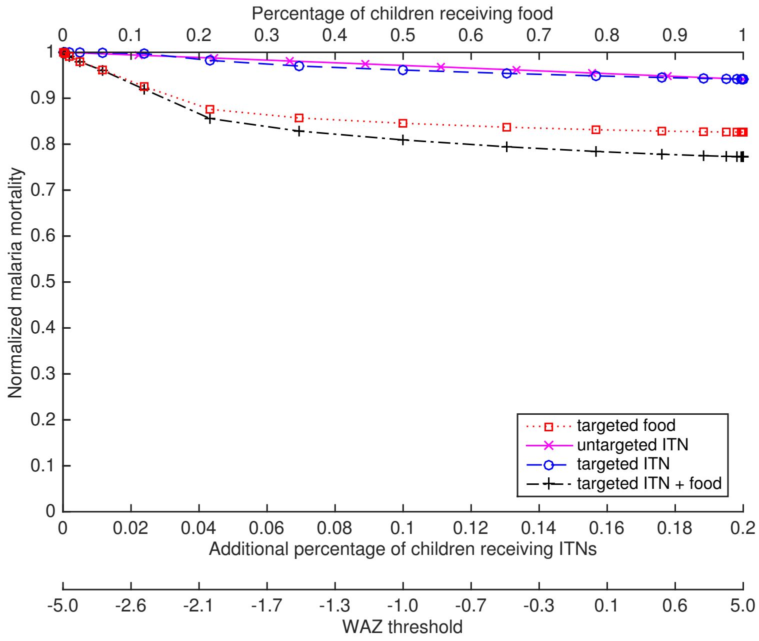

(b)

Figure 23: For the case of $k_1 = 0.04$, EIR $= 500$ (hyperendemic) and 80% baseline ITN coverage of children, which generates a clinical malaria prevalence of 0.558 in children at baseline, **(a)** the proportion of children with clinical malaria for a given coverage of a given policy, divided by the proportion of children with clinical malaria in the intervention-free case, and **(b)** the proportion of children who die from malaria for a given coverage of a given policy, divided by the proportion of children who die from malaria in the intervention-free case. The bottom of both figures gives the WAZ threshold that corresponds to a given coverage for the targeted policies (e.g., for the targeted ITN policy, ITNs are given to children with WAZ values below the threshold).

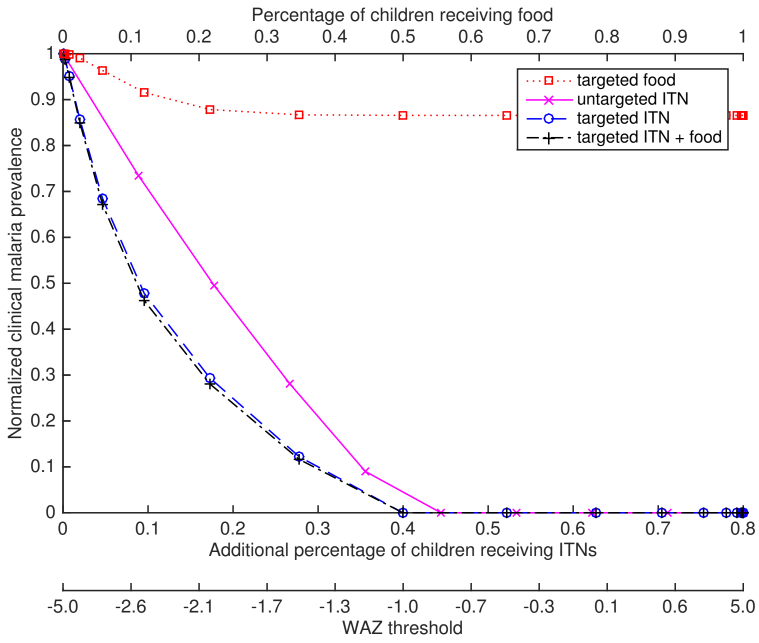

(a)

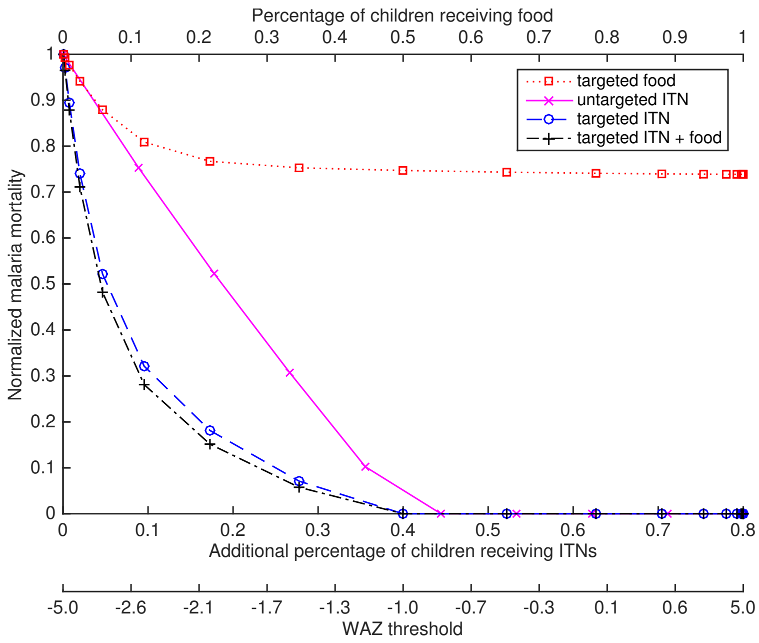

(b)

Figure 24: For the case of $k_1 = 0.08$, EIR $= 1$ (hypoendemic) and 20% baseline ITN coverage of children, which generates a clinical malaria prevalence of 0.049 in children at baseline, **(a)** the proportion of children with clinical malaria for a given coverage of a given policy, divided by the proportion of children with clinical malaria in the intervention-free case, and **(b)** the proportion of children who die from malaria for a given coverage of a given policy, divided by the proportion of children who die from malaria in the intervention-free case. The bottom of both figures gives the WAZ threshold that corresponds to a given coverage for the targeted policies (e.g., for the targeted ITN policy, ITNs are given to children with WAZ values below the threshold).

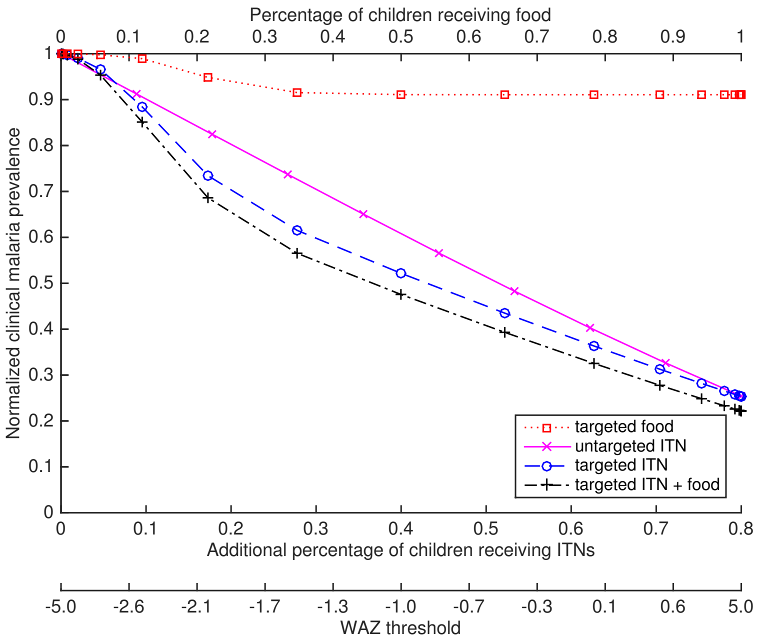

(a)

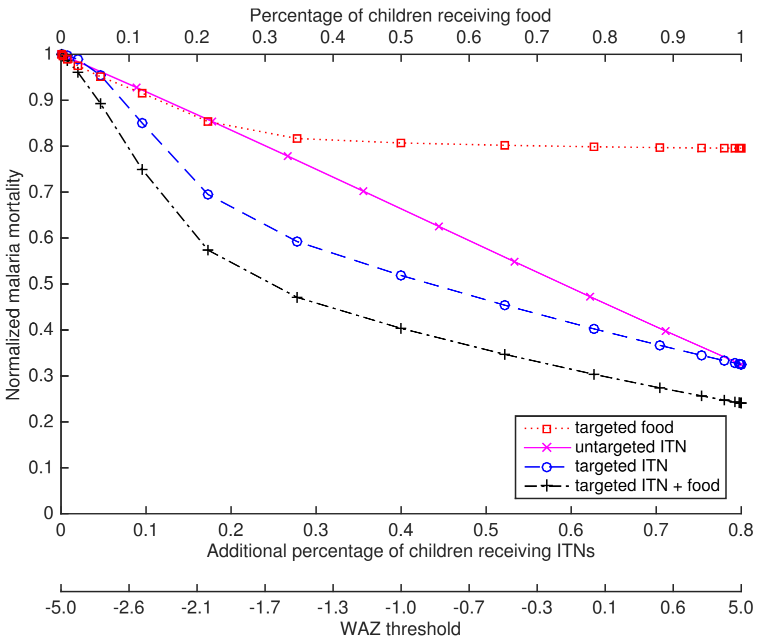

(b)

Figure 25: For the case of $k_1 = 0.08$, $\text{EIR} = 10$ (mesoendemic) and 20% baseline ITN coverage of children, which generates a clinical malaria prevalence of 0.354 in children at baseline, **(a)** the proportion of children with clinical malaria for a given coverage of a given policy, divided by the proportion of children with clinical malaria in the intervention-free case, and **(b)** the proportion of children who die from malaria for a given coverage of a given policy, divided by the proportion of children who die from malaria in the intervention-free case. The bottom of both figures gives the WAZ threshold that corresponds to a given coverage for the targeted policies (e.g., for the targeted ITN policy, ITNs are given to children with WAZ values below the threshold).

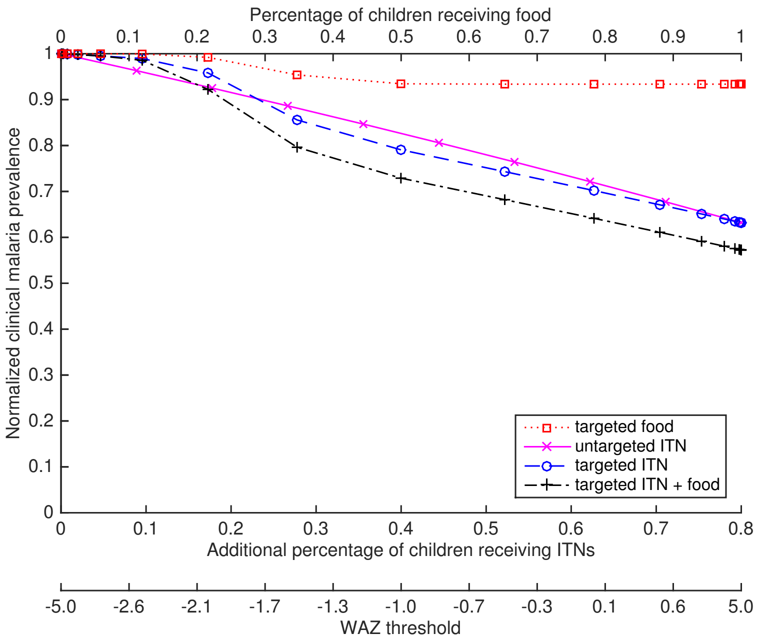

(a)

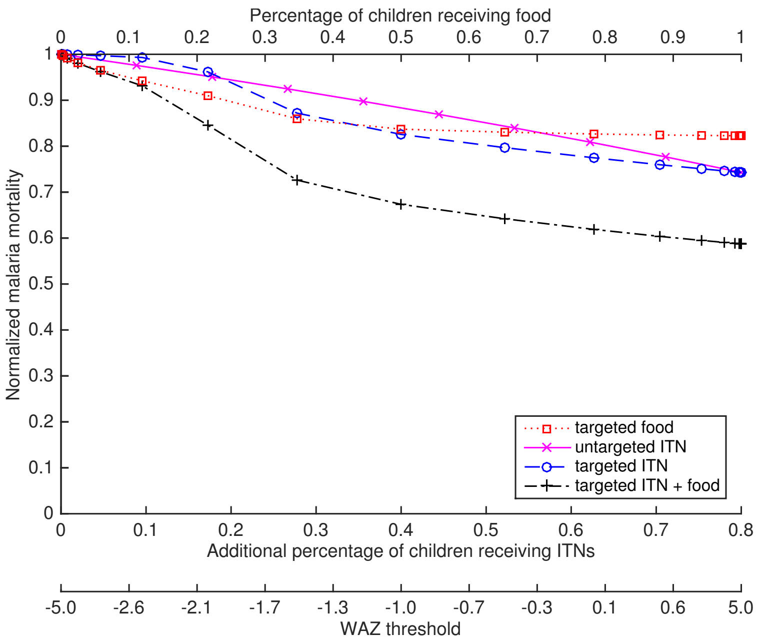

(b)

Figure 26: For the case of $k_1 = 0.08$, EIR $= 100$ (hyperendemic) and 20% baseline ITN coverage of children, which generates a clinical malaria prevalence of 0.580 in children at baseline, **(a)** the proportion of children with clinical malaria for a given coverage of a given policy, divided by the proportion of children with clinical malaria in the intervention-free case, and **(b)** the proportion of children who die from malaria for a given coverage of a given policy, divided by the proportion of children who die from malaria in the intervention-free case. The bottom of both figures gives the WAZ threshold that corresponds to a given coverage for the targeted policies (e.g., for the targeted ITN policy, ITNs are given to children with WAZ values below the threshold).

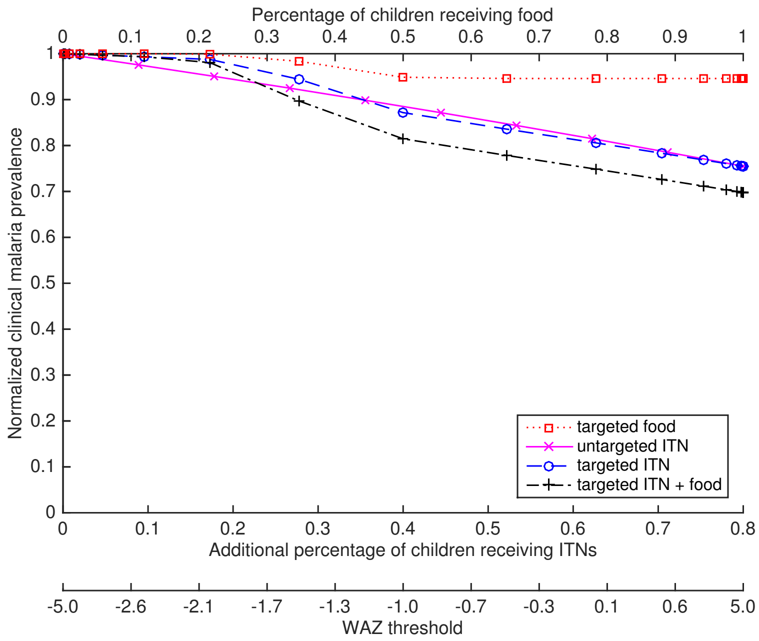

(a)

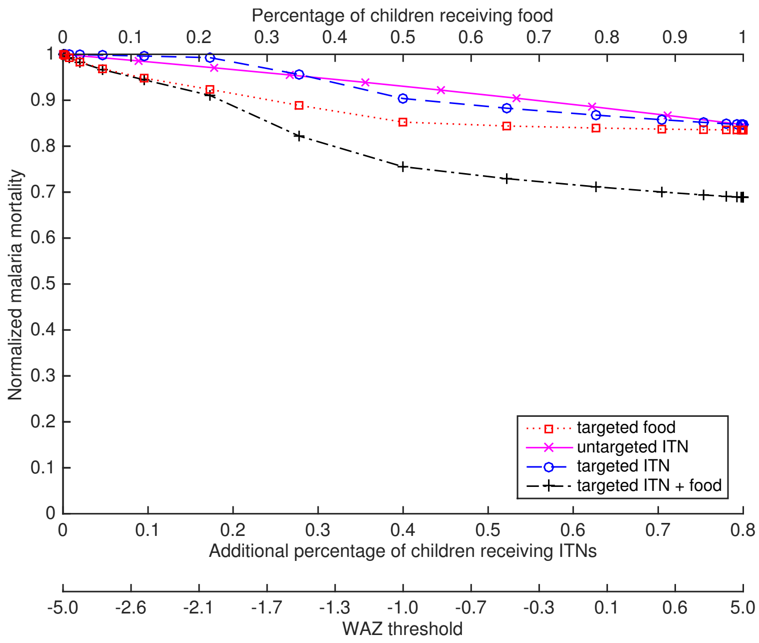

(b)

Figure 27: For the case of $k_1 = 0.08$, EIR $= 500$ (hyperendemic) and 20% baseline ITN coverage of children, which generates a clinical malaria prevalence of 0.680 in children at baseline, **(a)** the proportion of children with clinical malaria for a given coverage of a given policy, divided by the proportion of children with clinical malaria in the intervention-free case, and **(b)** the proportion of children who die from malaria for a given coverage of a given policy, divided by the proportion of children who die from malaria in the intervention-free case. The bottom of both figures gives the WAZ threshold that corresponds to a given coverage for the targeted policies (e.g., for the targeted ITN policy, ITNs are given to children with WAZ values below the threshold).

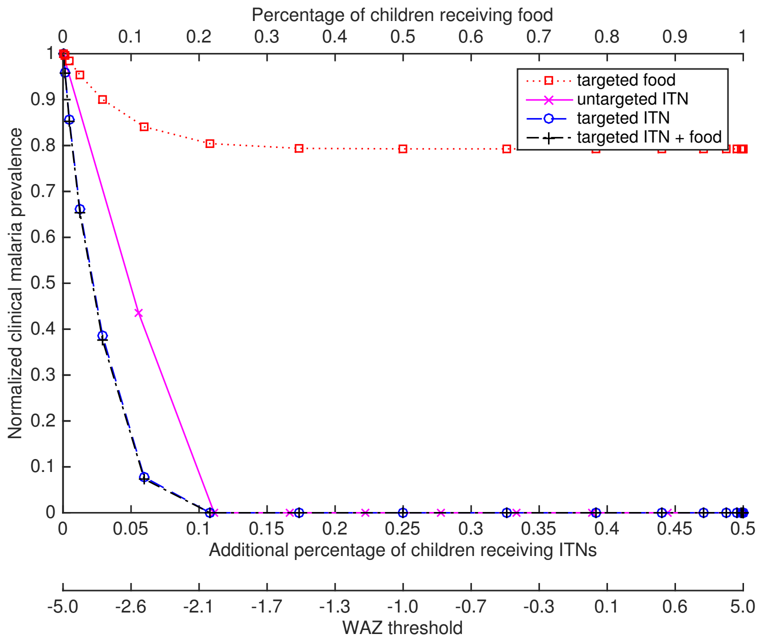

(a)

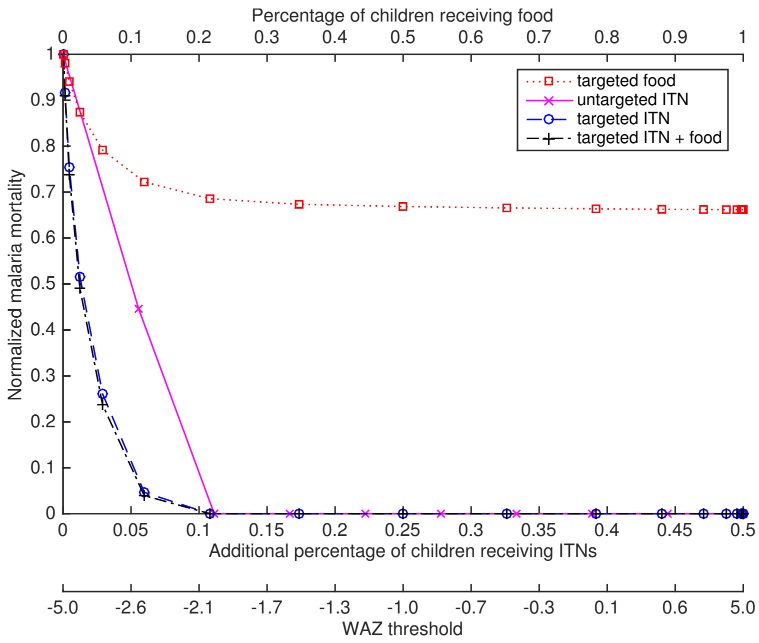

(b)

Figure 28: For the case of $k_1 = 0.08$, $\text{EIR} = 1$ (hypoendemic) and 50% baseline ITN coverage of children, which generates a clinical malaria prevalence of 0.010 in children at baseline, **(a)** the proportion of children with clinical malaria for a given coverage of a given policy, divided by the proportion of children with clinical malaria in the intervention-free case, and **(b)** the proportion of children who die from malaria for a given coverage of a given policy, divided by the proportion of children who die from malaria in the intervention-free case. The bottom of both figures gives the WAZ threshold that corresponds to a given coverage for the targeted policies (e.g., for the targeted ITN policy, ITNs are given to children with WAZ values below the threshold).

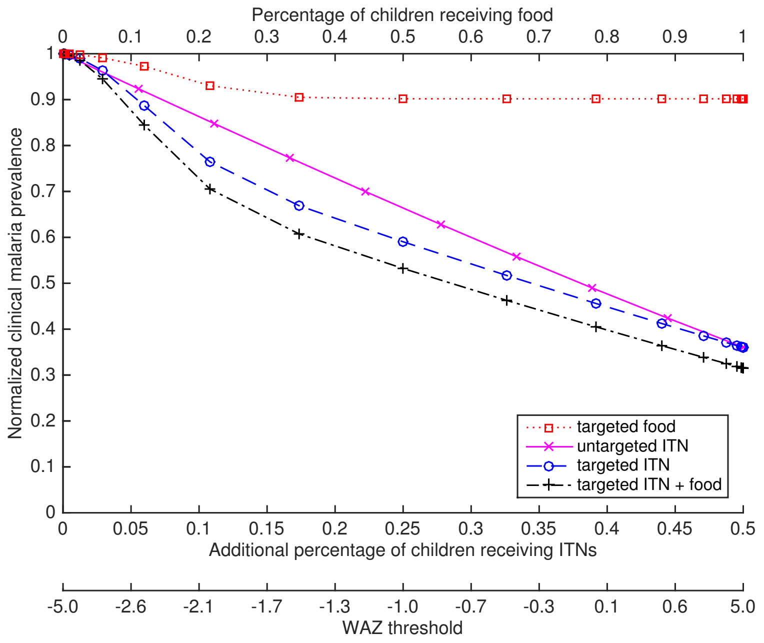

(a)

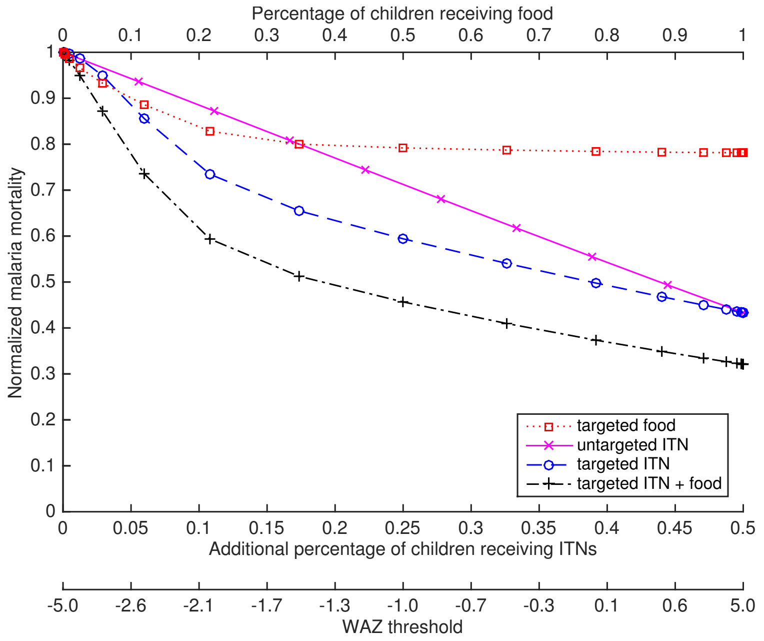

(b)

Figure 29: For the case of $k_1 = 0.08$, EIR $= 10$ (mesoendemic) and 50% baseline ITN coverage of children, which generates a clinical malaria prevalence of 0.250 in children at baseline, **(a)** the proportion of children with clinical malaria for a given coverage of a given policy, divided by the proportion of children with clinical malaria in the intervention-free case, and **(b)** the proportion of children who die from malaria for a given coverage of a given policy, divided by the proportion of children who die from malaria in the intervention-free case. The bottom of both figures gives the WAZ threshold that corresponds to a given coverage for the targeted policies (e.g., for the targeted ITN policy, ITNs are given to children with WAZ values below the threshold).

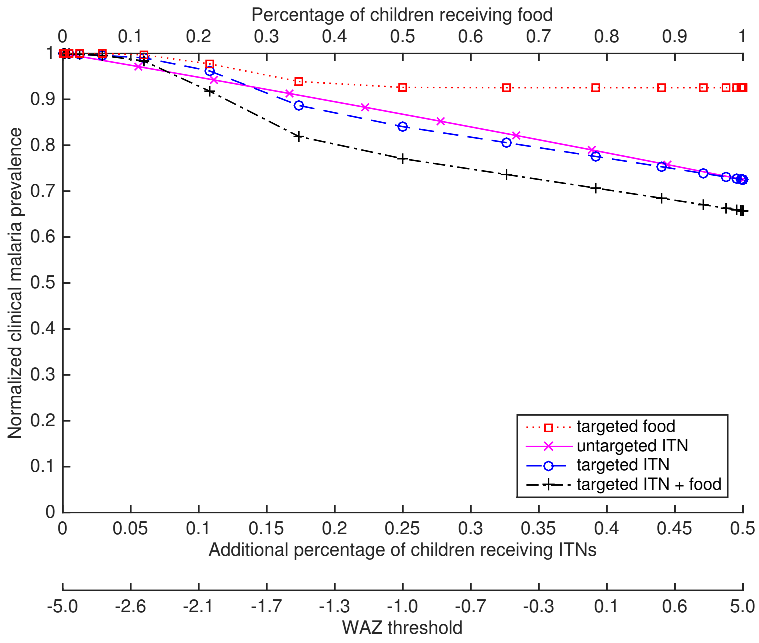

(a)

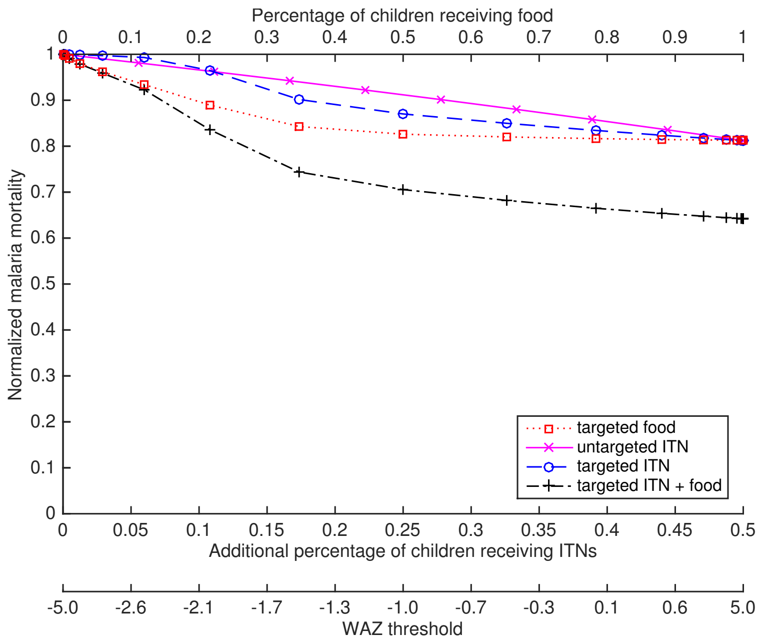

(b)

Figure 30: For the case of $k_1 = 0.08$, EIR = 100 (hyperendemic) and 50% baseline ITN coverage of children, which generates a clinical malaria prevalence of 0.505 in children at baseline, **(a)** the proportion of children with clinical malaria for a given coverage of a given policy, divided by the proportion of children with clinical malaria in the intervention-free case, and **(b)** the proportion of children who die from malaria for a given coverage of a given policy, divided by the proportion of children who die from malaria in the intervention-free case. The bottom of both figures gives the WAZ threshold that corresponds to a given coverage for the targeted policies (e.g., for the targeted ITN policy, ITNs are given to children with WAZ values below the threshold).

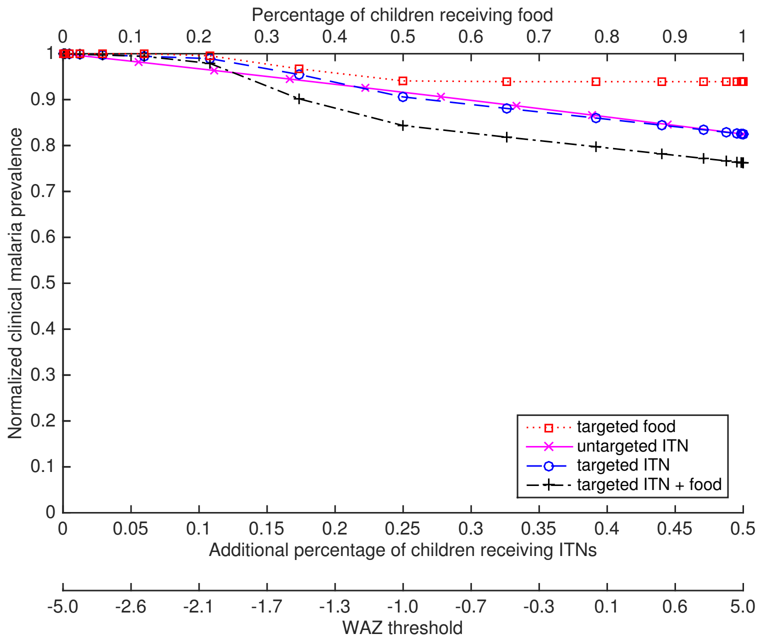

(a)

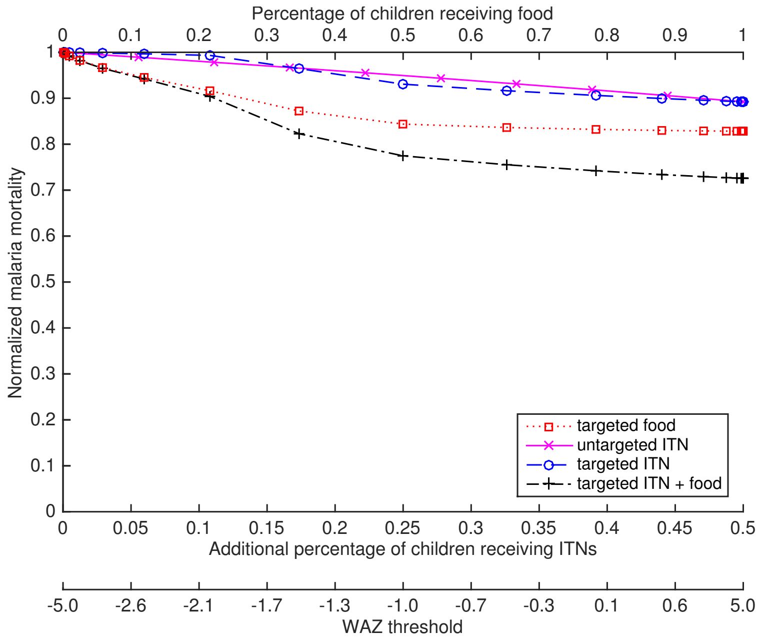

(b)

Figure 31: For the case of $k_1 = 0.08$, EIR $= 500$ (hyperendemic) and 50% baseline ITN coverage of children, which generates a clinical malaria prevalence of 0.621 in children at baseline, **(a)** the proportion of children with clinical malaria for a given coverage of a given policy, divided by the proportion of children with clinical malaria in the intervention-free case, and **(b)** the proportion of children who die from malaria for a given coverage of a given policy, divided by the proportion of children who die from malaria in the intervention-free case. The bottom of both figures gives the WAZ threshold that corresponds to a given coverage for the targeted policies (e.g., for the targeted ITN policy, ITNs are given to children with WAZ values below the threshold).

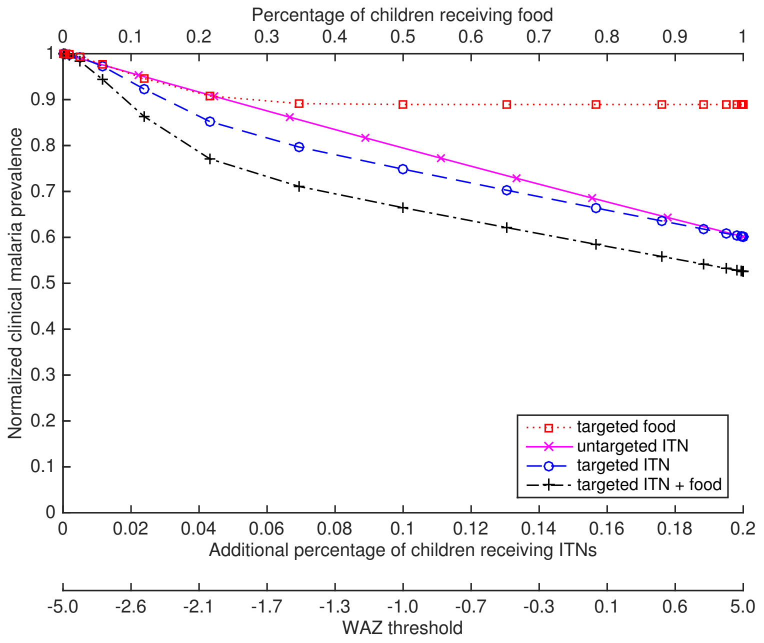

(a)

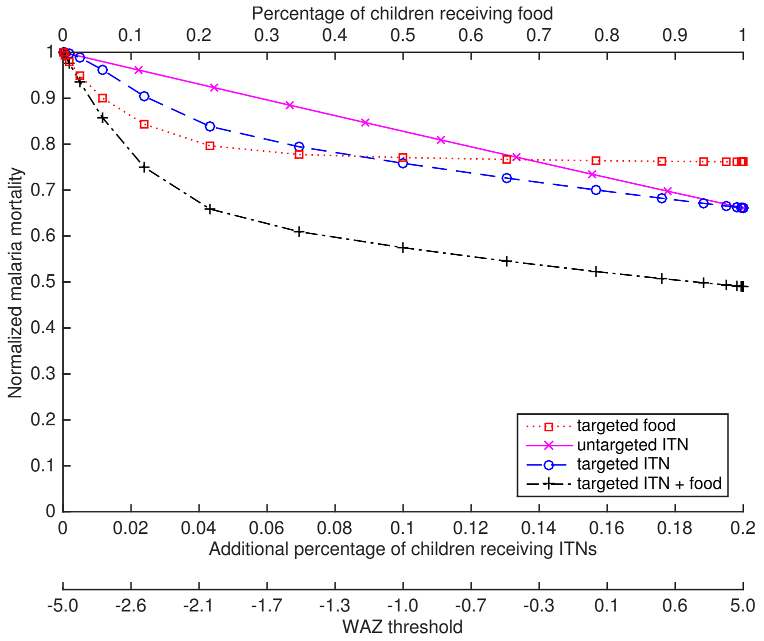

(b)

Figure 32: For the case of $k_1 = 0.08$, EIR $= 10$ (mesoendemic) and 80% baseline ITN coverage of children, which generates a clinical malaria prevalence of 0.149 in children at baseline, **(a)** the proportion of children with clinical malaria for a given coverage of a given policy, divided by the proportion of children with clinical malaria in the intervention-free case, and **(b)** the proportion of children who die from malaria for a given coverage of a given policy, divided by the proportion of children who die from malaria in the intervention-free case. The bottom of both figures gives the WAZ threshold that corresponds to a given coverage for the targeted policies (e.g., for the targeted ITN policy, ITNs are given to children with WAZ values below the threshold).

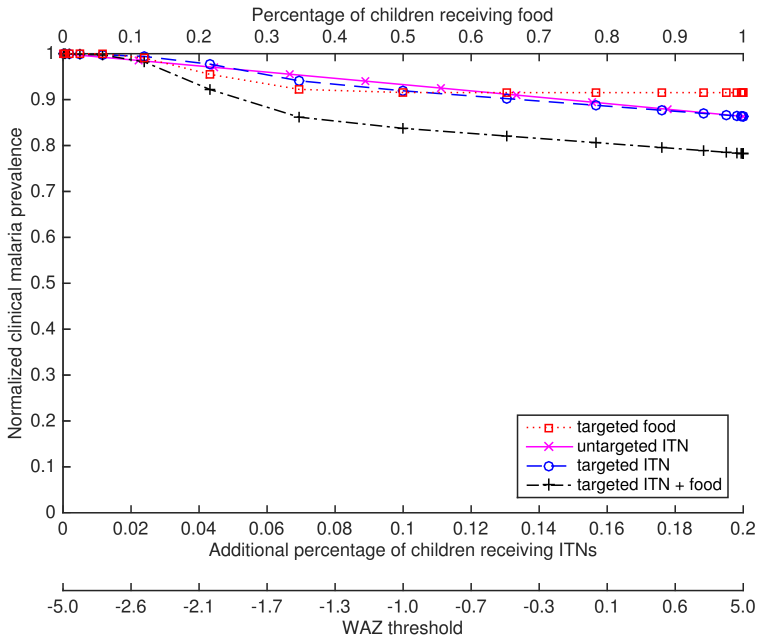

(a)

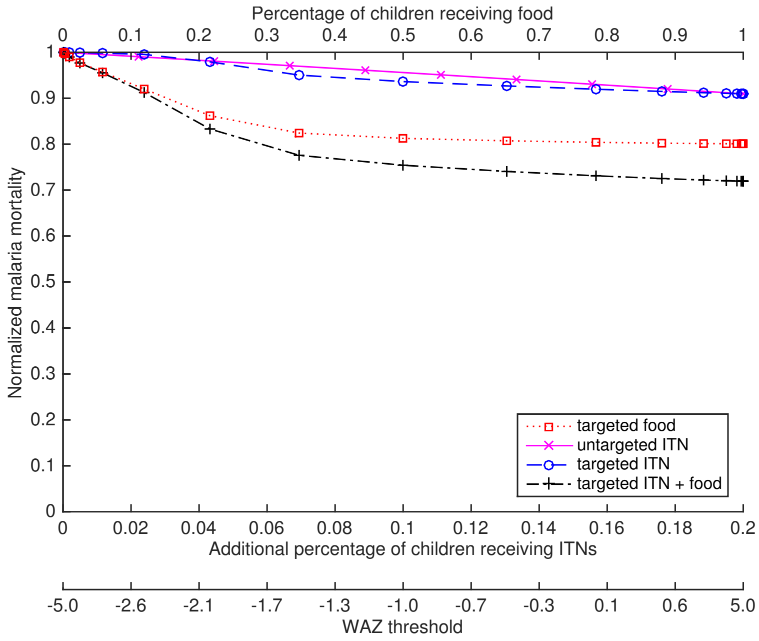

(b)

Figure 33: For the case of $k_1 = 0.08$, EIR $= 100$ (hyperendemic) and 80% baseline ITN coverage of children, which generates a clinical malaria prevalence of 0.424 in children at baseline, **(a)** the proportion of children with clinical malaria for a given coverage of a given policy, divided by the proportion of children with clinical malaria in the intervention-free case, and **(b)** the proportion of children who die from malaria for a given coverage of a given policy, divided by the proportion of children who die from malaria in the intervention-free case. The bottom of both figures gives the WAZ threshold that corresponds to a given coverage for the targeted policies (e.g., for the targeted ITN policy, ITNs are given to children with WAZ values below the threshold).

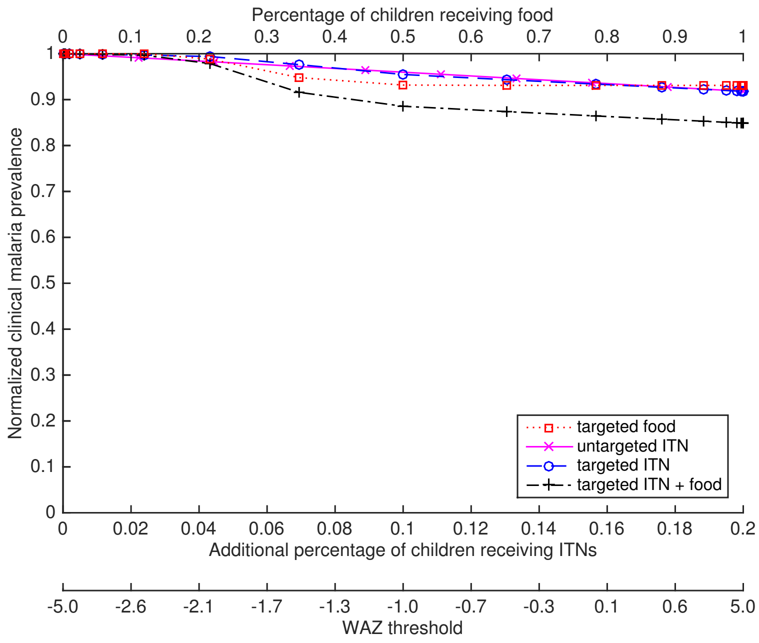

(a)

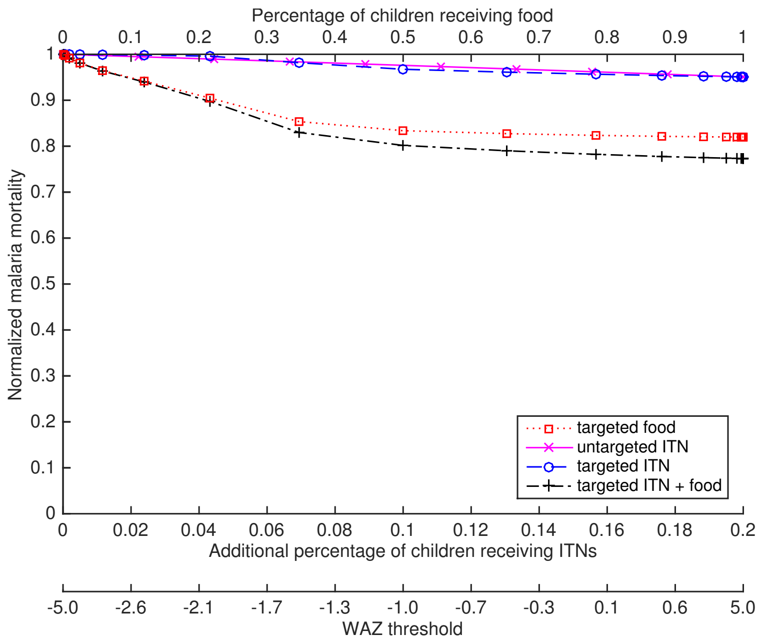

(b)

Figure 34: For the case of $k_1 = 0.08$, EIR $= 500$ (hyperendemic) and 80% baseline ITN coverage of children, which generates a clinical malaria prevalence of 0.558 in children at baseline, **(a)** the proportion of children with clinical malaria for a given coverage of a given policy, divided by the proportion of children with clinical malaria in the intervention-free case, and **(b)** the proportion of children who die from malaria for a given coverage of a given policy, divided by the proportion of children who die from malaria in the intervention-free case. The bottom of both figures gives the WAZ threshold that corresponds to a given coverage for the targeted policies (e.g., for the targeted ITN policy, ITNs are given to children with WAZ values below the threshold).
